# Supplementary material for: Benchmarking ChatGPT-4 on a radiation oncology in-training exam and Red Journal Gray Zone cases: potentials and challenges for ai-assisted medical education and decision making in radiation oncology
Source: Front Oncol. 2023 Sep 14;13:1265024. doi: 10.3389/fonc.2023.1265024 (PMC10543650; doi:10.3389/fonc.2023.1265024)
Supplement: Supplementary file 1 [file DataSheet_1.zip › ACR TXIT evaluation/ACR TXIT - Table of Specifications.pdf]

TXIT™ TABLE OF SPECIFICATIONS V. 042921

		Major Domain	Sub-Domain	% of Items
**1**		**Statistics**		**5%**
	1.1		Study design	
	1.2		Definitions of statistical terms	
	1.3		General interpretation and analysis	
	1.4		Survival curves	
	1.5		Specificity and sensitivity	
	1.6		Tests of significance	
	1.7		Phase III studies (randomized)	
	1.8		Retrospective trials and historical controls	
	1.9		Phase I and II studies (nonrandomized case control studies)	
	1.10		Multiple trials and meta-analysis	
**2**		**Bone and Soft Tissue**		**1.5%**
	2.1		Soft tissue sarcoma, including brachytherapy	
	2.2		Bone tumors (other than listed below)	
	2.3		Ewing sarcoma and osteosarcoma	
	2.4		Chondrosarcoma and chordoma	
	2.5		Desmoid tumor	
	2.6		Bone metastases	
	2.7		Benign entities, including heterotopic ossification	
	2.8		Kaposi's sarcoma	
	2.9		Non H+N skin cancers	
**3**		**Breast**		**8%**
	3.1		Early-stage breast cancer	
	3.2		Ductal carcinoma in situ (DCIS), Paget's disease, and lobular carcinoma in situ (LCIS)	
	3.3		Metastatic breast cancer	
	3.4		Locally advanced breast cancer, including neoadjuvant therapy and pathologic response	
	3.5		Inflammatory breast cancer	
	3.6		Recurrent breast cancer	
	3.7		Hypofractionation, APBI, and brachytherapy	
	3.8		Post-mastectomy radiation therapy	
	3.9		Axillary management, including micrometastases	
	3.10		Mammography screening, risk factors, genetics, and biomarkers	
	3.11		OARs, toxicity, and RT techniques	
	3.12		Anatomy and staging	

**4**		**CNS and Eye**		**6.5%**
	4.1		Brain metastasis and leptomeningeal disease	
	4.2		SRS Brain	
	4.3		Meningioma	
	4.4		Pituitary	
	4.5		Low grade gliomas	
	4.6		High grade gliomas	
			CNS germ cell tumors	
			Chordoma/chondrosarcoma	
			Spine radiosurgery	
	4.9		CNS lymphoma	
			Vestibular schwannoma	
	4.12		Eye diseases: lymphoma, melanoma, benign conditions (e.g. pterygium, Graves disease), including eye plaques for melanoma	
	4.10		Anatomy	
	4.14		OARs and toxicity	
**5**		**Gastrointestinal (GI)**		**7%**
	5.1		Esophagus	
	5.2		Stomach	
	5.3		Anus	
	5.4		Pancreas	
	5.5		Biliary tract	
	5.6		Liver, including SBRT	
	5.7		Colon and rectum	
	5.8		OARs, toxicity, and RT techniques	
	5.9		Systemic therapy, risk factors, anatomy, and staging	
**6**		**Genitourinary (GU)**		**7%**
	6.1		Prostate, including brachytherapy	
	6.2		Kidney and renal pelvis, ureter, and urethra	
	6.3		Testes: seminoma and non-seminoma	
	6.4		Bladder	
	6.5		Penis	
	6.6		OARs, toxicity, and RT techniques	
	6.7		ADT and systemic therapy, staging, risk factors, genetics, screening, anatomy, and management of metastases, including radioisotopes	

**7**		**Gynecology**		**7%**
	7.1		Endometrium and uterus	
	7.2		Vagina and vulva	
	7.3		Fallopian tube, ovary, and urethra	
	7.4		OARs, toxicity, and RT techniques	
	7.5		Uterine cervix	
	7.6		Brachytherapy	
	7.7		Systemic therapy, staging, risk factors, genetics, screening, and anatomy	
**8**		**Head, Neck and Skin**		**8%**
	8.1		Nasopharynx	
	8.2		Oral cavity (RMT and OT)	
	8.3		Oropharynx	
	8.4		Salivary gland	
	8.5		Larynx (supraglottic, larynx, subglottic)<br>Hypopharynx	
	8.6		Unknown primary	
	8.7		Thyroid gland<br>Paranasal sinuses	
	8.8		Skin cancers	
	8.9		Anatomy, including syndromes, and staging	
	8.10		Systemic therapy, including chemoradiation	
	8.11		Risk factors: tobacco, alcohol, HPV	
	8.12		OARs, toxicity, and RT techniques	
**9**		**Lung and Mediastinum**		**7%**
	9.1		SCLC/ Prophylactic Cranial Irradiation	
	9.2		Early Stage NSCLC	
	9.3		Advanced NSCLC	
	9.4		Palliation	
	9.5		Systemic therapy, risk factors, anatomy, and staging	
	9.6		OARs, toxicity, and RT techniques	
	9.7		Thymoma	
	9.8		Mesothelioma	

**10**		**Lymphoma and Leukemia**		**6.5%**
	10.1		Diffuse Large B cell	
	10.2		Follicular	
	10.3		MALT or other NHL	
	10.4		Multiple myeloma and/or plasmacytomas	
	10.5		Hodgkin disease	
	10.6		Leukemia	
	10.7		Total body and/or skin irradiation/transplant	
	10.8		Radioimmunotherapy	
	10.9		OARs, toxicity, and RT techniques	
**11**		**Pediatrics**		**6.5%**
	11.1		Hodgkin disease	
	11.2		Neuroblastoma	
	11.3		Wilms	
	11.4		Rhabdomyosarcoma	
	11.5		Ewing sarcoma	
	11.6		Leukemias	
	11.7		Craniopharyngioma	
	11.8		Medulloblastoma	
	11.9		Ependymoma	
	11.10		Other pediatric CNS tumors (DIPG, pilocytic astrocytoma, pedi HGG, ATRT)	
	11.11		OARs and RT techniques	
	11.12		Late effects	
**12**		**Biology**		**15%**
	12.1		Interaction of Radiation with Biological Systems	
	12.2		Molecular Mechanisms of DNA Damage	
	12.3		Molecular Mechanisms of DNA Repair	
	12.4		Chromosome and Chromatid Damage	
	12.5		Mechanisms of Cell Death	
	12.6		Cell and Tissue Survival Assays	
	12.7		Models of Cell Survival	
	12.8		Modifiers of Cell Survival: RBE and LET	
	12.9		Modifiers of Cell Survival: Oxygen Effect	
	12.10		Modifiers of Cell Survival: Cellular Recovery	
	12.11		Tumor Biology	
	12.12		Normal and Tumor Cell Kinetics	
	12.13		Molecular Signaling	
	12.14		Cancer Biology	
	12.15		Total Body Irradiation	

	12.16		Clinically Relevant Normal Tissue Responses to Radiation	
	12.17		Mechanisms of Normal Tissue Radiation Responses	
	12.18		Therapeutic Ratio	
	12.19		Time, Dose and Fractionation Effects	
	12.20		Nonstandard Dose Delivery Systems	
	12.21		Chemotherapy Agents and Radiation Therapy	
	12.22		Radiosensitizers, Radioprotectors, and Bioreductive Drugs	
	12.23		Hyperthermia	
	12.24		Radiation Carcinogenesis	
	12.25		Heritable Effects of Radiation	
	12.26		Teratogenesis: Effects on the Embryo and Fetus	
	12.27		Radiation Protection	
**13**		**Physics**		**15%**
	13.1		Atomic and nuclear structure	
	13.2		Production of photons and electrons	
	13.3		Treatment machines and generators; simulators and simulation tools	
	13.4		Radiation interactions	
	13.5		Radiation beam quality and dose	
	13.6		Radiation measurement and calibrations	
	13.7		Photons and x-ray characteristics of dosimetry	
	13.8		Electron beam characteristics and planning	
	13.9		External beam QA	
	13.10		Informatics	
	13.11		Brachytherapy, radiation protection and shielding	
	13.12		Imaging for Radiation Oncology	
	13.13		3D CRT Including ICRU concepts and beam related biology	
	13.14		Assessment of patient setup and verification	
	13.15		IMRT	
	13.16		Special procedures	
	13.17		Particle therapy, including proton therapy	
				**100%**
