# Supplementary material for: Benchmarking ChatGPT-4 on a radiation oncology in-training exam and Red Journal Gray Zone cases: potentials and challenges for ai-assisted medical education and decision making in radiation oncology
Source: Front Oncol. 2023 Sep 14;13:1265024. doi: 10.3389/fonc.2023.1265024 (PMC10543650; doi:10.3389/fonc.2023.1265024)
Supplement: Supplementary file 2 [file DataSheet_2.zip › Red journal gray zone evaluation/Case10_with_GPT_response.pdf]

GRAY ZONE

CME ASTRO

INTERNATIONAL JOURNAL OF
RADIATION ONCOLOGY • BIOLOGY • PHYSICS

www.redjournal.org

Exploring the Role of Resection Post-Radiation Therapy in Gliomas

Daphne B. Scarpelli, BS, and Jerry J. Jaboin, MD, PhD

Department of Radiation Medicine, Oregon Health & Science University, Portland, Oregon

A 45-year-old man was incidentally found to have a T2 hyperintense expansile anterior right frontal lobe mass during evaluation for pansinusitis. The patient underwent craniotomy with partial resection of the T2 hyperintense mass with pathology consistent with a diffuse World Health Organization grade 2 glioma, positive IDH1 mutation and 1p/q19 codeletion. The treatment team deferred completion resection. They proceeded with conventionally fractionated radiation therapy (54 Gy in 1.8 Gy daily fractions) with concurrent and adjuvant temozolomide recommended per CATNON and RTOG 9802.[1,2] After 5 of 12 cycles of adjuvant temozolomide, post-chemoradiation magnetic resonance imaging was notable for a decreased expansile T2/FLAIR mass and persistent diffuse signal abnormality in the right frontal lobe. The patient's case was reviewed for a second opinion on maximally completion resection.

Question

1. For a 45-year-old with good Karnofsky performance status who has completed concurrent chemoradiation therapy for a World Health Organization grade 2 glioma (1DH1 mutation and 1p/q19 codeletion), would you consider interval maximally safe resection?

References

1. van den Bent MJ, Baumert B, Erridge SC, et al. Interim results from the CATNON trial (EORTC study 26053-22054) of treatment with concurrent and adjuvant temozolomide for 1p/19q non-co-deleted anaplastic glioma: A phase 3, randomised, open-label intergroup study. *Lancet* 2017;390:1645–1653.
2. Laack NN, Sarkaria JN, Buckner JC. Radiation therapy oncology group 9802: Controversy or consensus in the treatment of newly diagnosed low-grade glioma? *Semin Radiat Oncol* 2015;25:197–202.

*Corresponding author: Jerry J. Jaboin, MD, PhD; E-mail: jaboin@ohsu.edu
Disclosures: none.
Note—CME is available for this feature as an ASTRO member benefit. To access, visit https://academy.astro.org.

Int J Radiation Oncol Biol Phys, Vol. 113, No. 1, pp. 11, 2022
0360-3016/$ - see front matter © 2021 Elsevier Inc. All rights reserved.
https://doi.org/10.1016/j.ijrobp.2021.03.013

INTERNATIONAL JOURNAL OF
RADIATION ONCOLOGY • BIOLOGY • PHYSICS
www.redjournal.org

GRAY ZONE EXPERT OPINION

Patience Is a Virtue

The imaging findings described after completion of concurrent chemoradiation and part of maintenance temozolomide (TMZ) overall suggest a decreased tumor burden but with some persistent signal abnormality, possibly representing residual or recurrent disease. Despite these findings, our recommendation would be continued observation with completion of scheduled maintenance TMZ. This patient[1] fits the most favorable molecular cohort in Radiation Therapy Oncology Group (RTOG)-9802 with isocitrate dehydrogenase (IDH) mutation and 1p/19q codeletion and, although the lack of upfront gross total resection (GTR) is suboptimal, the majority of patients (90%) in RTOG-9802 did not have a GTR. Post hoc analysis of RTOG-9802 showed excellent outcomes in patients with IDHmut/1p19q codeletion, showing a 5-year progression-free survival of nearly 90% for this cohort.[2] Therefore, early failure would be unlikely, and given the proven survival advantage of adjuvant chemotherapy, completion of the remainder of the planned TMZ is warranted.

If significant concern remained, we would recommend additional imaging with either magnetic resonance perfusion or positron emission tomography/magnetic resonance imaging to discern possible pseudoprogression (seen in approximately 20% of patients with low-grade glioma) from actual progression. Washington University reported their experience with patients with low-grade glioma with suspected progression, and 90% of suspicious findings ultimately were found to be pseudoprogression.[3] Of the 10 patients in their cohort who ultimately underwent surgery for reresection for progression, 80% had treatment effect with no evidence of residual or recurrent tumor. In lieu of clear evidence of progression (eg, new symptoms or radiographic evidence of progression on serial imaging), we would be hesitant to pursue surgical intervention because this would likely be low yield and without clear benefit to the patient.

Craig S. Schneider, MD, PhD
Christopher D. Willey, MD, PhD
Department of Radiation Oncology
The University of Alabama at Birmingham
Birmingham, Alabama

Int J Radiation Oncol Biol Phys, Vol. 113, No. 1, pp. 12−13, 2022
0360-3016/$ - see front matter © 2021 Elsevier Inc. All rights reserved.

Disclosures: C.D.W. is a part-time consultant for LifeNet Health, has received honoraria from Varian Medical Systems, and receives grant funding from NIH, AACR-Novocure and Varian.

References

1. Scarpelli DS, Jaboin JJ. Exploring the role of resection post-radiation therapy in gliomas. *Int J Radiat Oncol Biol Phys* 2022;113:11.
2. Bell EH, Zhang P, Shaw EG, et al. Comprehensive genomic analysis in NRG Oncology/RTOG 9802: A phase III trial of radiation versus radiation plus procarbazine, lomustine (CCNU), and vincristine in high-risk low-grade glioma. *J Clin Oncol* 2020;38:3407–3417.
3. Lin AL, White M, Miller-Thomas MM, et al. Molecular and histologic characteristics of pseudoprogression in diffuse gliomas. *J Neurooncol* 2016;130:529–533.

https://doi.org/10.1016/j.ijrobp.2021.03.041

Back to the OR

Low-grade gliomas (LGGs) represent an important source of morbidity and mortality in young adults. Upfront maximal safe resection is the standard of care, with adjuvant therapy used in high-risk patients. The association of molecular markers (IDH, ATRX, 1p/19q) and histology is used to stratify tumors into molecular subgroups. The 45-year-old patient highlighted[1] had a World Health Organization grade 2, IDH-mutated, 1p/19q codeleted glioma. Such patients invariably recur in the surgical cavity, and indeed, studies demonstrate that resection extent correlates with progression-free survival and overall survival (OS). Unfortunately, this patient did not receive initial gross total resection and immediately proceeded with adjuvant chemoradiation and temozolomide. Follow-up imaging demonstrated persistent disease in the right frontal lobe.

The question posed was whether further surgery should be employed. A retrospective study of 52 patients with recurrent LGG by Ramakrishna et al[2] found that the presence of any residual tumor after (first or second) surgery was associated with decreased OS. They also found that the

use of upfront radiation and pathology at recurrence affected OS. With respect to progression-free survival after a surgically treated recurrence, significant differences were observed with higher grade tumors, extent of resection at time of first recurrence, residual disease after first operation, resectability, and Karnofsky performance status score. Such data and others[3] highlight that patients with recurrent LGG are likely to benefit from additional surgery. Notably, such patients had low risk of surgical complications, performance status decrements, or neurologic sequelae. Thus, we would enthusiastically support reresection.

Kim C. Ohaegbulam, MD, PhD
Ravi A. Chandra, MD, PhD
Department of Radiation Medicine
Oregon Health & Science University
Portland, Oregon

Disclosures: none.

References

1. Scarpelli DS, Jaboin JJ. Exploring the role of resection post-radiation therapy in gliomas. *Int J Radiat Oncol Biol Phys* 2022;113:11.
2. Ramakrishna R, Hebb A, Barber J, Rostomily R, Silbergeld D. Outcomes in reoperated low-grade gliomas. *Neurosurgery* 2015;77:175–184.
3. Uppstrom TJ, Singh R, Hadjigeorgiou G, Magge R, Ramakrishna R. Repeat surgery for recurrent low-grade gliomas should be standard of care. *Clin Neurol Neurosurg* 2016;151:18–23.

https://doi.org/10.1016/j.ijrobp.2021.07.010

Just Because You *Can* Does Not Mean That You *Should*. . .

The optimal management of patients with low-grade glioma remains controversial. There currently are no randomized studies addressing the question of re-resection after adjuvant chemotherapy and radiation therapy (RT) for patients with glioma, World Health Organization grade 2, IDH1-mutant, 1p/19q codeletion, known as *molecular oligodendroglioma*, following initial subtotal resection. These patients typically have a long expected survival and good prognosis.

The first rationale for re-resection is to prevent the residual mass from acting as a source for malignant transformation (MT) to a high-grade glioma. However, the suggestion that RT results in MT has not been supported by data from the European Organization for Research and Treatment of Cancer 22845 (“nonbelievers”) trial, where both arms (early vs delayed RT) had similar rates of MT (approximately 70% at re-resection).[1] Evidence suggests that chemotherapy, particularly temozolomide, contributes to MT.[2,3] As such, our and other institutions typically would recommend treatment according to Radiation Therapy Oncology Group 9802, with sequential RT and procarbazine, lomustine, and vincristine rather than temozolomide.[4] Whether early reresection limits the risk of MT in patients is currently uncertain.

Other reasons for re-resection do not apply to this patient. This patient is asymptomatic, but one would recommend re-resection for other patients when the residual tumor causes mass effect, symptoms such as refractory seizures, and/or herniation. Additionally, re-resection would be performed to avoid adjuvant treatment altogether in cases in which resection would not result in unacceptable neurologic deficit and would render the patient low risk by Radiation Therapy Oncology Group 9802 criteria (<40 years old and with gross total resection).

Re-resection of this right frontal tumor[5] would be technically feasible. The benefits of such surgery, however, are unclear. Just because one can, does not mean one should. I would recommend continued serial observation for this patient.

Joshua S. Silverman, MD, PhD
Department of Radiation Oncology
NYU Langone Health
Grossman School of Medicine
New York, New York

Disclosures: none

References

1. van den Bent MJ, Afra D, de Witte O, et al. Long-term efficacy of early versus delayed radiotherapy for low-grade astrocytoma and oligodendroglioma in adults: The EORTC 22845 randomised trial. *Lancet* 2005;366:985–990.
2. Tom MC, Park DYJ, Yang K, et al. Malignant transformation of molecularly classified adult low-grade glioma. *Int J Radiat Oncol Biol Phys* 2019;105:1106–1112.
3. Choi S, Yu Y, Grimmer MR, et al. Temozolomide-associated hypermutation in gliomas. *Neuro Oncol* 2018;20:1300–1309.
4. Buckner JC, Shaw EG, Pugh SL, et al. Radiation plus procarbazine, CCNU and vincristine in low-grade glioma. *New Engl J Med* 2016;374:1344–1355.
5. Scarpelli DS, Jaboin JJ. Exploring the role of resection post-radiation therapy in gliomas. *Int J Radiat Oncol Biol Phys* 2022;113:11.

https://doi.org/10.1016/j.ijrobp.2022.01.018

ChatGPT-4: Surgical Reevaluation and Adjuvant Therapy

User: *You are an expert radiation oncologist from an academic center.*

Please give your recommendation on the following case:

"…"

Please give a specific recommendation of your most favored therapeutic approach in this patient case based on the available information and your reasoning for it.

ChatGPT-4: Based on the available information, my recommendation for this patient would be to consider a multidisciplinary approach that includes a thorough reevaluation of the possibility of maximal safe resection of the residual tumor, followed by continuing adjuvant temozolomide, and potentially the addition of tumor-treating fields (TTF) if deemed appropriate. This recommendation is based on the following reasoning:

Maximal safe resection: The patient initially underwent partial resection of the tumor, and the treatment team deferred completion resection. However, given the decrease in the expansile T2/FLAIR mass after chemoradiation, it may be worth reevaluating the feasibility of achieving a more complete resection. Maximal safe resection of low-grade gliomas has been associated with improved overall survival and progression-free survival in multiple studies. If the multidisciplinary team, including a neurosurgeon, deems it safe and feasible, this could be a beneficial step for the patient.

Continuing adjuvant temozolomide: The patient is currently on their 5th cycle of adjuvant temozolomide, which is in line with the treatment recommendations from the CATNON and RTOG 9802 trials. These trials demonstrated that adding temozolomide to radiation therapy in patients with grade 2 gliomas and specific molecular markers, such as IDH1 mutation and 1p/19q codeletion, led to improved overall survival. Given the patient's positive response to treatment so far, it would be reasonable to continue with the remaining cycles of adjuvant temozolomide.

Consideration of tumor-treating fields (TTF): TTF is a novel treatment modality that uses low-intensity, alternating electric fields to disrupt cell division in cancer cells. In the EF-14 trial, the addition of TTF to maintenance temozolomide was shown to improve overall survival and progression-free survival in patients with newly diagnosed glioblastoma. While the trial specifically focused on glioblastoma, TTF may still be considered as a potential therapeutic option for patients with grade 2 gliomas, particularly if the multidisciplinary team believes it could be beneficial in this specific case.

In summary, I recommend a thorough reevaluation of the possibility of maximal safe resection of the residual tumor, continuing adjuvant temozolomide, and potentially considering the addition of TTF if deemed appropriate by the multidisciplinary team. The patient's case should be regularly discussed and monitored by a team of experts, including a neurosurgeon, radiation oncologist, and medical oncologist, to ensure the most effective and personalized treatment plan.

User: *For a 45-year-old with good Karnofsky performance status who has completed concurrent chemoradiation therapy for a*

World Health Organization grade 2 glioma (1DH1 mutation and 1p/q19 codeletion), would you consider interval maximally safe resection?

ChatGPT-4: Yes, for a 45-year-old patient with good Karnofsky performance status who has completed concurrent chemoradiation therapy for a World Health Organization grade 2 glioma with IDH1 mutation and 1p/19q codeletion, I would consider interval maximally safe resection.

The rationale for considering interval maximally safe resection is as follows:

a. Improved overall survival and progression-free survival: Multiple studies have demonstrated that maximal safe resection of low-grade gliomas is associated with improved overall survival and progression-free survival. Achieving a more complete resection could potentially lead to better outcomes for this patient.

b. Good performance status: The patient's good Karnofsky performance status suggests that they may be able to tolerate the surgery and recover well from it. This is an important factor when considering additional surgical interventions.

c. Response to chemoradiation: As the patient's post-chemoradiation MRI showed a decreased expansile T2/FLAIR mass and persistent diffuse signal abnormality in the right frontal lobe, it is possible that the residual tumor may now be more amenable to resection. A reevaluation of the surgical feasibility and safety is warranted in this case.

Before proceeding with interval maximally safe resection, it is crucial to discuss the patient's case within a multidisciplinary team that includes a neurosurgeon, radiation oncologist, and medical oncologist to ensure the most effective and personalized treatment plan. The potential benefits of additional surgery must be carefully weighed against the risks and potential complications. Additionally, it is essential to closely monitor the patient's response to treatment and adjust the therapeutic approach as needed.

User: *Please summarize the other experts' recommendations in short sentences.*

ChatGPT-4:

Expert 1: Recommends continued observation with completion of scheduled maintenance temozolomide, as the patient has favorable molecular markers and excellent outcomes are expected. Additional imaging may be considered to discern possible pseudoprogression from actual progression.

Expert 2: Supports reresection, as studies demonstrate that resection extent correlates with progression-free survival and overall survival. Patients with recurrent low-grade gliomas are likely to benefit from additional surgery with low risk of surgical complications or neurologic sequelae.

Expert 3: Recommends continued serial observation, as the benefits of re-resection are unclear in this case. The patient is asymptomatic and has a long expected survival and good prognosis, so the rationale for re-resection is uncertain.

User: *As an expert radiation oncologist, which of the above experts' recommendation do you think the most proper?*

ChatGPT-4: As an expert radiation oncologist, I believe the most proper approach for the patient described above would be a combination of Expert 1's and Expert 2's recommendations. This involves:

Continuing observation and completing the scheduled maintenance temozolomide, as the patient has favorable molecular markers and has shown a decrease in the tumor burden after chemoradiation.

Reevaluating the possibility of maximal safe resection of the residual tumor. Studies demonstrate that the extent of resection correlates with progression-free survival and overall survival. This reevaluation should involve a multidisciplinary team, including a neurosurgeon, to determine if it is safe and feasible to achieve a more complete resection, taking into consideration the patient's good Karnofsky performance status.

The reason for combining these two recommendations is that they emphasize both the importance of completing the ongoing adjuvant therapy and the potential benefits of considering maximal safe resection if deemed appropriate by the multidisciplinary team. This approach aims to balance the potential benefits of additional surgery with the risks and uncertainties associated with it while ensuring the patient receives the planned and proven effective adjuvant therapy.

User: *At the beginning, you said that "my recommendation for this patient would be to consider a multidisciplinary approach that includes a thorough reevaluation of the possibility of maximal safe resection of the residual tumor, followed by continuing adjuvant temozolomide, and potentially the addition of tumor-treating fields (TTF) if deemed appropriate." In this case, which expert's recommendation is your initial suggestion closest to?*

ChatGPT-4: My initial recommendation is closest to a combination of Expert 1's and Expert 2's recommendations. This is because:

Like Expert 1, I emphasized the importance of continuing observation and completing the scheduled maintenance temozolomide, given the favorable molecular markers and the patient's response to chemoradiation.

Similar to Expert 2, I suggested reevaluating the possibility of maximal safe resection of the residual tumor, as studies have shown a correlation between the extent of resection and progression-free survival and overall survival.

In addition to these shared suggestions, I also mentioned the potential consideration of tumor-treating fields (TTF) if deemed appropriate by the multidisciplinary team. This recommendation is not explicitly mentioned by any of the three experts but could be an additional therapeutic option for this patient.

User: *After seeing the other experts' recommendations, are you going to update your own recommendation of your most favored therapeutic approach in this patient case? If so, what is your updated recommendation and which expert/experts is your update based on?*

ChatGPT-4: After reviewing the recommendations of the other three experts, I would update my initial recommendation slightly, while still maintaining a combination of Expert 1's and Expert 2's suggestions:

Updated Recommendation:

- Continue with observation and complete the scheduled maintenance temozolomide, as the patient has favorable molecular markers and has shown a decrease in the tumor burden after chemoradiation.

- Reevaluate the possibility of maximal safe resection of the residual tumor, involving a multidisciplinary team, including a neurosurgeon, to determine if it is safe and feasible to achieve a more complete resection, taking into consideration the patient's good Karnofsky performance status.

- I would no longer emphasize the potential addition of tumor-treating fields (TTF) as a primary recommendation in this case, given the other three experts did not mention it and the focus should remain on the management strategies with more established benefits for this specific patient population. However, TTF could still be considered as an option for discussion within the multidisciplinary team if the standard approaches are not providing the desired results or if further evidence emerges supporting its use in grade 2 gliomas.

This updated recommendation is based on a combination of Expert 1's and Expert 2's recommendations, as it acknowledges the importance of completing the ongoing adjuvant therapy while also considering the potential benefits of maximal safe resection if deemed appropriate by the multidisciplinary team.
