# Supplementary material for: Benchmarking ChatGPT-4 on a radiation oncology in-training exam and Red Journal Gray Zone cases: potentials and challenges for ai-assisted medical education and decision making in radiation oncology
Source: Front Oncol. 2023 Sep 14;13:1265024. doi: 10.3389/fonc.2023.1265024 (PMC10543650; doi:10.3389/fonc.2023.1265024)
Supplement: Supplementary file 2 [file DataSheet_2.zip › Red journal gray zone evaluation/Case14_with_GPT_response.pdf]

GRAY ZONE

CME ASTRO

INTERNATIONAL JOURNAL OF
RADIATION ONCOLOGY • BIOLOGY • PHYSICS

www.redjournal.org

Trim One's (E)Wings—How Much is Enough?

Boryana M. Eastman, MD, PhD, and Gabrielle M. Kane, MD

Department of Radiation Oncology, University of Washington School of Medicine, Seattle, Washington

Received Oct 25, 2020; Accepted for publication Nov 20, 2020

A 52-year-old woman presented with an enlarging firm perianal mass consistent with ischiorectal abscess. She underwent resection of a 5.5 × 3.5 × 3 cm mass abutting the external sphincter muscle. Final pathology was consistent with Ewing sarcoma (EWSR1-FLI1 fusion gene present) with positive margins and perineural invasion. Staging scans including computed tomography of the chest/abdomen/pelvis, and full-body positron emission tomography-computed tomography scan showed no evidence of metastatic disease.

She was started on systemic treatment with alternating cycles of vincristine, doxorubicin, and cyclophosphamide and ifosfamide and etoposide for total of 14 planned cycles (7 of vincristine, doxorubicin, and cyclophosphamide and 7 of ifosfamide and etoposide). Magnetic resonance imaging of the pelvis after cycle 2 of chemotherapy revealed no residual mass (Fig. 1). The patient was evaluated by colorectal surgery with recommendations for abdominal perineal resection and permanent colostomy and by radiation oncology with recommendations for definitive chemoradiation therapy.

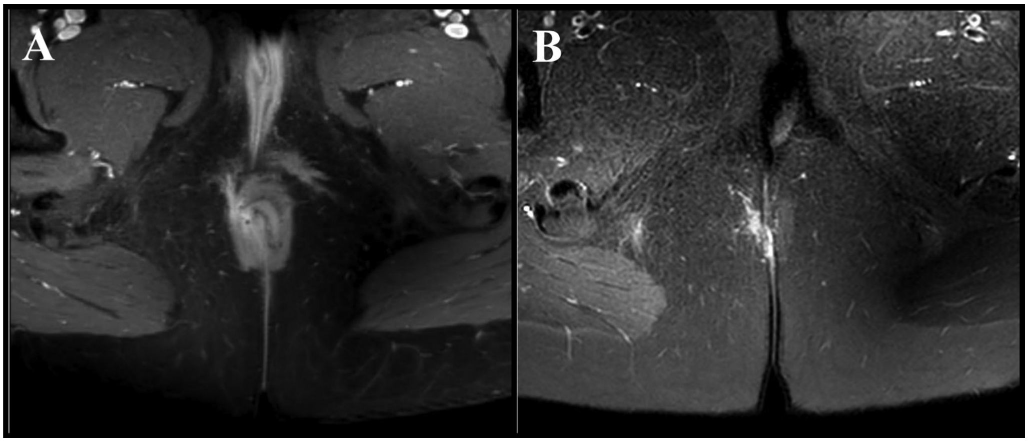

Fig. 1. Magnetic resonance imaging after resection and 2 cycles of chemotherapy. (A) Postcontrast T1-weighted axial image with fat saturation. (B) T2-weighted axial image with fat saturation.

Questions

1. Would surgical re-resection or radiation lead to better local control of the patient's Ewing sarcoma?
2. Given the perineural invasion and the perianal location of the primary lesion, would a combined approach (ie, preoperative radiation followed by definitive resection) be preferred?

*Corresponding author: Boryana Eastman, MD, PhD; E-mail: beastman@uw.edu

Disclosures: none.

CME is available for this feature as an ASTRO member benefit, to access visit https://academy.astro.org.

Int J Radiation Oncol Biol Phys, Vol. 112, No. 2, pp. 278, 2022
0360-3016/$ - see front matter © 2020 Elsevier Inc. All rights reserved.
https://doi.org/10.1016/j.ijrobp.2020.11.063

INTERNATIONAL JOURNAL OF
RADIATION ONCOLOGY • BIOLOGY • PHYSICS
www.redjournal.org

GRAY ZONE EXPERT OPINIONS

Tipping the Scales Toward Single Modality

Local control for Ewing sarcoma is typically planned as a single modality, surgery with planned negative margins or definitive chemoradiation therapy. Combined modality (surgery with pre- and/or postoperative radiation therapy) has not demonstrated significant benefit for local control[1] or survival.[2] The modality of choice should be based on the likelihood of success (eg, the degree of confidence that a re-resection would provide the disease clearance of a traditional R0 resection), morbidity, and degree of disruption to systemic therapy, which we know is essential for both local control and overall outcomes (Fig. 1). Owing to the lack of clinical equipoise to address the local control question in a prospective randomized fashion, we rely on retrospective analyses subject to selection bias, where traditionally surgery is favored for "expendable bones" and definitive radiation therapy is often the modality of choice for locally advanced tumors. These retrospective analyses incorporate patients treated with varied chemotherapy regimens, including less-intensive regimens, and it is known that local control can be influenced by the intensity of systemic therapy. Importantly, current data do not support using a different approach for extraskeletal Ewing sarcoma, for which a combined-modality soft tissue sarcoma approach should not be applied. We would consider the patient's[3] non-oncologic positive margin surgery before chemotherapy to be analogous to a biopsy and would not use this to guide our recommendations. The role of perineural invasion on local control is not well described, and we would not adjust our local control planning. Considering all these factors, we would anticipate definitive chemoradiation therapy as the approach of choice.

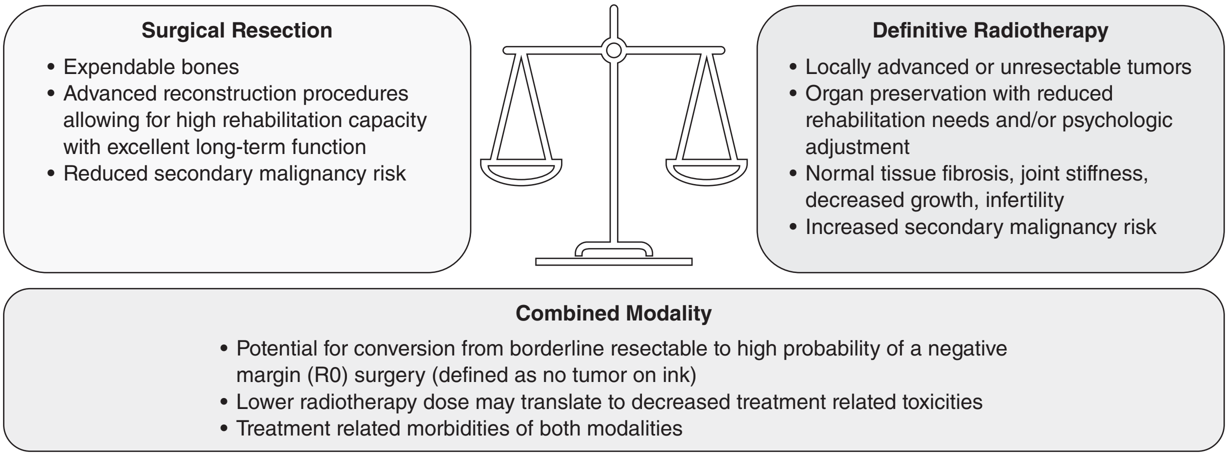

Fig. 1. Weighing the balance of informed discussion of local control treatment strategies for Ewing sarcoma.

Int J Radiation Oncol Biol Phys, Vol. 112, No. 2, pp. 279−281, 2022
0360-3016/$ - see front matter Published by Elsevier Inc.

Stephanie K. Schaub, MD[1]
Catherine M. Albert, MD[2]
[1]*Department of Radiation Oncology*
University of Washington
Seattle, Washington
[2]*Department of Hematology-Oncology, Seattle Children's Hospital*
University of Washington
Seattle, Washington

Disclosures: None.

References

1. Ahmed SK, Robinson SI, Arndt CAS, et al. Pelvis Ewing sarcoma: Local control and survival in the modern era. *Pediatr Blood Cancer* 2017;64.
2. DuBois SG, Krailo MD, Gebhardt MC, et al. Comparative evaluation of local control strategies in localized Ewing sarcoma of bone: A report from the Children's Oncology Group. *Cancer* 2015;121:467–475.
3. Eastman BM, Kane GM. Trim one's (E)wings—how much is enough? *Int J Radiat Oncol Biol Phys* 2022;112:278.

https://doi.org/10.1016/j.ijrobp.2021.04.038

Matthew B. Spraker, MD, PhD[1]
Mia C. Weiss, MD[2]
[1]*Department of Radiation Oncology*
Washington University in St Louis
St Louis, Missouri
[2]*Department of Medicine, Division of Hematology and Oncology*
Washington University in St Louis
St Louis, Missouri

Disclosures: None.

References

1. DuBois SG, Krailo MD, Gebhardt MC, et al. Comparative evaluation of local control strategies in localized Ewing sarcoma of bone: A report from the Children's Oncology Group. *Cancer* 2015;121:467–475.
2. Eastman BM, Kane GM. Trim one's (E)wings—how much is enough? *Int J Radiat Oncol Biol Phys* 2022;112:278.
3. Orr WS, Denbo JW, Billups CA, et al. Analysis of prognostic factors in extraosseous Ewing sarcoma family of tumors: Review of St. Jude Children's Research Hospital experience. *Ann Surg Oncol* 2012;19:3816–3822.

https://doi.org/10.1016/j.ijrobp.2021.04.036

Tilting the Balance, and Gantry, Towards Preservation of Function

Balancing Function Preservation and Local Control in Localized Ewing Sarcoma

Standard treatment for localized Ewing sarcoma includes chemotherapy and local therapy. Surgery and radiation therapy are effective local treatment options, but there has been no randomized trial comparing them. A pooled analysis of 1444 patients from Ewing sarcoma randomized trials found that the risk of local failure was higher for patients treated with radiation therapy, but all patients had similar event-free and overall survival.[1] As discussed by the authors,[2] this "reflects the relatively low contribution of local failure to overall disease failure in Ewing sarcoma" and validates radiation therapy as a reasonable alternative to morbid surgery. This study also found no benefit of surgery plus radiation therapy compared with either modality alone. Unless tumor shrinkage would allow for a less morbid surgery, teams should select one or the other. Clinical factors including patient age, expected margin status, and function preservation should be considered.

One consideration is that this is an extraosseous Ewing sarcoma. Although these tumors are less well studied than skeletal Ewing sarcoma, case series suggest that their behavior is similar,[3] and the same concepts can likely be applied as with skeletal Ewing sarcomas.

Radiation therapy should be selected in this 52-year-old woman facing a permanent colostomy with surgery.

Extraosseous, Always Personal

Adult-onset extraosseus Ewing sarcoma diagnosed via non-oncologic resection is a challenging beginning to a disease with an already circuitous treatment paradigm. In cases of complete excision before induction chemotherapy, non−organ-enclosed tumors are considered appropriately removed only when a 2 cm margin is achieved.[1]

Surgery (with or without radiation therapy) versus radiation as local control therapy is dictated by the feasibility of obtaining adequate margins and maintaining the patient's functional integrity. No prospective, randomized study of surgery versus radiation exists. In review of the intergroup trials, however, the presumed blanket superiority of surgery over radiation may be questionable. Even when not controlling for disease site resectability, radiation therapy may be an equivalent option for some, if not all, patients with Ewing sarcoma in regard to local control[3] and, in turn, comparable overall survival.

The options of radiation or surgery are typically reviewed among all treatment physicians, and if surgery is thought appropriate, it is offered. However, in applying the research data to specific patients with Ewing sarcoma, if radiation therapy is better able to minimize toxicity at a specific anatomic site where resection could be detrimental to the patient, it should be offered as a viable local control therapeutic option.

When reviewing treatment options,[2] resuming consolidative chemotherapy should also be taken into account.

Timely resumption of chemotherapy with less than 6 weeks between cycles should be built into local control management.[2] The chemotherapy may be given concurrently with radiation therapy as primary local control therapy or administered with a radiation boost.

Krisha J. Howell, MD, Associate Professor
Fox Chase Cancer Center
Philadelphia, PA 19111

Disclosures: none.

References

1. COG AEWS 1031: A phase III randomized trial of adding vincristine-topotecan-cyclophospamide to standard chemotherapy in initial treatment of non-metastatic Ewing sarcoma. Available at: childrensoncologygroup.com. Accessed February 27, 2019.
2. Eastman BM, Kane GM. Trim one's (E)wings—how much is enough? *Int J Radiat Oncol Biol Phys* 2022;112:278.
3. Ahmed S. Identification of patients with localized Ewing sarcoma at higher risk for local failure: A report from the Children's Oncology Group. *Int J Radiat Oncol Biol Phys* 2017;99:1286–1294.

https://doi.org/10.1016/j.ijrobp.2021.09.016

ChatGPT-4: Combined Approach of Neoadjuvant Chemoradiation Therapy and Tailored Surgical Resection

User: *You are an expert radiation oncologist from an academic center.*

Please give your recommendation on the following case:

"…"

Please give a specific recommendation of your most favored therapeutic approach in this patient case based on the available information and your reasoning for it.

ChatGPT-4: Based on the available information, my recommendation for this patient would be to proceed with neoadjuvant chemoradiation therapy followed by a reassessment for local control measures such as an abdominoperineal resection (APR) with colostomy if needed.

My reasoning for this approach is as follows:

- The patient's Ewing sarcoma is a rare and aggressive malignancy, and she has already demonstrated a good response to the initial two cycles of systemic chemotherapy. Continuing with the planned 14 cycles of chemotherapy is essential to provide optimal systemic control of the disease.

- The addition of neoadjuvant chemoradiation therapy would aim to further enhance local control and potentially facilitate a more conservative surgical approach if the tumor response is favorable. Chemoradiation has been shown to improve local control rates in other soft tissue sarcomas, and it might help in achieving negative surgical margins, reducing the risk of local recurrence.

- Following neoadjuvant chemoradiation therapy, the patient should be re-evaluated to assess the extent of tumor response. If there is a significant reduction in the tumor size and the risk of positive margins has decreased, a more conservative surgical approach, such as a wide local excision, might be considered instead of the more invasive APR with colostomy. However, if the tumor response is not as favorable or there is still a significant risk of positive margins, proceeding with an APR and colostomy would be appropriate to ensure adequate local control.

- The multidisciplinary team, including the colorectal surgeon and the radiation oncologist, should continue to work closely together to tailor the treatment plan according to the patient's response and preferences, weighing the benefits and risks of each approach.

In summary, my favored therapeutic approach for this patient would be to continue with the planned systemic chemotherapy, incorporate neoadjuvant chemoradiation therapy to improve local control, and reassess for appropriate local control measures based on the tumor response following chemoradiation therapy.

User: *Would surgical re-resection or radiation lead to better local control of the patient's Ewing sarcoma?*

ChatGPT-4: The choice between surgical re-resection and radiation for better local control in this patient's Ewing sarcoma depends on several factors, including the tumor's location, size, and response to neoadjuvant therapy, as well as the patient's overall health and preferences. Both surgical re-resection and radiation therapy can be effective in achieving local control, but their success depends on the specifics of the case.

Surgical re-resection can lead to better local control if it results in negative surgical margins, meaning that no cancer cells are found at the edge of the removed tissue. Achieving negative margins is crucial in reducing the risk of local recurrence. However, given the tumor's location in the perianal region and its proximity to the external sphincter muscle, a more aggressive surgery, such as abdominoperineal resection (APR) with colostomy, might be necessary to achieve negative margins, leading to a significant impact on the patient's quality of life.

Radiation therapy, on the other hand, can be a less invasive option for achieving local control, especially when used in combination with chemotherapy (chemoradiation). In some cases, chemoradiation can lead to substantial tumor shrinkage, making it easier to achieve negative surgical margins if surgery is still needed. However, radiation therapy also comes with potential side effects, such as damage to nearby healthy tissues and organs, which should be considered when making treatment decisions.

In this particular case, since the patient has already shown a good response to chemotherapy, neoadjuvant chemoradiation therapy may be a reasonable option to further enhance local control and potentially facilitate a more conservative surgical approach if the tumor response is favorable. After completing the neoadjuvant chemoradiation therapy, the patient should be re-evaluated to determine the most appropriate local control measure, taking into consideration the tumor response, patient preferences, and the potential risks and benefits of each approach. The multidisciplinary team, including the colorectal surgeon and the radiation oncologist, should work together to tailor the treatment plan to the patient's specific needs and preferences.

User: *Given the perineural invasion and the perianal location of the primary lesion, would a combined approach (ie, preoperative radiation followed by definitive resection) be preferred?*

ChatGPT-4: In this particular case, a combined approach of preoperative radiation (neoadjuvant chemoradiation therapy) followed by definitive resection might indeed be preferred due to the perineural invasion and perianal location of the primary lesion.

The presence of perineural invasion can increase the risk of local recurrence and suggests a more aggressive tumor behavior. Preoperative radiation therapy, especially when combined with chemotherapy, can help to reduce the tumor size, improve the likelihood of achieving negative surgical margins, and potentially eradicate microscopic disease in the perineural tissues, thereby improving local control.

The perianal location of the lesion poses a challenge in terms of achieving adequate surgical margins while preserving the patient's sphincter function and quality of life. Neoadjuvant chemoradiation therapy may increase the likelihood of a more conservative surgical approach if the tumor response is favorable, thus preserving the patient's anorectal function and avoiding the need for a permanent colostomy.

Following neoadjuvant chemoradiation therapy, the patient should be re-evaluated to determine the extent of tumor response and the most appropriate surgical approach. If there is a significant reduction in the tumor size and the risk of positive margins has decreased, a more conservative surgical approach, such as wide local excision, might be considered. However, if the tumor response is not as favorable or there is still a significant risk of positive margins, proceeding with an abdominoperineal resection (APR) and colostomy would be appropriate to ensure adequate local control.

In summary, given the perineural invasion and perianal location of the primary lesion, a combined approach of neoadjuvant chemoradiation therapy followed by definitive resection, tailored to the patient's tumor response and preferences, could be the preferred choice for optimizing local control and preserving the patient's quality of life.

User: *Please summarize the other experts' recommendations in short sentences.*

ChatGPT-4:

Expert 1's recommendation: They favor definitive chemoradiation therapy as the approach of choice for this patient, as they believe combined modality (surgery and radiation) has not demonstrated significant benefits for local control or survival in Ewing sarcoma.

Expert 2's recommendation: They suggest selecting radiation therapy in this patient to preserve function, as it is a reasonable alternative to morbid surgery, and they found no benefit of surgery plus radiation therapy compared to either modality alone.

Expert 3's recommendation: They emphasize that radiation therapy can be a viable local control therapeutic option in cases where it better minimizes toxicity and maintains patient functionality, and they stress the importance of timely resumption of chemotherapy in the treatment plan.

User: *As an expert radiation oncologist, which of the above experts' recommendation do you think the most proper?*

ChatGPT-4: As an expert radiation oncologist, I believe the recommendations from Expert 2 align most closely with my own assessment of the patient's case. Expert 2 suggests selecting radiation therapy for this patient to preserve function, as it serves as a reasonable alternative to morbid surgery. They found no benefit in combining surgery and radiation therapy compared to either modality alone.

In the context of the patient's perineural invasion and perianal location of the primary lesion, a combined approach of neoadjuvant chemoradiation therapy followed by a definitive resection tailored to the patient's tumor response and preferences could optimize local control and preserve the patient's quality of life. This approach is consistent with Expert 2's emphasis on balancing function preservation and local control.

It is important to note that the decision should be made in a multidisciplinary context, with input from the colorectal surgeon, radiation oncologist, and medical oncologist, and considering the patient's preferences and unique circumstances.

***User:** At the beginning, you said that "my recommendation for this patient would be to proceed with neoadjuvant chemoradiation therapy followed by a reassessment for local control measures such as an abdominoperineal resection (APR) with colostomy if needed." In this case, which expert's recommendation is your initial suggestion closest to?*

ChatGPT-4: My initial recommendation is closest to Expert 2's recommendation, which emphasizes the importance of balancing function preservation and local control in localized Ewing sarcoma. Both recommendations advocate for the use of radiation therapy in this case, considering the potential for preserving the patient's function while maintaining effective local control.

***User:** After seeing the other experts' recommendations, are you going to update your own recommendation of your most favored therapeutic approach in this patient case? If so, what is your updated recommendation and which expert/experts is your update based on?*

ChatGPT-4: After reviewing the three experts' recommendations, my updated recommendation for this patient case is to continue with the planned systemic chemotherapy and proceed with definitive radiation therapy as the primary local control strategy, with the aim to preserve function and minimize morbidity. This updated recommendation is primarily based on the insights provided by Expert 2.

My reasoning for this updated approach is as follows:

- The patient has already demonstrated a good response to the initial two cycles of systemic chemotherapy, and continuing with the planned 14 cycles of chemotherapy is essential for optimal systemic control.

- Definitive radiation therapy is a reasonable alternative to morbid surgery, especially given the perianal location of the tumor and the risk of permanent colostomy associated with aggressive surgery. Radiation therapy can help achieve local control while preserving the patient's anorectal function and quality of life.

- As Expert 2 highlighted, there is no clear evidence of additional benefit from combining surgery and radiation therapy compared to either modality alone. Therefore, it is reasonable

to choose one modality based on factors such as tumor location, patient age, expected margin status, and the potential for function preservation.

- It is essential to continue working closely with the multidisciplinary team, including the colorectal surgeon and the radiation oncologist, to tailor the treatment plan according to the patient's response and preferences, weighing the benefits and risks of each approach.

In summary, my updated favored therapeutic approach for this patient would be to continue with the planned systemic chemotherapy and proceed with definitive radiation therapy as the primary local control strategy, based on the insights from Expert 2 and the goal of preserving the patient's function and quality of life.
