# Supplementary material for: Benchmarking ChatGPT-4 on a radiation oncology in-training exam and Red Journal Gray Zone cases: potentials and challenges for ai-assisted medical education and decision making in radiation oncology
Source: Front Oncol. 2023 Sep 14;13:1265024. doi: 10.3389/fonc.2023.1265024 (PMC10543650; doi:10.3389/fonc.2023.1265024)
Supplement: Supplementary file 2 [file DataSheet_2.zip › Red journal gray zone evaluation/Case1_with_GPT_response.pdf]

GRAY ZONE

CME ASTRO

INTERNATIONAL JOURNAL OF
RADIATION ONCOLOGY • BIOLOGY • PHYSICS

www.redjournal.org

# Sowing the Seeds: A Case of Oligometastatic Anal Cancer 12 Years After Prostate Brachytherapy

Leila T. Tchelebi, MD

*Department of Radiation Medicine, Donald and Barbara Zucker School of Medicine at Hofstra/Northwell, Northwell Health, Lake Success, New York*

Received Aug 8, 2022; Accepted for publication Aug 8, 2022

A 78-year-old man presented with rectal bleeding. Colonoscopy revealed a semicircumferential necrotic mass extending from the dentate proximally into the rectum, measuring 4 cm in length. Biopsy of the lesion was positive for poorly differentiated squamous cell carcinoma. Positron emission tomography–computed tomography revealed the anorectal mass with intense hypermetabolic activity, a 1.3-cm hypermetabolic left inguinal node, and a hypermetabolic lesion in the sacrum (Fig. 1). Sacral

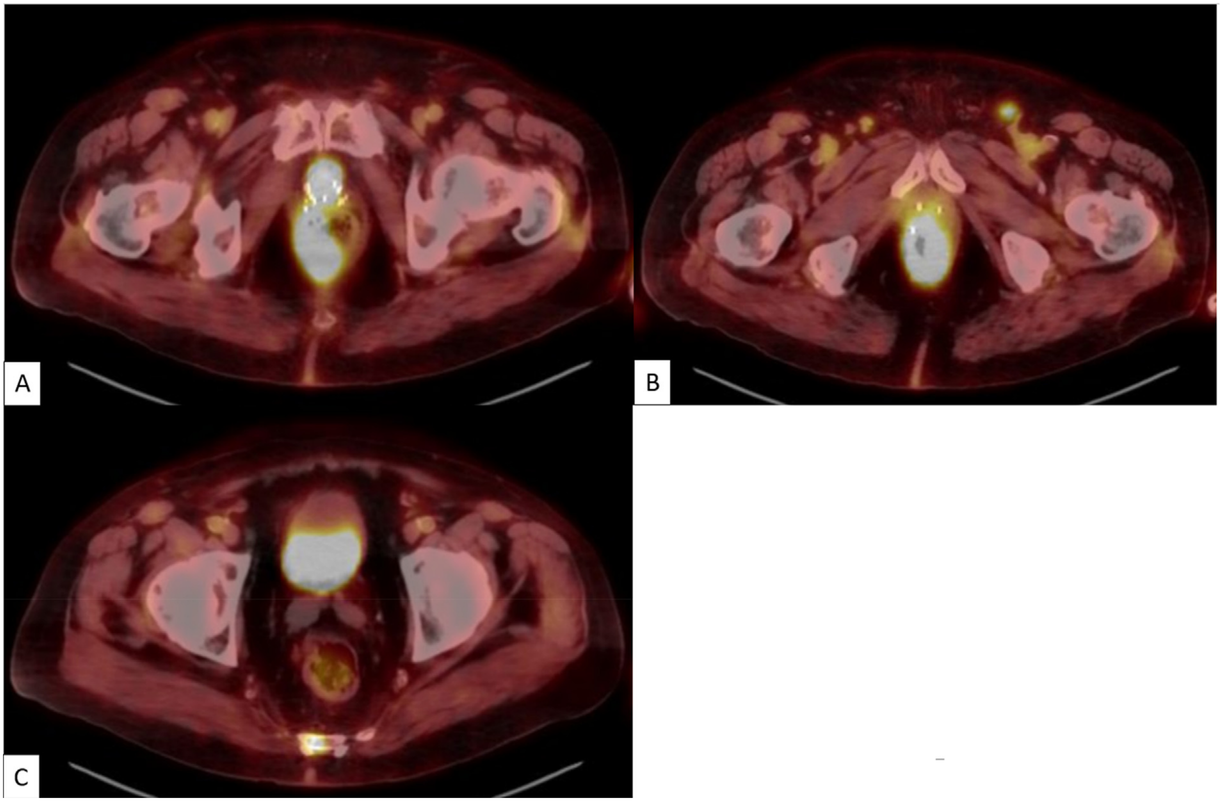


**Fig. 1.** Axial images from the patient's positron emission tomography–computed tomography scan depicting (A) the primary anal canal mass, (B) the left inguinal node metastasis, and (C) the sacral metastasis.

Corresponding author: Leila T. Tchelebi, MD; E-mail: ltchelebi@northwell.edu
Conflict of interest: None.
Disclosures: none.
CME is available for this feature as an ASTRO member benefit, to access visit https://academy.astro.org.

Int J Radiation Oncol Biol Phys, Vol. 114, No. 5, pp. 827–828, 2022
0360-3016/$ - see front matter © 2022 Elsevier Inc. All rights reserved.
https://doi.org/10.1016/j.ijrobp.2022.08.033

biopsy confirmed metastatic, poorly differentiated squamous cell cancer. The patient has a history of low-risk prostate cancer treated with low-dose-rate brachytherapy seed implant monotherapy (total dose, 144 Gy) in 2009.

## Questions

1. What would be your approach to this patient? Would you offer this patient systemic therapy?
2. If you would offer this patient radiation, what would your dose, fractionation, and target volumes be?
3. Would you treat the sacral metastasis first, concurrently, or after treating the primary tumor? What would be your dose and fractionation?
4. Does the previous seed implant affect your management?
5. Do you think the previous brachytherapy treatment resulted in the patient's subsequent development of anal cancer?

INTERNATIONAL JOURNAL OF
RADIATION ONCOLOGY • BIOLOGY • PHYSICS
www.redjournal.org

GRAY ZONE EXPERT OPINIONS

## Systemic Therapy, Then Locoregional Consolidation

We suspect this is a new primary unrelated to prior radiation therapy (RT), a theory which would be supported if the pathologic specimen were found to be associated with human papillomavirus infection.[1] For patients with oligometastatic anorectal squamous cell carcinoma, data suggest more favorable outcomes when treated with systemic therapy plus local therapy targeting the primary and metastatic sites compared with systemic therapy alone.[2]

However, the current scenario is complicated because the patient received prior prostate brachytherapy there-by exposing the anterior rectal wall adjacent to the prostate to radiation and, presumably, a small volume would have received prescription dose. In that context, considerably greater caution is warranted regarding the use of consolidative RT targeting the anorectum because the patient will be at risk of rectal injury, including bleeding, ulceration, perforation, or fistula, which could ultimately be highly morbid.

We favor initial treatment with systemic therapy for 2 to 3 months and assessment of response to determine whether the patient has biologically favorable disease, which supports an aggressive local treatment strategy. If the patient has stable or responsive disease (and specifically lack of further metastatic progression) and favored aggressive therapy after extensive shared decision making, we would treat with RT and concurrent chemotherapy. Positron emission/computed tomography and magnetic resonance imaging would assist with target delineation. Target volumes would include the elective low pelvis and inguinal nodes to 30 Gy, the gross anorectal primary plus 1 cm to 39 Gy, and the involved inguinal lymph node plus 1 cm and involved sacral segment to 45 Gy, using sequential boosts of each 1.5-Gy, twice-daily fractions.[3] If the patient were to be risk averse, we would favor systemic therapy with reservation of RT for palliative-intent purposes.

Roman O. Kowalchuk, MD
Krishan R. Jethwa, MD
*Department of Radiation Oncology*
*Mayo Clinic*
*Rochester, Minnesota*

Int J Radiation Oncol Biol Phys, Vol. 114, No. 5, pp. 829−832, 2022
0360-3016/$ - see front matter © 2022 Published by Elsevier Inc.

Disclosures: K.R.J. is an editor and receives honoraria from RadoncQuestions.com, LLC. R.O.K.'s spouse is employed by GE HealthCare.

## References

1. Tchelebi LT. Sowing the Seeds: A Case of Oligometastatic Anal Cancer 12 Years After Prostate Brachytherapy. *Int J Radiat Oncol Biol Phys* 2022;114:827–828.
2. Jethwa KR, Jin Z, Hallemeier CL. A critical review of the role of local therapy for oligometastatic gastrointestinal cancer [e-pub ahead of print]. *Int J Radiat Oncol Biol Phys.* https://doi.org/10.1016/j.ijrobp.2022.06.084, accessed August 10, 2022.
3. Osborne EM, Eng C, Skibber JM, et al. Hyperfractionated accelerated reirradiation for patients with recurrent anal cancer previously treated with definitive chemoradiation. *Am J Clin Oncol* 2018;41:632–637.

https://doi.org/10.1016/j.ijrobp.2022.08.057

## Warily Whacking the Weeds

Patients with stage IV synchronous oligometastatic anal cancer[1] who are treated with consolidative therapy have a considerable median overall survival (55 vs 17 months) and progression-free survival (16 vs 5 months) advantage compared with patients receiving chemotherapy alone.[2] If local symptoms are manageable, this patient should receive 4 to 6 cycles of systemic therapy (carboplatin/paclitaxel) first. Although there is a risk of toxicity and late fistulation with pelvic irradiation after brachytherapy,[3] symptoms from local progression of anal cancer are detrimental to quality of life due to worsening bleeding and pain. Therefore, since total consolidation can halt symptoms and yield long-term survival, this treatment can be offered after informed patient discussion.

A low dose per fraction should be used to limit late effects, which can include administering 3060 cGy in 17 fractions with intensity modulated radiation therapy to the tumor, anorectum, regional nodes, and involved sacrum with chemotherapy. A sequential boost to 5040 cGy to the gross disease (primary involved nodes, and sacrum) can be

delivered depending on patient tolerance. Prior dosimetry can be fused if feasible, and attention to plan homogeneity at the rectal/prostatic interface with daily image guided radiation therapy is necessary. Although proton therapy may be considered given prior radiation, this treatment is unlikely to benefit since adequate coverage requires anterior rectal wall irradiation.

More than 90% of cases of anal cancer result from human papillomavirus infection. Although the status in unknown, there is no known association between anal squamous cell and brachytherapy. If human papillomavirus infection is negative and there is no immunosuppression history, a secondary neoplasm could be a possible result of prior brachytherapy.

Neil B. Newman, MD, MS
*Department of Radiation Oncology*
*University of Texas Health Science Center at San Antonio*
*San Antonio, Texas*

Jordan Kharofa, MD
*Department of Radiation Oncology*
*University of Cincinnati*
*Cincinnati, Ohio*

Disclosures: None.

## References

1. Tchelebi LT. Sowing the Seeds: A Case of Oligometastatic Anal Cancer 12 Years After Prostate Brachytherapy. *Int J Radiat Oncol Biol Phys* 2022;114:827–828.
2. Eng C, Chang GJ, You YN, et al. The role of systemic chemotherapy and multidisciplinary management in improving the overall survival of patients with metastatic squamous cell carcinoma of the anal canal. *Oncotarget* 2014;5:11133–11142.
3. Hilal L, Wu AJ, Reyngold M, et al. Radiation for anorectal cancers in patients with a history of prostate radiation therapy. *Int J Radiat Oncol Biol Phys* 2021;111:e42–e43.

https://doi.org/10.1016/j.ijrobp.2022.08.059

## Prostrate After Prostate?

For patients with metastatic anal cancer,[1] we recommend starting with carboplatin/paclitaxel, up to 8 cycles, per the InterAACT trial.[2] After chemotherapy, we would restage with positron emission tomography–computed tomography, and if the patient is responding to treatment, offer consolidation chemoradiation to the pelvis, including the sacral metastasis.

We would treat the pelvis, including elective lymph nodes (inguinal, external and internal iliac, presacral, and obturator), to 45 Gy, the fluorodeoxyglucose avid inguinal node to 50.4 Gy, and the primary tumor and sacral metastasis to 54 Gy in 30 fractions via a simultaneous integrated boost. With this plan, most of the previously irradiated anterior rectal wall would receive a total dose of 45 Gy in the elective volume, which we anticipate is a safe cumulative dose given the 13-year interval and that patients on the ASCENDE-RT trial received 46 Gy of external beam pelvic radiation therapy immediately followed by 115 Gy low dose rate brachytherapy.[3] We would contour the urethra and avoid urethral hot spots to minimize risk of urethral-rectal fistula. We would favor treatment on a magnetic resonance imaging–guided linear accelerator for better organ-at-risk protection, including urethra visualization allowing for planning target volume margin reduction to 3 mm. We would plan for weekly adaptation, with additional adaptation on demand. We anticipate the primary tumor would shrink and move further away from the previously irradiated rectum; thus, replanning would allow further cooling of the area of reirradiation overlap. As for cancer etiology, it is certainly possible that the patient's prior low dose rate brachytherapy contributed to his risk for anal squamous cell carcinoma, although dose fall-off from brachytherapy is sharp (>50% at 1 cm) and the patient fits the age demographic for non–HIV-associated anal squamous cell carcinoma.

Maureen L. Aliru, MD, PhD
Nina N. Sanford, MD
*Department of Radiation Oncology*
*University of Texas Southwestern*
*Dallas, Texas*

Disclosures: none.

## References

1. Tchelebi LT. Sowing the Seeds: A Case of Oligometastatic Anal Cancer 12 Years After Prostate Brachytherapy. *Int J Radiat Oncol Biol Phys* 2022;114:827–828.
2. Rao S, Sclafani F, Eng C, et al. International Rare Cancers Initiative multicenter randomized phase II trial of cisplatin and fluorouracil versus carboplatin and paclitaxel in advanced anal cancer: InterAAct. *J Clin Oncol* 2020;38:2510–2518.
3. Rodda S, Tyldesley S, Morris WJ, et al. ASCENDE-RT: An analysis of treatment-related morbidity for a randomized trial comparing a low-dose-rate brachytherapy boost with a dose-escalated external beam boost for high- and intermediate-risk prostate cancer. *Int J Radiat Oncol Biol Phys* 2017;98:286–295.

https://doi.org/10.1016/j.ijrobp.2022.08.038

## BID It to Win It

For this oligometastatic anal squamous cell carcinoma,[1] we would discuss definitive chemoradiation given the paucity of data supporting chemotherapy alone. The safety of pelvic reirradiation has been evaluated in a few prospective trials.[2,3] Up-front surgical resection is a consideration but is less appropriate in the setting of metastatic anal cancer and inguinal node adenopathy. The patient's desire for sphincter preservation should also be evaluated.

We would offer reirradiation with photons or proton therapy. The preferred regimen would be a total dose of 45 Gy in 1.5 Gy twice-daily fractions, followed by boost of 540 cGy in daily 1.8 Gy fractions to the sacral lesion and involved inguinal node. We would reduce the anterior extent of lower dose volume to include only a small portion of the prostate. To minimize the urethral dose, a foley catheter would be used at simulation, and the urethra would be contoured and identified as an organ at risk. The sacral metastasis can be treated concurrently and included in our higher dose target volumes, because the presacral space will be covered regardless and the additional toxicity is minimal.

The prior prostate brachytherapy primarily impacted the anterior rectal wall and prostatic urethra; as such, we would use bid fractionation and limit the total dose to the anal tumor. We would discuss the potential late effects, including proctitis and rectoprostatic fistula. Radiation-induced secondary malignancy is a possibility; however, further assessment of known risk factors for anal cancer, including sexual history and HIV and human papillomavirus status, would help determine the etiology of this anal cancer.

Brianna M. Jones, MD
Karyn A. Goodman, MD
*Department of Radiation Oncology*
*Icahn School of Medicine at Mount Sinai*
*New York, New York*

Disclosures: none.

## References

1. Tchelebi LT. Sowing the Seeds: A Case of Oligometastatic Anal Cancer 12 Years After Prostate Brachytherapy. *Int J Radiat Oncol Biol Phys* 2022;114:827-828.
2. Osborne EM, Eng C, Skibber JM, et al. Hyperfractionated accelerated reirradiation for patients with recurrent anal cancer previously treated with definitive chemoradiation. *Am J Clin Oncol* 2018;41:632-637.
3. Moningi S, Ludmir EB, Polamraju P, et al. Definitive hyperfractionated, accelerated proton reirradiation for patients with pelvic malignancies. *Clin Transl Radiat Oncol* 2019;19:59-65.

https://doi.org/10.1016/j.ijrobp.2022.09.014

## New Growth After Planted Seeds, Treat With Rays

Before Dr. Nigro's excellent 1974 study changed the way that we treat anal canal squamous cell carcinoma, abdominopelvic resections were common but resulted in permanent colostomy for all patients.

So, in reality, the questions we are being asked here are the following:[1]

1. Does the oligometastasis rule out the option of curative treatment?
2. Does the previous brachytherapy make this patient ineligible for an attempt at organ preservation?

Patients on the SABR-COMET trial (including those with colorectal cancer) had a 100% rate of local control when bone metastases were treated with hypofractionated radiation therapy.[2] We would offer this patient curative treatment.

Increased risk of toxic effects with reirradiation leads to increased provider hesitancy to deliver such treatment. However, if intolerable rectal or anal-sphincter toxic effects should result from the reirradiation, this patient would undergo a surgery no worse than the primary surgery that would be planned. He loses nothing, rectum-wise, by trying radiation therapy.

We recommend simultaneous intensity modulated radiation therapy, per anal canal squamous cell treatment guidelines, and stereotactic body radiation therapy to his sacral metastasis, enabling careful control of the total dose to all genitourinary structures, incorporating our best knowledge of his previous brachytherapy dose. We would recommend that chemotherapy start after stereotactic body radiation therapy.

The Surveillance, Epidemiology and End Results Program study by Baxter et al tells us that the risk of rectal cancer increases after prostate radiation therapy, with a hazard ratio of 1.7, but only in irradiated sites.[3] In this case, at least three-fourths of the tumor lies well outside the region exposed to any meaningful dose from prostate brachytherapy; thus, we consider a connection unlikely.

Kathryn E. Hitchcock, MD, PhD
Nicolette R. Drescher, MD
*Department of Radiation Oncology*
*College of Medicine*
*University of Florida*
*Gainesville, Florida*

Disclosures: none.

## References

1. Tchelebi LT. Sowing the Seeds: A Case of Oligometastatic Anal Cancer 12 Years After Prostate Brachytherapy. *Int J Radiat Oncol Biol Phys* 2022;114:827-828.
2. Palma DA, Olson R, Harrow S, et al. Stereotactic ablative radiotherapy for the comprehensive treatment of oligometastatic cancers: Long-term results of the SABR-COMET phase II randomized trial. *J Clin Oncol* 2020;38:2830-2838.
3. Baxter NN, Tepper JE, Durham SB, et al. Increased risk of rectal cancer after prostate radiation: A population-based study. *Gastroenterology* 2005;128:819-824.

https://doi.org/10.1016/j.ijrobp.2022.09.015

**ChatGPT-4: Multimodal Approach Involving Chemotherapy, Radiotherapy, and Possibly Immunotherapy**

***User:*** *You are an expert radiation oncologist from an academic center.*

*Please give your recommendation on the following case:*

*"…"*

*Please give a specific recommendation of your most favored therapeutic approach in this patient case based on the available information and your reasoning for it.*

**ChatGPT-4:** As an expert radiation oncologist, my recommendation for this case would be a multimodal approach including chemotherapy, radiation therapy, and possibly immunotherapy. This is due to the aggressive nature of the poorly differentiated squamous cell carcinoma, the presence of metastatic disease in the inguinal lymph node and sacrum, and the patient's age and history of prior radiation treatment.

1. Chemotherapy: The patient should receive systemic chemotherapy to target the metastatic disease. A commonly used regimen for squamous cell carcinoma of the anal canal is a combination of 5-fluorouracil (5-FU) and cisplatin or carboplatin. The chemotherapy will help to control the disease systemically and also potentially increase the effectiveness of radiation therapy.

2. Radiation therapy: Concurrent chemoradiation is the standard approach for non-metastatic anal cancer. In this case, intensity-modulated radiation therapy (IMRT) would be preferred due to its ability to deliver precise radiation doses, sparing nearby healthy tissue, and potentially reducing side effects. The radiation oncologist will need to carefully consider the prior radiation exposure from the patient's brachytherapy for prostate cancer to avoid exceeding dose constraints to the surrounding organs at risk. The target volumes should include the primary tumor, the involved inguinal lymph node, and the sacral metastasis.

3. Immunotherapy: Given the poorly differentiated nature of the tumor, the patient might benefit from immunotherapy, specifically PD-1 or PD-L1 inhibitors such as pembrolizumab or nivolumab. However, it is important to determine the patient's tumor PD-L1 status and discuss potential side effects before deciding on this approach.

The patient should also be evaluated by a multidisciplinary team, including medical oncologists, radiation oncologists, and surgical oncologists. Regular follow-ups and imaging should be performed to assess treatment response and manage potential side effects.

It is important to note that every patient's case is unique, and the final treatment plan should be individualized based on the patient's overall health, preferences, and potential side effects of the therapies. In some cases, palliative care may also be considered to manage symptoms and improve quality of life.

***User:*** *Would you offer this patient systemic therapy?*

**ChatGPT-4:** Yes, I would recommend systemic therapy for this patient, as the presence of metastatic disease in the inguinal lymph node and the sacrum suggests the need for systemic treatment in addition to local therapy. Systemic therapy, such as chemotherapy, can help control the spread of the cancer and improve the patient's prognosis.

A commonly used chemotherapy regimen for squamous cell carcinoma of the anal canal is a combination of 5-fluorouracil (5-FU) and cisplatin or carboplatin. This combination has demonstrated efficacy in treating anal cancer and is typically given concurrently with radiation therapy.

Additionally, given the poorly differentiated nature of the tumor, it may be worth considering immunotherapy, specifically PD-1 or PD-L1 inhibitors such as pembrolizumab or nivolumab. However, the patient's tumor PD-L1 status should be determined, and potential side effects should be discussed before deciding on this approach.

It is important to note that the choice of systemic therapy should be individualized based on the patient's overall health, comorbidities, and preferences. A multidisciplinary team of medical oncologists, radiation oncologists, and surgical oncologists should be involved in evaluating the patient and formulating a comprehensive treatment plan.

***User:** If you would offer this patient radiation, what would your dose, fractionation, and target volumes be?*

**ChatGPT-4:** In this specific case, considering the metastatic nature of the disease, I would recommend a chemoradiation approach with the following radiation dose, fractionation, and target volumes:

Dose and fractionation: A standard dose-fractionation scheme for anal cancer typically involves delivering 45 to 50.4 Gy to the primary tumor and involved lymph nodes, using daily fractions of 1.8 to 2 Gy, given over 5-6 weeks. However, due to the patient's prior radiation therapy for prostate cancer, it is important to carefully consider the cumulative dose to surrounding organs at risk. The radiation oncologist may need to adjust the dose or fractionation to account for the patient's individual situation and to minimize the risk of late radiation-induced toxicity.

Target volumes:

a) Gross Tumor Volume (GTV): The primary anal canal mass, the left inguinal node metastasis, and the sacral metastasis should be included in the GTV. These volumes are based on the findings from the positron emission tomography-computed tomography (PET-CT) scan.

b) Clinical Target Volume (CTV): The CTV should include the GTV with an appropriate margin to cover any microscopic disease spread. For the primary tumor, the CTV should encompass the entire anal canal and mesorectal fat. The CTV for the involved inguinal node should include the entire left inguinal nodal basin, while the CTV for the sacral metastasis should encompass the involved bone and surrounding soft tissue with an adequate margin.

c) Planning Target Volume (PTV): The PTV accounts for uncertainties in patient positioning and internal organ motion. An appropriate margin should be added around the CTV to create the PTV.

Intensity-modulated radiation therapy (IMRT) would be the preferred radiation technique for this case, as it allows for better dose conformity to the target volumes while minimizing the dose to the surrounding healthy tissues. Furthermore, image-guided radiation therapy (IGRT) should be utilized for accurate patient positioning and target localization throughout the course of treatment.

Please note that these recommendations are based on a general understanding of the patient's condition and should be tailored to the individual patient's needs and circumstances. It is important to discuss the treatment plan with a multidisciplinary team of specialists, taking into account the patient's overall health, prior radiation exposure, and potential side effects.

***User:*** *Would you treat the sacral metastasis first, concurrently, or after treating the primary tumor? What would be your dose and fractionation?*

**ChatGPT-4:** In this case, the decision to treat the sacral metastasis first, concurrently, or after treating the primary tumor would depend on the patient's symptoms, performance status, and overall treatment goals. However, a common approach would be to treat the primary tumor and sacral metastasis concurrently, given the presence of metastatic disease and the need for systemic therapy.

For the sacral metastasis, palliative radiation therapy can be utilized to control the tumor, alleviate pain, and potentially prevent complications such as pathological fractures. A typical palliative regimen might include:

Hypofractionated radiation therapy: A total dose of 20-30 Gy in 5-10 fractions, with each fraction delivered over 1-2 weeks. This regimen is effective in providing pain relief and local control with minimal side effects.

Alternatively, a more protracted course of radiation therapy could be considered if the patient's performance status and overall treatment goals support a more aggressive approach. In this case, the total dose might be increased to 30-40 Gy in 10-20 fractions, delivered over 2-4 weeks.

It is important to note that the dose and fractionation for the sacral metastasis would be separate from the concurrent chemoradiotherapy (CRT) delivered to the primary anorectal tumor and involved lymph nodes. The radiation oncologist should carefully plan the treatment to minimize overlap between the target volumes and to avoid exceeding the tolerance doses for critical organs such as the bowel, bladder, and spinal cord.

The chosen dose and fractionation for the sacral metastasis should be individualized based on the patient's symptoms, tolerance to treatment, and response to therapy. Collaboration with the medical oncologist and other members of the multidisciplinary team is essential to ensure optimal patient management.

*User: Does the previous seed implant affect your management?*

**ChatGPT-4:** The previous low-dose-rate brachytherapy seed implant for prostate cancer (total dose, 144 Gy) in 2009 does affect the management of this patient to some extent. It is important to consider the potential risks and complications associated with re-irradiation, as the patient has already received a significant dose of radiation to the pelvic region, which may increase the likelihood of late radiation-induced toxicity.

When planning the radiation treatment for the anorectal tumor and involved lymph nodes, the radiation oncologist should take into account the prior radiation exposure to the bladder, bowel, and surrounding normal tissues. Modern radiation techniques, such as intensity-modulated radiation therapy (IMRT) or volumetric-modulated arc therapy (VMAT), can be used to minimize the dose to these critical structures, reducing the risk of treatment-related side effects.

The dose and fractionation for the primary tumor and involved lymph nodes may need to be adjusted based on the patient's tolerance to treatment and potential side effects. It is crucial to closely monitor the patient throughout the treatment process for signs of acute or late toxicity and to manage any complications that may arise.

Furthermore, the presence of the previous seed implant highlights the importance of a multidisciplinary approach to the patient's care. The radiation oncologist should collaborate with the medical oncologist, surgical oncologist, and other members of the treatment team to ensure that the patient's management plan is well-coordinated and balances the need for local control, systemic control, and minimizing treatment-related complications.

*User: Do you think the previous brachytherapy treatment resulted in the patient's subsequent development of anal cancer?*

**ChatGPT-4:** It is unlikely that the previous brachytherapy treatment for prostate cancer directly resulted in the patient's subsequent development of anal cancer. While radiation therapy can increase the risk of developing secondary malignancies, the risk is generally low and typically arises after a long latency period (usually more than 10 years).

In the case of brachytherapy for prostate cancer, the radiation dose is delivered via implanted radioactive seeds directly into the prostate gland, resulting in a highly localized radiation distribution. This means that the surrounding tissues receive relatively low doses of radiation compared to external beam radiation therapy. The anorectal region is not in the direct vicinity of the prostate, making it even less likely that the previous brachytherapy treatment led to the development of anal cancer.

It is important to note that anal cancer is most commonly associated with other risk factors, such as infection with human papillomavirus (HPV), smoking, and immunosuppression. These factors are more likely to have contributed to the development of anal cancer in this patient than the prior brachytherapy treatment for prostate cancer.

However, it is still essential to carefully consider the potential risks and complications associated with additional radiation therapy in a patient with a history of prior radiation treatment, as mentioned in previous responses.

***User:** There are five other experts' recommendations. (Other experts' recommendations are provided.) Please summarize the three experts' recommendations in short sentences.*

**ChatGPT-4:** Expert 1: Recommends initial systemic therapy, followed by assessment of response, and then consideration of chemoradiation with cautious dose-fractionation for locoregional consolidation if the disease is stable or responsive.

Expert 2: Suggests starting with 4-6 cycles of systemic therapy (carboplatin/paclitaxel), then considering consolidation treatment with a low dose per fraction of radiation therapy to the tumor, anorectum, regional nodes, and involved sacrum, depending on patient tolerance.

Expert 3: Advises starting with up to 8 cycles of carboplatin/paclitaxel chemotherapy, followed by restaging and consolidation chemoradiation to the pelvis, including the sacral metastasis, if the patient responds to treatment.

Expert 4: Proposes definitive chemoradiation with either photons or proton therapy, using a twice-daily fractionation schedule and limiting the total dose to the anal tumor, while discussing potential late effects with the patient.

Expert 5: Recommends simultaneous intensity-modulated radiation therapy and stereotactic body radiation therapy to the sacral metastasis, with careful control of the total dose to genitourinary structures, followed by chemotherapy.

***User:** As an expert radiation oncologist, which of the above five experts' recommendation do you think the most proper for the patient described above?*

**ChatGPT-4:** As an expert radiation oncologist, I find Expert 3's recommendation to be the most proper approach for the patient described above. This approach involves starting with up to 8 cycles of carboplatin/paclitaxel chemotherapy, followed by restaging and consolidation chemoradiation to the pelvis, including the sacral metastasis if the patient responds to treatment.

This recommendation accounts for the metastatic nature of the disease and the need for systemic treatment while also considering the benefits of local consolidation therapy if the disease is responsive to chemotherapy. Additionally, the suggested chemoradiation plan carefully addresses the patient's prior radiation exposure and aims to minimize the risk of late radiation-induced toxicity.

***User:** At the beginning, you said that "my recommendation for this case would be a multimodal approach including chemotherapy, radiation therapy, and possibly immunotherapy." In this case, which expert's recommendation is your initial suggestion closest to?*

**ChatGPT-4:** My initial suggestion of a multimodal approach involving chemotherapy, radiation therapy, and possibly immunotherapy is closest to Expert 3's recommendation. Expert 3 suggests starting with up to 8 cycles of carboplatin/paclitaxel chemotherapy, followed by restaging and consolidation chemoradiation to the pelvis, including the sacral metastasis if the patient responds to treatment.

Although Expert 3 does not explicitly mention immunotherapy, their recommendation combines chemotherapy and radiation therapy in a manner that addresses both the systemic and locoregional aspects of the disease. This approach considers the patient's prior radiation exposure and aims to minimize the risk of late radiation-induced toxicity. Immunotherapy can be further discussed with the patient and the treatment team, depending on the patient's response to chemotherapy and other factors.

***User:** After seeing the other five experts' recommendations, are you going to update your own recommendation of your most favored therapeutic approach in this patient case? If so, what is your updated recommendation and which expert/experts is your update based on?*

**ChatGPT-4:** After reviewing the other five experts' recommendations, I would update my initial recommendation. My updated recommendation would be to follow Expert 3's approach, which I find to be the most balanced and comprehensive for the patient described.

My updated recommendation involves:

- Starting with up to 8 cycles of carboplatin/paclitaxel chemotherapy.
- Restaging the patient after chemotherapy and evaluating their response to treatment.
- If the patient responds well, proceeding with consolidation chemoradiation to the pelvis, including the sacral metastasis. This would involve treating the pelvis, elective lymph nodes, and the primary tumor and sacral metastasis with a simultaneous integrated boost.

Expert 3's recommendation takes into account the metastatic nature of the disease, the need for systemic treatment, and the potential benefits of local consolidation therapy. It also addresses the patient's prior radiation exposure and aims to minimize the risk of late radiation-induced toxicity. Immunotherapy can still be considered in the treatment plan and discussed with the patient and the treatment team depending on the patient's response to chemotherapy and other factors.
