# Supplementary material for: Benchmarking ChatGPT-4 on a radiation oncology in-training exam and Red Journal Gray Zone cases: potentials and challenges for ai-assisted medical education and decision making in radiation oncology
Source: Front Oncol. 2023 Sep 14;13:1265024. doi: 10.3389/fonc.2023.1265024 (PMC10543650; doi:10.3389/fonc.2023.1265024)
Supplement: Supplementary file 2 [file DataSheet_2.zip › Red journal gray zone evaluation/Case3_with_GPT_response.pdf]

GRAY ZONE

CME ASTRO

INTERNATIONAL JOURNAL OF
RADIATION ONCOLOGY • BIOLOGY • PHYSICS

www.redjournal.org

Adjuvant Head and Neck Radiation in a Young Pregnant Woman

Anand K. Sharma, MBBS

HCA South Atlantic Division, H&N Radiation Oncology, Trident Medical Center, Charleston, South Carolina

Received May 5, 2022; Accepted for publication Jul 18, 2022

A 32-year-old, pregnant (14 weeks), female patient presented to the emergency room with a 3-month history of a nonhealing, painful ulcer on the right side of the tongue that she initially thought was an accidental bite. She was prescribed antibiotics with recommendations to see oral surgery if no improvement. The patient consulted with oral surgery, and underwent biopsy testing of a 3.5 cm right lateral tongue ulcer. The biopsy test results were consistent with moderately differentiated keratinizing squamous cell carcinoma (p16 negative). A computed tomography scan of the neck revealed a 3.5 $\times$ 1.8 cm enhancing lesion on the right lateral tongue, extending through the full thickness of the tongue without suspicious adenopathy. The patient has a history of smoking (1/4 pack per day for 10 years), but quit 3 years prior.

She underwent a hemiglossectomy and bilateral neck dissection with free-flap reconstruction. Surgical pathology tests revealed 3.5 cm poorly differentiated squamous cell carcinoma with a 13-mm depth of invasion and negative surgical margins (closest margin: 4 mm). Perineural invasion was present without lymphovascular invasion. One ipsilateral, level 2A lymph node (4 mm) was positive without ECE. The patient's carcinoma was staged as pT3, pN1, Mo. She did well after surgery, and was discharged on postoperative day 7. She was 17 weeks pregnant on the discharge date.

Questions

1. Would you offer adjuvant radiation therapy?
2. If offering radiation therapy, what modality would you employ? External beam radiation therapy (intensity modulated radiation therapy vs 3-dimensional)? Brachytherapy? Any special timing or technical considerations for a pregnant patient?
3. If offering radiation therapy, would you include the right neck in the target volume?
4. Is there a role for systemic therapy?
5. What counseling should be provided to this patient given her second trimester status?

Corresponding author: Anand K. Sharma, MBBS.; E-mail: anand.sharma@hcahealthcare.com
Conflict of interest: None.
Disclosures: None.
CME is available for this feature as an ASTRO member benefit, to access visit https://academy.astro.org.

Int J Radiation Oncol Biol Phys, Vol. 114, No. 3, p. 382, 2022
0360-3016/$ - see front matter © 2022 Elsevier Inc. All rights reserved.
https://doi.org/10.1016/j.ijrobp.2022.07.031

INTERNATIONAL JOURNAL OF
RADIATION ONCOLOGY • BIOLOGY • PHYSICS
www.redjournal.org

GRAY ZONE EXPERT OPINIONS

One Treatment But Two Patients

1. We recommend adjuvant radiation therapy (RT) to the primary site and both sides of the neck.[1] The patient had numerous adverse features that correlate with a significant risk of recurrence and successful salvage of recurrence is unlikely.
2. Intensity-modulated radiation therapy is possible despite increasing the fetal dose compared with 3-dimensional conventional radiation therapy. Fetal shielding, keeping the modulation low, rotation of the collimator (so the multileaf collimator opens along the patient axis), and consideration of flattening-filter-free mode can help mitigate the fetal risk.[2] Final decisions of external beam radiation therapy technique would be made after fetal dose estimations.

Initiating RT 4 weeks after surgery would start treatment at 21 weeks. This is acceptable as the patient is past the most radiosensitive stages of pregnancy.[3] Delaying counters the preferential timing of postoperative RT, and the presumed enhanced safety later in pregnancy is offset by the growth of the fetus closer to the delivered radiation field.

3. Omitting the right neck would not offer meaningful fetal protection.
4. The pregnancy is not a specific contraindication to chemotherapy. The complexity is more that the case described numerous adverse features, but not a positive margin or extranodal extension for which there is consensus for concurrent chemo-RT. There are no data to support aggressive adjuvant chemotherapy in lieu of RT.
5. The patient's personal priorities may affect the treatment strategy. Although the risk is low to the fetus, there is a remote possibility of effect on growth or cognition and malformation. Additionally, the morbidity of the treatment on the patient may be more difficult to manage during a pregnancy and subsequently with a newborn.

Adam S. Garden, MD
Departments of Radiation Oncology
UT MD Anderson Cancer Center
Houston, Texas

Stephen F. Kry, PhD
Radiation Physics
UT MD Anderson Cancer Center
Houston, Texas

Disclosures: none.

References

1. Sharma AK. Head and neck radiation in a young pregnant woman. *Int J Radiat Oncol Biol Phys* 2022;114:382.
2. Kry SF, Bednarz B, Howell RM, et al. AAPM TG-158: Measurement and calculation of doses outside the treated volume from external-beam radiation therapy. *Med Phys* 2017;44:391–429.
3. Stovall M, Blackwell CR, Cundiff J, et al. Fetal dose from radiotherapy with photon beams: Report of AAPM Radiation Therapy Committee task group No. 36. *Med Phys* 1995;22:63–82.

https://doi.org/10.1016/j.ijrobp.2022.07.1829

What to Expect When You're Expecting: Managing Oral Cavity Cancer in the Setting of a Second Trimester Pregnancy

It would be standard to recommend postoperative radiation to this young pregnant female with resected locally advanced oral cavity cancer and multiple risk factors

Int J Radiation Oncol Biol Phys, Vol. 114, No. 3, pp. 383−384, 2022
0360-3016/$ - see front matter © 2022 Elsevier Inc. All rights reserved.

including pT3 primary, depth of invasion ≥4 mm, close surgical margins (≤5 mm), perineural invasion, and positive lymph node.[1] The complicating factor is that she is 17 weeks pregnant. Typically, radiation therapy during pregnancy is avoided due to fetal health concerns including miscarriage, fetal death, and radiation-induced malignancies. Given the distance between the target site and pelvis, the primary concern is the peripheral radiation dose, which is comprised of external and internal scatter to the fetus. Radiation during the middle trimester has previously been associated with intellectual delay.[2] Multiple studies have demonstrated peripheral fetal doses of <0.1 Gy with prescription doses of up to 66 Gy, using lead shielding of the pelvis to minimize the dose.[3]

Despite a relatively low fetal radiation dose, we would have a thorough discussion with the patient regarding the risks and benefits of adjuvant photon radiation to 60 Gy to the bilateral neck with lead shielding. We recommend the patient seek a radiation oncologist with experience in treating patients during pregnancy. Overall, we favor 3-dimensional conformal radiotherapy over intensity-modulated radiation therapy due to increased internal scatter associated with the use of collimators in the latter, which would increase radiation dose to the fetus. We would obtain diagnostic noncontrast magnetic resonance imaging for treatment planning and recommend establishing care with a high-risk obstetrician/gynecologist. We would not recommend systemic therapy given health concerns for the fetus and the absence of positive margins or extranodal extension.

Jennifer Ma, MD
Nancy Y. Lee, MD, FASTRO
Department of Radiation Oncology
Memorial Sloan Kettering Cancer Center
New York, New York

Disclosures: N.Y.L. serves on advisory boards for Merck, Merck Serono, Pfizer, Elsie, and Mirati. The other author has no disclosures to declare.

References

1. Sharma AK. Head and neck radiation in a young pregnant woman. *Int J Radiat Oncol Biol Phys* 2022;114:382.
2. Kal HB, Struikmans H. Radiotherapy during pregnancy: Fact and fiction. *Lancet Oncol* 2005;6:328–333.
3. Podgorsak MB, Meiler RJ, Kowal H, Kishel SP, Orner JB. Technical management of a pregnant patient undergoing radiation therapy to the head and neck. *Med Dosim* 1999;24:121–128.

https://doi.org/10.1016/j.ijrobp.2022.07.1828

Just Because You Could, Does Not Mean You Should

Radiation therapy (RT) should be avoided in pregnancy except for select cases where the benefit clearly outweighs the risks. Here, T3 (American Joint Committee on Cancer 8), close margins, and perineural invasion (PNI) are risk factors for a locoregional recurrence (LRR).[1] For similar cases, we would routinely recommend RT to reduce LRR risk. We would treat the primary site and bilateral neck with IMRT to 60 Gy in 30 fractions starting within 6 weeks of surgery. Given no extracapsular extension or positive margins and only a single lymph node metastasis, we would favor no chemotherapy.

From a technical standpoint, fetal dose below the deterministic threshold of 0.1 Gy is possible even without modern techniques. A similar case of a 29-year-old woman with oral tongue squamous cell carcinoma who was 16 weeks pregnant at surgery was reported.[2] She was treated with opposed laterals and electron boost starting 6 weeks after surgery and a healthy baby was delivered 7 weeks after treatment. We would counsel the patient that second trimester pregnancy risks including mental retardation, microcephaly, and carcinogenesis are uncertain, likely small, but not zero. Conversely, assessing the magnitude of benefit of RT is modulated by missing information, namely the number of dissected nodes and the extent of PNI. If the neck dissection was adequate (>18 nodes) and PNI was only a small, single focus, then the LRR risk without RT may be small.[3] Ultimately, we would offer adjuvant RT during pregnancy if desired by the patient.

Sumi Sinha, MD
Jason W. Chan, MD
Department of Radiation Oncology
University of California San Francisco,
San Francisco, California

Disclosures: none.

References

1. Sharma AK. Head and neck radiation in a young pregnant woman. *Int J Radiat Oncol Biol Phys* 2022;114:382.
2. Nuyttens JJ, Prado KL, Jenrette JM, Williams TE. Fetal dose during radiotherapy: Clinical implementation and review of the literature. *Cancer Radiother* 2002;6:352–357.
3. Koyfman SA, Ismaila N, Crook D, et al. Management of the neck in squamous cell carcinoma of the oral cavity and oropharynx: ASCO clinical practice guideline. *J Clin Oncol* 2019;37:1753–1774.

https://doi.org/10.1016/j.ijrobp.2022.07.1830

See redjournal.org for an additional opinion ~~to~~ this case, “Reconsidering How it Begins.”

ChatGPT-4: Maximal Cancer Control, Minimal Risk to Fetus

User: *You are an expert radiation oncologist from an academic center.*

Please give your recommendation on the following case:

"…"

Please give a specific recommendation of your most favored therapeutic approach in this patient case based on the available information and your reasoning for it.

ChatGPT-4: Given that the patient is 17 weeks pregnant, her management requires a multidisciplinary approach involving the radiation oncologist, medical oncologist, obstetrician, and oral surgeon to ensure the best possible outcome for both the mother and the fetus.

My recommendation would be as follows:

Adjuvant therapy: As the patient has a high-risk tumor (pT3, pN1, and presence of perineural invasion), adjuvant therapy should be considered. The choice of adjuvant therapy (chemotherapy or radiation) would depend on the evaluation of potential risks to the fetus and the mother.

Radiation therapy: Radiation therapy can be a suitable adjuvant treatment option for this patient. However, given the patient's pregnancy, additional precautions should be taken to minimize radiation exposure to the fetus. Intensity-modulated radiation therapy (IMRT) or volumetric modulated arc therapy (VMAT) can be used to target the tumor bed and the ipsilateral neck with higher precision and lower radiation doses to surrounding tissues. The treatment can be planned during the second trimester (when the patient is beyond 17 weeks pregnant) when the fetus is less sensitive to radiation.

Chemotherapy: The use of chemotherapy in pregnant patients is generally avoided, especially during the first trimester due to potential teratogenic effects on the fetus. However, certain chemotherapy agents like cisplatin have been used during the second and third trimesters in some cases with close monitoring. The decision to use chemotherapy as part of adjuvant treatment should be made after thorough discussion with the patient and a careful evaluation of the potential risks and benefits.

Close monitoring: The patient should be closely monitored by the obstetrician during and after the radiation therapy. A fetal ultrasound should be performed before initiating treatment and at regular intervals throughout the therapy to ensure the health of the fetus.

The therapeutic approach recommended above aims to maximize the chances of controlling the patient's cancer while minimizing the risks to the fetus. It is important to have a detailed discussion with the patient about the benefits and potential risks of the proposed treatment plan and obtain her informed consent. Additionally, the patient should be counseled on the importance of regular follow-up and surveillance for early detection of potential recurrences or metastasis.

User: *Would you offer adjuvant radiation therapy?*

ChatGPT-4: As an expert radiation oncologist, I would consider offering adjuvant radiation therapy to this patient due to her high-risk features (pT3, pN1, and presence of perineural invasion). Adjuvant radiation therapy can help reduce the risk of local recurrence and improve overall outcomes.

However, given the patient's pregnancy, additional precautions must be taken to minimize radiation exposure to the fetus. Treatment can be planned during the second trimester (when the patient is beyond 17 weeks pregnant), as the fetus is less sensitive to radiation at this stage. Intensity-modulated radiation therapy (IMRT) or volumetric modulated arc therapy (VMAT) can be employed to target the tumor bed and the ipsilateral neck with higher precision and lower radiation doses to surrounding tissues.

Close collaboration with the obstetrician, medical oncologist, and oral surgeon is essential to ensure the best possible outcome for both the mother and the fetus. The patient should be closely monitored during and after radiation therapy, with fetal ultrasounds performed before initiating treatment and at regular intervals throughout the therapy to ensure the health of the fetus.

It is important to discuss the potential benefits and risks of adjuvant radiation therapy with the patient and obtain her informed consent. The patient should also be counseled on the importance of regular follow-up and surveillance for early detection of potential recurrences or metastasis.

User: *If offering radiation therapy, what modality would you employ? External beam radiation therapy (intensity modulated radiation therapy vs 3-dimensional)? Brachytherapy? Any special timing or technical considerations for a pregnant patient?*

ChatGPT-4: For this patient, I would recommend using external beam radiation therapy (EBRT) with intensity-modulated radiation therapy (IMRT) or volumetric modulated arc therapy (VMAT). These techniques offer better dose conformity and precision in targeting the tumor bed and the ipsilateral neck while minimizing radiation exposure to the surrounding healthy tissues and the fetus. IMRT and VMAT allow for more accurate dose distribution compared to 3-dimensional conformal radiation therapy (3D-CRT).

Brachytherapy is generally not the preferred modality for head and neck cancers, as it is more commonly used for treating gynecologic, prostate, and some skin cancers. Furthermore, brachytherapy might not provide optimal coverage of the tumor bed and the lymph nodes at risk in this particular case.

For a pregnant patient, timing and technical considerations are crucial. Treatment should be planned during the second trimester (beyond 17 weeks pregnant), as the fetus is less sensitive to radiation during this period. It is essential to take additional precautions to minimize radiation exposure to the fetus:

1. Shielding: Using abdominal and pelvic shielding to further protect the fetus from scatter radiation.
2. Positioning: Carefully positioning the patient to minimize the distance between the treatment area and the fetus, reducing the chance of fetal exposure.
3. Dosimetry: Collaborating with a medical physicist to optimize the treatment plan and ensure the lowest possible dose to the fetus while effectively treating the cancer.
4. Monitoring: Performing fetal ultrasounds before initiating treatment and at regular intervals during therapy to ensure the health of the fetus.

A multidisciplinary approach involving the radiation oncologist, medical oncologist, obstetrician, and oral surgeon is necessary to ensure the best possible outcome for both the mother and the fetus. The patient should be thoroughly informed of the potential benefits and risks of radiation therapy and provide her informed consent. Regular follow-up and surveillance are also essential for early detection of potential recurrences or metastasis.

User: *If offering radiation therapy, would you include the right neck in the target volume?*

ChatGPT-4: Yes, I would include the right neck in the target volume for radiation therapy. As the patient's carcinoma is staged as pT3, pN1, and has perineural invasion, there is a higher risk of local recurrence and regional lymph node involvement. Including the right neck in the target volume, specifically focusing on the ipsilateral neck levels at risk, would help reduce the likelihood of regional recurrence.

Using intensity-modulated radiation therapy (IMRT) or volumetric modulated arc therapy (VMAT) would allow for better dose conformity and precision in targeting both the tumor bed and the right neck while minimizing radiation exposure to the surrounding healthy tissues and the fetus. This is particularly important in this case, as the patient is pregnant, and minimizing radiation exposure to the fetus is crucial.

As always, a multidisciplinary approach involving the radiation oncologist, medical oncologist, obstetrician, and oral surgeon is necessary to ensure the best possible outcome for both the mother and the fetus. The patient should be informed of the potential benefits and risks of radiation therapy, and provide her informed consent. Regular follow-up and surveillance are essential for early detection of potential recurrences or metastasis.

User: *Is there a role for systemic therapy?*

ChatGPT-4:

There might be a role for systemic therapy in this patient's case, particularly considering the high-risk features of the tumor (pT3, pN1, and presence of perineural invasion). Systemic therapy, such as chemotherapy, could be employed as an adjuvant treatment to reduce the risk of recurrence and improve overall outcomes.

However, the use of chemotherapy in pregnant patients is generally avoided, especially during the first trimester, due to potential teratogenic effects on the fetus. Certain chemotherapy agents, like cisplatin, have been used during the second and third trimesters in some cases with close monitoring. The decision to use chemotherapy as part of adjuvant treatment should be made after thorough discussion with the patient and a careful evaluation of the potential risks and benefits.

A multidisciplinary approach involving the radiation oncologist, medical oncologist, obstetrician, and oral surgeon is necessary to ensure the best possible outcome for both the mother and the fetus. It is essential to weigh the benefits of systemic therapy against the potential risks to the fetus, and to closely monitor the patient during and after treatment. The patient should be informed of the potential benefits and risks of systemic therapy and provide her informed consent. Regular follow-up and surveillance are also essential for early detection of potential recurrences or metastasis.

***User:** What counseling should be provided to this patient given her second trimester status?*

ChatGPT-4: Counseling for this patient should involve a multidisciplinary approach, with input from the radiation oncologist, medical oncologist, obstetrician, and oral surgeon. Given her second trimester status, several key topics should be addressed during counseling:

Cancer treatment options: Explain the rationale for recommending adjuvant therapy, such as radiation therapy and/or chemotherapy, to reduce the risk of recurrence and improve overall outcomes. Discuss the potential benefits and risks of each treatment option, including their impact on the pregnancy.

Timing of treatment: Emphasize that the second trimester is considered a safer time for cancer treatment, as the fetus is less sensitive to radiation and some chemotherapy agents during this period. However, it is still essential to minimize radiation exposure to the fetus.

Radiation therapy considerations: Discuss the use of advanced techniques like IMRT or VMAT, which allow for better dose conformity and precision in targeting the tumor bed and ipsilateral neck while minimizing radiation exposure to the fetus. Explain the importance of shielding, positioning, and treatment planning in reducing fetal exposure.

Chemotherapy considerations: Explain the risks associated with chemotherapy during pregnancy, particularly during the first trimester. Discuss the potential use of specific chemotherapy agents, like cisplatin, during the second and third trimesters with close monitoring.

Monitoring during treatment: Ensure the patient understands the importance of close collaboration between the obstetrician and oncology team during and after treatment. Explain the role of fetal ultrasounds in monitoring the health of the fetus throughout treatment.

Potential complications: Discuss possible short-term and long-term complications of cancer treatment during pregnancy for both the mother and the fetus. These may include preterm labor, low birth weight, and developmental issues in the child.

Informed consent: Emphasize the importance of the patient's informed consent for the proposed treatment plan, and ensure she has a clear understanding of the potential benefits and risks.

Follow-up and surveillance: Stress the importance of regular follow-up and surveillance for early detection of potential recurrences or metastasis, and the need for ongoing communication between the patient and her healthcare team.

Emotional support: Address the patient's emotional well-being, as cancer diagnosis and treatment during pregnancy can be particularly challenging. Encourage her to seek support from friends, family, support groups, or mental health professionals as needed.

User: *Please summarize the four experts' recommendations in short sentences.*

ChatGPT-4: Expert 1: Recommends adjuvant radiation therapy (RT) to the primary site and both sides of the neck, considering intensity-modulated radiation therapy (IMRT) with fetal dose mitigation techniques, and avoiding systemic chemotherapy due to lack of consensus and potential risks to the fetus. Emphasizes patient's personal priorities in treatment strategy.

Expert 2: Suggests adjuvant photon radiation to bilateral neck with lead shielding while considering fetal health concerns, recommends 3-dimensional conformal radiotherapy over IMRT to minimize fetal exposure, and advises against systemic therapy due to health concerns for the fetus.

Expert 3: Advocates avoiding radiation therapy during pregnancy except in cases where the benefit clearly outweighs the risks. Considers offering adjuvant RT during pregnancy if desired by the patient and based on the assessment of potential benefits.

Expert 4: Emphasizes the importance of multidisciplinary evaluation and considering all options for pregnancy management. Suggests induction chemotherapy during pregnancy, delaying radiation therapy until after the resolution of pregnancy, and discussing adjuvant chemotherapy in the interim to minimize risks to both the mother and the fetus.

User: *As an expert radiation oncologist, which of the above four experts' recommendation do you think the most proper?*

ChatGPT-4: As an expert radiation oncologist, I find Expert 4's recommendation to be the most proper, as it provides a comprehensive approach that takes into account the complex interplay of cancer treatment and pregnancy management. Expert 4 highlights the importance of a multidisciplinary evaluation, involving maternal-fetal medicine specialists, to explore all available options for both cancer management and pregnancy care.

Expert 4's recommendation considers alternative treatment options, such as induction chemotherapy during pregnancy, which may help control the disease and minimize the risks associated with radiation therapy during pregnancy. They also suggest delaying radiation therapy until after the resolution of the pregnancy, whenever possible, and discussing the use of adjuvant chemotherapy in the interim.

While Expert 1's recommendation is also balanced and comprehensive, Expert 4's approach emphasizes the crucial conversations and collaboration between the patient, oncologists, and maternal-fetal medicine providers to determine the best course of action for both the mother and the fetus. This approach ensures that all aspects of the patient's care are considered, resulting in the most appropriate and personalized treatment plan.

User: *As an expert radiation oncologist, you have suggested a multidisciplinary approach involving the radiation oncologist, medical oncologist, obstetrician, and oral surgeon to ensure the best possible outcome for both the mother and the fetus. It includes adjuvant therapy, radiation therapy, chemotherapy, and close monitoring. In this case, which expert's recommendation is your suggestion closest to?*

ChatGPT-4: My suggestion is closest to Expert 1's recommendation. Expert 1 provides a balanced and comprehensive approach that considers adjuvant radiation therapy to the primary site and both sides of the neck due to the multiple adverse features and significant risk of recurrence. They also discuss techniques to minimize fetal risk during radiation therapy and the timing of treatment initiation. Furthermore, Expert 1 highlights the importance of considering the patient's personal priorities and addressing potential effects on the fetus and the patient's ability to manage treatment-related morbidities during pregnancy and with a newborn.

Although my suggestion aligns most closely with Expert 1's recommendation, I still emphasize the importance of a multidisciplinary evaluation and considering alternative treatment options, as mentioned by Expert 4, to ensure the best possible outcome for both the mother and the fetus. The final decision should be made after thorough discussion and collaboration among the patient, oncologists, and maternal-fetal medicine specialists.

User: *After seeing the other experts' recommendations, are you going to update your own recommendation of your most favored therapeutic approach in this patient case? If so, what is your updated recommendation and which expert/experts is your update based on?*

ChatGPT-4: After reviewing the recommendations of the other four experts, I still believe my initial recommendation is a well-balanced approach that considers the patient's cancer management and the potential risks to the fetus. However, I would like to emphasize a few points from the other experts' recommendations that could further refine my approach:

Expert 1 and Expert 2 both discuss the importance of considering the fetal dose when selecting a radiation therapy technique. I concur that final decisions regarding the radiation therapy technique should be made after estimating the fetal dose and considering the potential risks.

Expert 2 suggests using 3-dimensional conformal radiotherapy (3D-CRT) over intensity-modulated radiation therapy (IMRT) due to concerns about internal scatter increasing the fetal dose. This is a valid consideration and should be discussed with the radiation oncologist when planning the treatment.

Expert 3 highlights the uncertainty in the magnitude of the benefit of radiation therapy during pregnancy, while Expert 4 discusses the possibility of considering adjuvant chemotherapy in the interval between resection and radiation. I agree that this could be an option to explore, but the decision should be made after a thorough discussion with the patient and medical oncologist, weighing the potential risks and benefits.

In summary, my updated recommendation maintains the initial approach but emphasizes the importance of carefully selecting the radiation therapy technique based on fetal dose estimations and considering the use of adjuvant chemotherapy to minimize the risk of interval recurrence due to radiation delay. These refinements are based on insights from all four experts and aim to optimize cancer management while minimizing risks to the fetus.
