# Supplementary material for: Benchmarking ChatGPT-4 on a radiation oncology in-training exam and Red Journal Gray Zone cases: potentials and challenges for ai-assisted medical education and decision making in radiation oncology
Source: Front Oncol. 2023 Sep 14;13:1265024. doi: 10.3389/fonc.2023.1265024 (PMC10543650; doi:10.3389/fonc.2023.1265024)
Supplement: Supplementary file 2 [file DataSheet_2.zip › Red journal gray zone evaluation/Case4_with_GPT_response.pdf]

GRAY ZONE

CME ASTRO

INTERNATIONAL JOURNAL OF
RADIATION ONCOLOGY • BIOLOGY • PHYSICS

www.redjournal.org

From the Plexus to the Cord: A Case of Metastatic Breast Cancer

Chidinma P. Anakwenze Akinfenwa, MD, MPH, and Eric A. Strom, MD

Department of Radiation Oncology, The University of Texas MD Anderson Cancer Center, Houston, Texas

Received Nov 17, 2020; Accepted for publication Jan 12, 2021

In 1998, a 46-year-old professional model presented with breast cancer with axillary, infraclavicular, liver, and lung metastasis. She completely responded to systemic therapy but developed brain metastasis treated in 1999 with resection and scalp-sparing whole brain radiation. She continued modeling and remained with no evidence of disease before recurring locally and undergoing lumpectomy and radiation in 2002. After a breast and infraclavicular recurrence, she received chemotherapy followed by modified radical mastectomy in 2005. She remained with no evidence of disease until a positron emission tomography/computed tomography scan in 2013 (Fig. 1a) revealed curvilinear activity in the left infraclavicular region thought to be nodal or vascular phenomena. She developed slowly progressive sensory deficits, pain, and mild weakness in the left forearm. Repeat positron emission tomography/computed tomography scan in 2014 (Fig. 1b) revealed progressive linear uptake concerning for infiltration of the brachial plexus (BP) cords. This prompted electromyography, which confirmed plexopathy. Given suspicion of metastatic BP infiltration, the infraclavicular and supraclavicular regions were treated to 40 Gy in 15 fractions, followed by BP boost of 10 Gy in 5 fractions completed in January 2015 with radiographic response (Fig. 1c). Her weakness remained

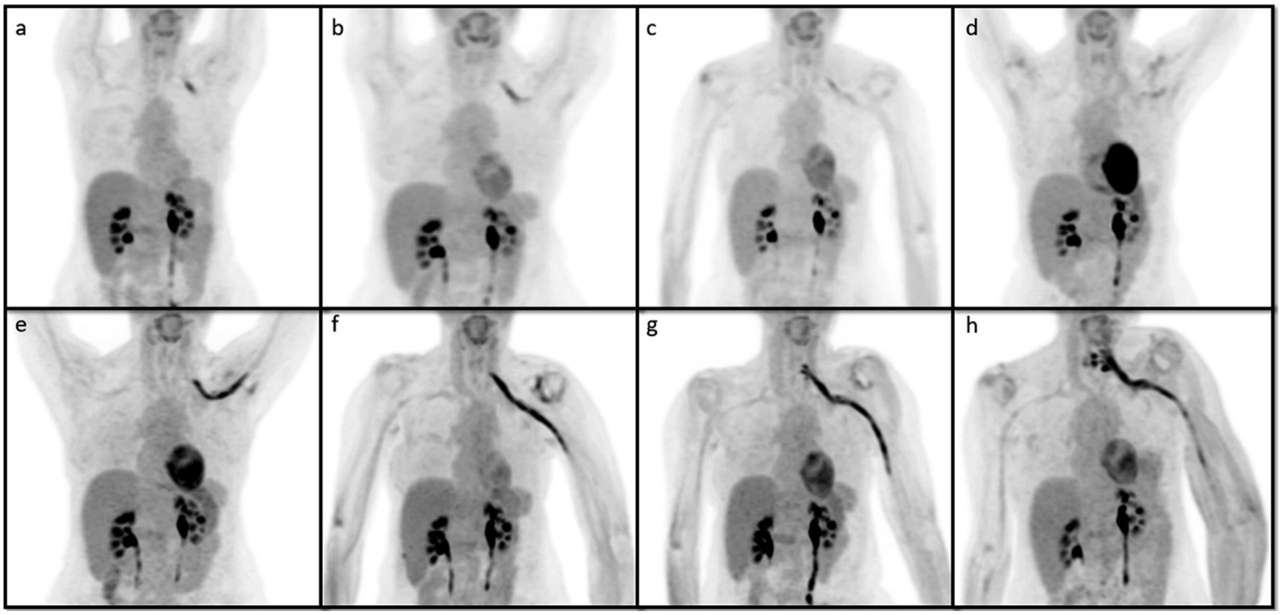

Fig. 1. Chronological progression of brachial plexus metastasis dated (a) December 2013, (b) September 2014, (c) January 2015, (d) June 2015, (e) January 2016, (f) October 2016, (g) May 2017, and (h) May 2018

Int J Radiation Oncol Biol Phys, Vol. 114, No. 2, pp. 181−182, 2022
0360-3016/$ - see front matter © 2021 Published by Elsevier Inc.
https://doi.org/10.1016/j.ijrobp.2021.01.020

stable, but imaging revealed slow progression extending to the cervical and thoracic spinal cord, which acutely resulted in ambulatory deficits in November 2018. We thus emergently irradiated her cord with 40 Gy in 15 fractions.

- Given that there are other causes of brachial plexopathy and lower extremity weakness, what diagnostic tools might aid in distinguishing neurotrophic tumor infiltration?
- If radiation of the BP and/or cord is recommended, what dose and fractionation is recommended?

∗Corresponding author: Chidinma P. Anakwenze Akinfenwa, MD, MPH; E-mail: cpanakwenze@mdanderson.org
Disclosures: none.
CME is available for this feature as an ASTRO member benefit, to access visit https://academy.astro.org.
Reprint requests to: Chidinma P. Anakwenze Akinfenwa, MD, MPH, U.T.-M.D. Anderson Cancer Center, Department of Radiation Oncology, 1515 Holcombe Blvd., Houston, Texas 77030, Tel: (713) 792-6161; E-mail: cpanakwenze@mdanderson.org
Conflict of interest: none.

INTERNATIONAL JOURNAL OF
RADIATION ONCOLOGY • BIOLOGY • PHYSICS
www.redjournal.org

GRAY ZONE EXPERT OPINIONS

High Reward and Low Risk

Clinical history in combination with ultrasound, magnetic resonance imaging, and positron emission tomography (PET) can assess whether symptoms are due to neurotrophic tumor infiltrate versus radiation plexopathy or another cause. In this patient[1] with infraclavicular disease and prior radiation, symptoms are likely from direct tumor involvement or prior radiation.

Clinically, painless upper trunk lesions with lymphedema correlate with radiation injury, whereas painful lower trunk lesions are more often related to neoplastic infiltration. On magnetic resonance imaging, both radiation plexopathy and tumor appear as hypointense on T1 and have increased T2 signal. On PET, radiation injury is only mildly avid, whereas tumor is often hypermetabolic.[2] The PET avidity and pain in this patient is consistent with tumor.

Increased dose correlates with pain relief in patients with carcinomatous plexopathy. A biologically effective dose (alpha/beta = 10) over 30 is associated with higher rates of treatment response. Recently published large randomized trials of conventional versus hypofractionated treatment for postmastectomy radiation therapy have shown no evidence of brachial plexus injury in patients who received hypofractionation, with a median follow-up of 5 years. Therefore, the dose of 40 Gy in 15 fractions used for this patient seems appropriate for palliation, with minimal risk.

With regard to the optimal dose for reirradiation, there is data to suggest that risk of toxicity to the plexus is low in patients with a >2-year interval between radiation therapy courses.[3] The same authors recommend a cumulative maximum dose of less than 95 Gy. As such, the reported dose of 40 Gy in 15 fractions appears safe and reasonable.

Melissa Pulfer Mitchell, MD, PhD
University of Texas
MD Anderson Cancer Center
Houston Texas

Disclosures: none.

Int J Radiation Oncol Biol Phys, Vol. 114, No. 2, pp. 183−184, 2022
0360-3016/$ - see front matter © 2021 Elsevier Inc. All rights reserved.

References

1. Anakwenze Akinfenwa CP, Strom EA. A case of metastatic breast cancer to the brachial plexus with direct infiltration of the intramedullary cervical and thoracic spinal cord. *Int J Radiat Oncol Biol Phys* 2022;114:181–182.
2. Gilcrease-Garcia BM, Deshmukh SD, Parsons MS. Anatomy, imaging, and pathologic conditions of the brachial plexus. *Radiographics* 2020; 40:1686–1714.
3. Chen AM, Yoshizaki T, Velez MA, et al. Tolerance of the brachial plexus to high-dose reirradiation. *Int J Radiat Oncol Biol Phys* 2017;98:83–90.

https://doi.org/10.1016/j.ijrobp.2021.03.011

Ten Percent Plexopathy Is Acceptable in This Case

Metastatic infiltration of breast cancer into the brachial plexus (BP) is rare. Symptoms can occur years after initial diagnosis and can be confused with treatment-related complications. Diagnostic workup can include electromyography, BP magnetic resonance imaging (MRI), positron emission tomography with computed tomography and/or nerve biopsy to ensure accurate diagnosis. The gold standard in this setting is MRI, with a sensitivity of 95% for detecting perineural invasion and 63% for mapping the entire extent of perineural spread.[1] The clinical target volume of involved nerves can be extended proximally to their site of origin—in this case, at the spinal cord levels C5 to T1.

At the time of initial BP involvement, 40 Gy in 15 fractions (equivalent dose in 2-Gy fractions ($EQD2_3$) of 45.3 Gy) followed by a boost of 10 Gy in 5 fractions ($EQD2_3$ of 10 Gy) was delivered. Assuming an α/β ratio of 3, an initial, more fractionated regimen such as 60 Gy in 30 fractions may have allowed for dose escalation with minimally increased risk of toxicity.[2] Of note, estimates of the α/β ratio of breast cancer range from 2.2 Gy to 10.0 Gy.[3]

At time of recurrence, if accepting a 10% chance of BP injury, a total $EQD2_3$ of 90 Gy could be delivered to the BP.[4] This would leave an $EQD2_3$ of 35 Gy that could be prescribed to the BP this time. This could be delivered as 34 Gy in 15 fractions to the BP with a

simultaneous integrated boost of 40 Gy in 15 fractions to the nonoverlapping area of spinal cord. Admittedly, the use of the linear quadratic equation to estimate cumulative toxicity risk is imperfect, but given the inevitable plexopathy that may arise without further radiation therapy, this dose and fractionation with a relatively low risk of radiation-induced toxicity warrants consideration.

In summary, it is our opinion that the authors[5] used an appropriate dose of radiation up front that straddled palliative and curative-intent regimens in terms of its overall risk and efficacy, which appears appropriate for this patient with apparent oligoprogression. In hindsight given the recurrence, an initial aggressive, conventionally fractionated regimen encompassing a larger portion of the BP may have optimized outcomes, although with a slightly higher risk of radiation-induced plexopathy.

Sangjune Laurence Lee, MD, MSE, FRCPC
Salman Faruqi, MD, FRCPC
Division of Radiation Oncology
Tom Baker Cancer Center
University of Calgary
Calgary, Alberta, Canada

Disclosures: I (Salman Faruqi) have received funding from Sanofi for educational events regarding the management of cutaneous malignancy.
Acknowledgments—We thank Jeffrey Q. Cao, MD, MBA, FRCPC, for this opportunity and his comments that greatly improved the article.

References

1. Nemzek WR, Hecht S, Gandour-Edwards R, et al. Perineural spread of head and neck tumors: How accurate is MR imaging? *AJNR Am J Neuroradiol* 1998;19:701–706.
2. Yan M, Kong W, Kerr A, et al. The radiation dose tolerance of the brachial plexus: A systematic review and meta-analysis. *Clin Transl Radiat Oncol* 2019;18:23–31.
3. van Leeuwen CM, Oei AL, Crezee J, et al. The alfa and beta of tumours: A review of parameters of the linear-quadratic model, derived from clinical radiotherapy studies. *Radiat Oncol* 2018;13:96.
4. Morse RT, Doke K, Ganju RG, et al. Stereotactic body radiation therapy for apical lung tumors: Dosimetric analysis of the brachial plexus and preliminary clinical outcomes. *Pract Radiat Oncol* 2022;12:e183–e192.
5. Anakwenze Akinfenwa CP, Strom EA. A case of metastatic breast cancer to the brachial plexus with direct infiltration of the intramedullary cervical and thoracic spinal cord. *Int J Radiat Oncol Biol Phys* 2022;114:181–182.

https://doi.org/10.1016/j.ijrobp.2022.07.020

Don't Forget the Value of a Good History

Differentiating malignant infiltration from radiation induced fibrosis can be challenging so taking a comprehensive history, particularly noting the previous radiation dose to the brachial plexus (BP) and time elapsed since treatment is key.[1] Clinical presentation with both sensory and motor neurologic deficits can point more toward a malignant cause, as can intractable pain at rest and the tempo of progression. The evolution of fibrosis is a slow process.[2] A dedicated magnetic resonance imaging BP protocol is useful to aid the clinical diagnosis. Malignant infiltration is typically less uniform in appearance compared with the linear enhancement of radiation induced fibrosis, which typically presents without a focal mass.

The intent of treatment, projected life expectancy, specifically whether late toxicity will have time to develop, and time interval between radiation courses all help inform the organ at risk constraints. Development of plexopathy is affected not only by total dose and dose/fraction but the volume of the BP receiving a high dose of radiation, the use of concomitant chemotherapy, and whether plexopathy was present at the start of treatment. Most protocols limit the BP dose to between 60 to 66 $Gy_{2/2}$ with increased risk of toxicity at doses greater than 69 Gy.[3] Chen et al[4] reported increased tolerance of the BP to historically accepted constraints in the setting of reirradiation. A clinical decision may need to be made, weighing the risk that plexopathy might be an inevitable consequence of disease progression should the target volume not be adequately covered to meet constraints. These decisions are multifactorial and complex and need to be made with the patient. In the setting of reirradiation of the spinal cord using conventional fractionation, Nieder et al[5] showed that the risk of radiation myelopathy is low, providing the cumulative biologically effective dose is $\leq$135.5 Gy_2, the interval between radiation courses is at least 6 months, and the dose of each course is $\leq$98 Gy_2.

Emma Maria Dunne, PhD, MBBS, FRCR
Department of Radiation Oncology,
British Columba Cancer Agency
Vancouver Centre
British Columbia, Canada

References

1. Anakwenze Akinfenwa CP, Strom EA. A case of metastatic breast cancer to the brachial plexus with direct infiltration of the intramedullary cervical and thoracic spinal cord. *Int J Radiat Oncol Biol Phys* 2022;114:181–182.
2. Johansson S, Svensson H, Denekamp J. Timescale of evolution of late radiation injury after postoperative radiotherapy of breast cancer patients. *Int J Radiat Oncol Biol Phys* 2000;48:745–750.
3. Amini A, Yang J, Williamson R, et al. Dose constraints to prevent radiation-induced brachial plexopathy in patients treated for lung cancer. *Int J Radiat Oncol Biol Phys* 2012;82:e391–e398.
4. Chen AM, Yoshizaki T, Velez MA, Mikaeilian AG, Hsu S, Cao M. Tolerance of the brachial plexus to high-dose reirradiation. *Int J Radiat Oncol Biol Phys* 2017;98:83–90.
5. Nieder C, Grosu AL, Andratschke NH, Molls M. Update of human spinal cord reirradiation tolerance based on additional data from 38 patients. *Int J Radiat Oncol Biol Phys* 2006;66:1446–1449.

https://doi.org/10.1016/j.ijrobp.2022.06.060

ChatGPT-4: Systemic Therapy and Supportive Care

User: *You are an expert radiation oncologist from an academic center.*

Please give your recommendation on the following case:

"…"

Please give a specific recommendation of your most favored therapeutic approach in this patient case based on the available information and your reasoning for it.

ChatGPT-4: Based on the available information, this patient has shown a history of recurrent metastatic breast cancer with involvement of various sites, including the brachial plexus, and a prior history of multiple treatments, including systemic therapy, surgery, and radiation.

My most favored therapeutic approach for this patient would be a combination of systemic therapy and supportive care. I would first recommend a thorough multidisciplinary evaluation involving a medical oncologist, radiation oncologist, and neurologist to tailor the best treatment plan.

Reasoning:

The patient has shown a tendency for recurrence and metastasis, which might imply the presence of resistant cancer cells. Systemic therapy, such as targeted therapy or immunotherapy, may be more effective in controlling the spread of the disease and targeting cancer cells that are resistant to previous treatments. Additionally, genomic profiling of the tumor can guide the selection of targeted therapies.

Reirradiation of the brachial plexus could be considered; however, given the patient's prior radiation treatments, the risk of toxicity and complications may be higher. A thorough assessment of the benefits and risks of reirradiation should be discussed with the radiation oncologist.

Supportive care is essential for this patient due to the sensory deficits, pain, and mild weakness in the left forearm. This may involve pain management, physical therapy, and occupational therapy to improve the patient's quality of life and maintain functionality.

Close monitoring and regular follow-ups are necessary to track the disease progression and modify the treatment plan accordingly.

It is essential to have open communication with the patient about the goals of the treatment, the potential benefits and risks, and the importance of a balanced approach that considers both disease control and quality of life.

User: *Given that there are other causes of brachial plexopathy and lower extremity weakness, what diagnostic tools might aid in distinguishing neurotrophic tumor infiltration?*

ChatGPT-4: Distinguishing neurotrophic tumor infiltration from other causes of brachial plexopathy and lower extremity weakness requires a combination of diagnostic tools. Some of the key diagnostic methods include:

Imaging studies:

a. Magnetic Resonance Imaging (MRI): MRI with and without contrast is the preferred imaging modality for visualizing the brachial plexus and spinal cord. It provides detailed information about soft tissue structures and can help identify tumor infiltration, inflammation, or other structural abnormalities.
b. Positron Emission Tomography/Computed Tomography (PET/CT): PET/CT can detect areas of increased metabolic activity associated with cancerous lesions, aiding in the identification of metastatic infiltration.

Electromyography (EMG) and nerve conduction studies (NCS): These tests assess the electrical activity of muscles and the conduction of impulses along the nerves. They can help distinguish between nerve and muscle disorders, localizing the site of the lesion and determining the severity and extent of the plexopathy.

Cerebrospinal fluid (CSF) analysis: A lumbar puncture can be performed to obtain CSF for analysis. The presence of malignant cells, elevated protein levels, or other abnormal findings may suggest tumor infiltration or other neurological conditions.

Biopsy: In some cases, when imaging and other diagnostic tests are inconclusive, a biopsy of the suspected lesion or surrounding tissue may be necessary to confirm the presence of malignant cells.

Blood tests: Certain tumor markers or inflammatory markers in the blood may provide additional information and help distinguish between cancer-related and non-cancer-related causes of plexopathy or weakness.

A comprehensive assessment using these diagnostic tools, along with a thorough clinical examination and a review of the patient's medical history, can help distinguish neurotrophic tumor infiltration from other causes of brachial plexopathy and lower extremity weakness.

User: *If radiation of the BP and/or cord is recommended, what dose and fractionation is recommended?*

ChatGPT-4: As a radiation oncologist, I would advise caution in reirradiating the brachial plexus (BP) and/or spinal cord, given the patient's prior radiation treatments and the risk of toxicity and complications. However, if reirradiation is determined to be the best course of action after a

multidisciplinary evaluation, a personalized approach based on the patient's medical history and the location and extent of the tumor should be taken.

For reirradiation of the brachial plexus, a common approach would be to use a hypofractionated regimen, which consists of administering higher doses of radiation per fraction over a shorter period of time. One potential regimen for this patient could be 30-35 Gy in 10-15 fractions. This approach aims to minimize the risk of radiation-induced complications while still delivering an adequate dose to control the disease.

For reirradiation of the spinal cord, the situation becomes more challenging due to the risk of radiation myelopathy, a rare but potentially severe complication that can result in paralysis. The maximum recommended cumulative dose for the spinal cord is typically around 50 Gy, although the actual dose tolerance may vary depending on factors such as previous radiation treatments and the patient's overall health.

If reirradiation is deemed necessary, it is crucial to use advanced radiation planning techniques such as intensity-modulated radiation therapy (IMRT) or stereotactic body radiotherapy (SBRT), which allow for highly conformal dose delivery to the target while sparing the surrounding healthy tissue.

The specific dose and fractionation for this patient should be determined by the radiation oncologist, taking into account the patient's previous treatments, the time elapsed since prior radiation therapy, the volume and location of the tumor, and the risks and benefits associated with reirradiation. Regular follow-up and close monitoring of the patient's response to treatment are essential to ensure the best possible outcome while minimizing potential complications.

***User:** Please summarize the other experts' recommendations in short sentences.*

ChatGPT-4:

Expert 1: Focuses on clinical history and imaging to assess the cause of symptoms, supports a biologically effective dose (alpha/beta = 10) over 30 for treatment response, and considers the previously administered dose of 40 Gy in 15 fractions safe and reasonable for the patient.

Expert 2: Suggests accepting a 10% chance of BP injury, allowing for a total EQD2(3) of 90 Gy to be delivered to the BP, with the remaining EQD2(3) of 35 Gy prescribed this time, and delivered as 34 Gy in 15 fractions to the BP and 40 Gy in 15 fractions to the nonoverlapping area of the spinal cord.

Expert 3: Emphasizes the importance of a comprehensive history and clinical presentation in differentiating malignant infiltration from radiation-induced fibrosis, and stresses the need for complex, multifactorial decision-making, considering factors such as treatment intent, projected life expectancy, and the time interval between radiation courses.

User: *As an expert radiation oncologist, which of the above four experts' recommendation do you think the most proper?*

ChatGPT-4: As an expert radiation oncologist, I find the recommendations provided by all three experts valuable. However, I believe that Expert 1's recommendation seems to be the most appropriate in this case, given the specific circumstances of the patient.

Expert 1 focuses on the use of clinical history, imaging studies, and correlating symptoms to determine the cause of the patient's symptoms. They support the use of a biologically effective dose (alpha/beta = 10) over 30 for treatment response and emphasize the safety of the previously administered dose of 40 Gy in 15 fractions for this patient.

Additionally, Expert 1 acknowledges the importance of the interval between radiation therapy courses and the cumulative maximum dose in determining the optimal dose for reirradiation. They find the reported dose of 40 Gy in 15 fractions to be safe and reasonable for this patient, considering the patient's history and current condition.

Overall, Expert 1's recommendation focuses on providing effective palliation for the patient while minimizing the risk of radiation-induced complications. They carefully consider the patient's history, interval between radiation therapy courses, and the cumulative maximum dose to guide their recommendation.

That being said, it is important to recognize that each patient's situation is unique, and a multidisciplinary evaluation with the input of radiation oncologists, medical oncologists, surgeons, and other healthcare professionals is crucial for determining the best course of action for each individual.

User: *Your most favored therapeutic approach for this patient would be a combination of systemic therapy and supportive care. You would first recommend a thorough multidisciplinary evaluation involving a medical oncologist, radiation oncologist, and neurologist to tailor the best treatment plan. In this case, which expert's recommendation is your suggestion closest to?*

ChatGPT-4: My earlier recommendation of focusing on Expert 1's approach does not contradict the idea of combining systemic therapy and supportive care in this patient's case. Expert 1's recommendation primarily addresses the radiation therapy aspect of treatment, but it is important to note that their approach does not exclude the possibility of integrating systemic therapy and supportive care.

The decision to combine systemic therapy and supportive care should be made after a thorough multidisciplinary evaluation involving a medical oncologist, radiation oncologist, and neurologist, as I previously mentioned. Each expert's recommendation provides valuable insights, but Expert 1's approach to radiation therapy seems most appropriate in this case, given the specific circumstances of the patient. This approach can be integrated into a comprehensive treatment plan that also incorporates systemic therapy and supportive care, as deemed appropriate by the multidisciplinary team.

User: *After seeing the other experts' recommendations, are you going to update your own recommendation of your most favored therapeutic approach in this patient case? If so, what is your updated recommendation and which expert/experts is your update based on?*

ChatGPT-4: After reviewing the recommendations of the other three experts, I would like to update my recommendation for this patient case.

Updated Recommendation:

Diagnostic workup: Perform a thorough diagnostic workup to differentiate between malignant infiltration and radiation-induced plexopathy, including clinical history, dedicated magnetic resonance imaging (MRI) of the brachial plexus, and positron emission tomography with computed tomography (PET-CT) as suggested by Expert 1 and Expert 2.

- Reirradiation: Considering the recommendations of Expert 1 and Expert 2, reirradiation of the brachial plexus with a dose of 34 Gy in 15 fractions, along with a simultaneous integrated boost of 40 Gy in 15 fractions to the non-overlapping area of the spinal cord, may be appropriate. The risk of radiation-induced toxicity is relatively low and could be acceptable given the potential for inevitable plexopathy without further radiation therapy.

- Systemic therapy: As mentioned in my initial recommendation, consider systemic therapy options such as targeted therapy or immunotherapy based on genomic profiling of the tumor to control disease spread and target resistant cancer cells.

- Supportive care: Continue with supportive care measures, including pain management, physical therapy, and occupational therapy to maintain the patient's quality of life and functionality.

- Monitoring and follow-ups: Perform close monitoring and regular follow-ups to track disease progression and adjust the treatment plan accordingly.

This updated recommendation is based on the valuable insights from Expert 1, Expert 2, and Expert 3, which helped refine the diagnostic workup, reirradiation plan, and overall treatment strategy. The importance of a comprehensive history, proper diagnostic imaging, and weighing the risks and benefits of reirradiation were particularly informative in updating the recommendation.
