# Supplementary material for: Benchmarking ChatGPT-4 on a radiation oncology in-training exam and Red Journal Gray Zone cases: potentials and challenges for ai-assisted medical education and decision making in radiation oncology
Source: Front Oncol. 2023 Sep 14;13:1265024. doi: 10.3389/fonc.2023.1265024 (PMC10543650; doi:10.3389/fonc.2023.1265024)
Supplement: Supplementary file 2 [file DataSheet_2.zip › Red journal gray zone evaluation/Case5_with_GPT_response.pdf]

GRAY ZONE

INTERNATIONAL JOURNAL OF
RADIATION ONCOLOGY • BIOLOGY • PHYSICS

www.redjournal.org

Low Risk, High Risk: Adjuvant Therapy in Resected p16+ Oropharyngeal Cancer with ≥5 Positive Ipsilateral Lymph Nodes

Bailey Nelson, MD, and Vinita Takiar, MD, PhD

Department of Radiation Oncology, University of Cincinnati, Cincinnati, Ohio

A 50-year-old, previously healthy man presented with a 2-month history of an enlarging left neck mass and odynophagia. He is a never smoker. Physical examination revealed a mobile 2.5 cm left level 2 cervical lymph node. Fine needle aspiration of the node was positive for p16+ squamous cell carcinoma. Positron emission tomographic computed tomography demonstrated at least 2 avid left level 2 cervical lymph nodes with no obvious primary lesion and no distant metastatic disease (Fig 1). Quadroscopy revealed an abnormal lesion in the left palatine tonsil and biopsy was positive for p16+ squamous cell carcinoma. There was no extension of the tonsillar mass to the base of tongue or soft palate. The clinical stage was T1N1M0. The patient proceeded with transoral robotic surgery tonsillectomy and selective lymph node dissection of the left neck (levels 2-4). Two level 2 cervical lymph nodes and 5 level 3 cervical lymph nodes were positive for metastatic carcinoma out of a total of 36 lymph

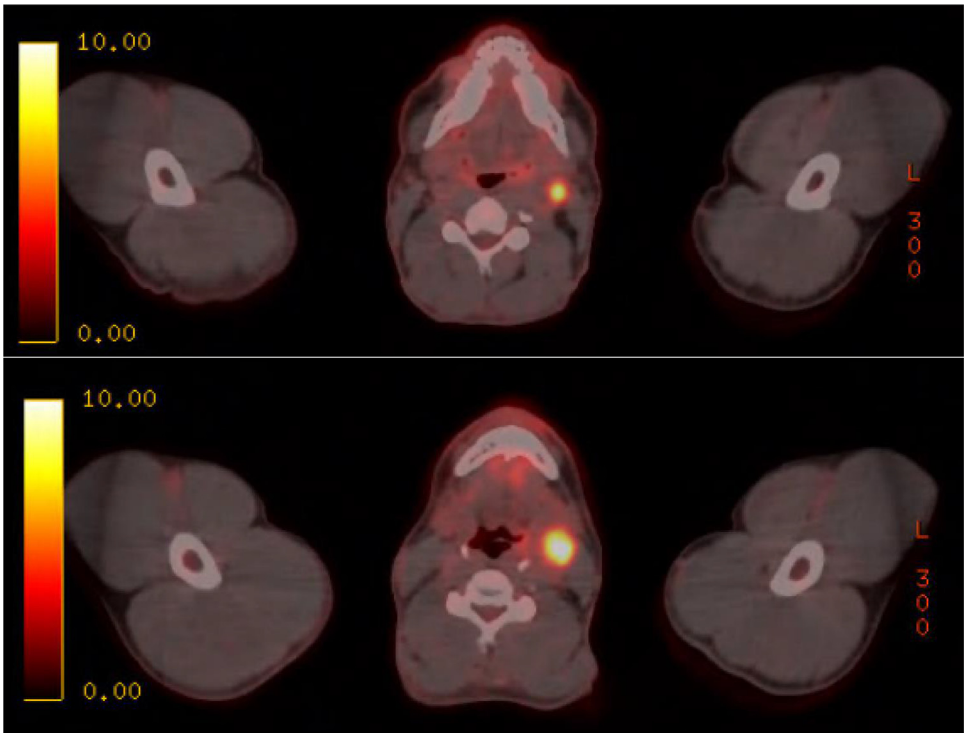

Fig. 1. Staging positron emission tomographic computed tomography.

nodes with no evidence of extranodal extension. Pathology from the left tonsil specimen confirmed a 1.5 cm p16+ squamous cell carcinoma with negative surgical margins and no lymphovascular or perineural invasion. The pathologic stage was T1N2.

Int J Radiation Oncol Biol Phys, Vol. 114, No. 1, pp. 10−11, 2022
0360-3016/$ - see front matter Published by Elsevier Inc.
https://doi.org/10.1016/j.ijrobp.2022.04.018

Questions

What are your recommendations for adjuvant therapy?

1. Adjuvant radiation or adjuvant chemoradiation?
2. Ipsilateral or bilateral nodal radiation?
3. What radiation dose(s) should be prescribed?
4. Should the primary site be irradiated?

INTERNATIONAL JOURNAL OF
RADIATION ONCOLOGY • BIOLOGY • PHYSICS
www.redjournal.org

GRAY ZONE EXPERT OPINION

Multistation Radiation

Over a decade has passed since the prognostic significance of human papillomavirus—status for squamous cell cancers of the oropharynx was demonstrated. Numerous cooperative group efforts have investigated both dose and volume treatment de-escalation in these patients. For this case, in the setting of upfront surgical management with a transoral robotic surgery (TORS)/selective neck approach, given pathologic confirmation of multistation ipsilateral neck disease, we unequivocally recommend adjuvant therapy. In absence of positive margins or extranodal extension, we do not advocate for chemoradiation but instead radiation alone. We favor irradiation of both the primary site and ipsilateral neck. Modern protocols, such as NRG HN-005, allow for unilateral neck treatment in the definitive setting for select patients with well-lateralized primary tumors with minimal nodal disease burden. Although this patient has both level II and III involvement, in the setting of an adequate neck dissection, we are comfortable omitting the contralateral neck. Although there is emerging evidence for uncoupling the primary site from the neck for post-TORS radiation therapy treatment, this paradigm remains in its infancy and lacks randomized validation. Further, omission of the primary site when delivering ipsilateral neck radiation after TORS appears to offer no significant clinical or dosimetric advantage.[1] Based on the evidence from the ECOG-ACRIN 3311 clinical trial, this patient fails to meet criteria for the intermediate-risk group (T1-2 negative/close margins, N1-2 with <1 mm ENE and ≤4 lymph nodes +) and thus is questionable for de-escalated radiation doses.[2] Thus, we recommend treating this patient's operative bed and involved level II and III neck nodal levels to a dose of 60 Gy with elective coverage of the ipsilateral level IV neck to 54 Gy in 30 fractions with intensity-modulated radiotherapy.

Grant W McKenzie, MD
Neal E Dunlap, MD
Department of Radiation Oncology
University of Louisville
Louisville, Kentucky

Disclosures: none.

References

1. Lazarev S, Todorov B, Tam J, et al. Adjuvant radiation in the TORS era: Is there a benefit to omitting the tumor bed? *Pract Radiat Oncol* 2017;7:93–99.
2. Ferris RL, Flamand Y, Weinstein G, et al. Phase II randomized trial of transoral surgery and low-dose intensity modulated radiation therapy in resectable p16+ locally advanced oropharynx cancer: An ECOG-ACRIN Cancer Research Group trial (E3311). *J Clinc Oncol* 2022;40:138–149.

https://doi.org/10.1016/j.ijrobp.2022.04.034

Less Is Not Always More

First, pretreatment patient selection for surgical resection of oropharynx cancer is of paramount importance, as patients receiving triple modality therapy consistently report higher levels of toxicity, worse quality of life, higher decision regret, and value cure as first priority both pre- and posttreatment. The accuracy of imaging to predict pathologic nodal stage has been variable in the literature. Postoperatively, the patient in question would be considered "intermediate risk" by several clinical trial stratifications, but likely carries a clinically significant risk of both locoregional failure and distant metastases.

In consideration of adjuvant radiation therapy (RT) for this patient, we would not typically give less than 60 Gy postoperatively at our institution. Although pN2 patients have been included in de-escalation trial strategies as "intermediate risk" with only pathologic extranodal extension considered at highest risk, the literature suggests that patients with high nodal count have adverse outcomes. Specifically, OS, PFS, LRC, and DM have been shown to be worse even in the absence of extranodal extension, with the potential for out-of-field locoregional failures in the setting of RT volume de-escalation.[1-4] The present patient could also be considered for concurrent chemotherapy for these reasons. A proper noninferiority trial to define optimal adjuvant RT dose

0360-3016/$ - see front matter © 2022 Published by Elsevier Inc.

for intermediate-risk patients seems unlikely, and our field continues to struggle with which patients may be treated with less RT, but this patient likely represents an extreme within the intermediate-risk category.

Although the primary site was resected with negative margins, our institution has not considered the promising phase II data of the AVOID trial yet standard of care, and thus would typically treat both the primary tumor site and the neck. We would expect a primary tumor confined to the anatomic tonsil to drain to the ipsilateral neck only, but, in this particular patient, we would also carefully evaluate and consider treating the contralateral neck. Although we do not use multiple nodes or N2b status (AJCC 7th ed) by itself as an automatic indication for contralateral neck irradiation, this patient had a total of 7 lymph nodes involved in the ipsilateral neck with 5 radiographically occult lymph nodes, likely representing aggressive biology. One must consider the balance of tumor control and toxicity when making the decision to treat the contralateral neck. In our practice, even with bilateral neck RT, we have been able to spare the pharyngeal constrictors and salivary glands to below meaningful dose constraints to minimize toxicity.

Michelle Mierzwa, MD
Caitlin Schonewolf, MD
Department of Radiation Oncology
University of Michigan
Ann Arbor, Michigan

Disclosures: nonePlease verify that all disclosures are complete or add any missing disclosure information..

2. Lee NCJ, Kelly JR, Park HS, et al. Patterns of failure in high-metastatic node number human papillomavirus-positive oropharyngeal carcinoma. *Oral Oncol* 2018;85:35–39.
3. Loganadane G, Kelly JR, Lee NC, et al. Incidence of radiographically occult nodal metastases in HPV+ oropharyngeal carcinoma: Implications for reducing elective nodal coverage. *Pract Radiat Oncol* 2018;8:397–403.
4. Zhan KY, Eskander A, Kang SY, et al. Appraisal of the AJCC 8th edition pathologic staging modifications for HPV-positive oropharyngeal cancer, a study of the National Cancer Data Base. *Oral Oncol* 2017;73:152–159.

https://doi.org/10.1016/j.ijrobp.2022.06.069

Chemotherapy Only for High-risk Features

A 50-year-old never smoker presented with p16+ squamous cell carcinoma, confined to the tonsil with 2 avid lymph nodes in the ipsilateral neck, both less than 3 cm. With these pretreatment findings, an initial approach with surgery was almost assured of requiring adjuvant radiation therapy. Two recently published phase 2 randomized trials of surgery or radiation therapy[1,2] in such patients suggest definitive radiation therapy and concurrent systemic therapy may have lower overall morbidity, and this would have been our recommended initial approach.

Although on Eastern Cooperative Oncology Group (ECOG) 3311 the patient would have been assigned to the adjuvant radiation therapy and concurrent cisplatin arm postoperatively,[3] a recent secondary analysis of NRG/Radiation Therapy Oncology Group (RTOG) 9501/0234 and European Organization for Research and Treatment of Cancer (EORTC) 22391 did not find a cutoff on positive lymph nodes where concurrent systemic therapy demonstrated a significantly improved outcome.[4]

Therefore, we would recommend adjuvant radiation therapy without concurrent chemotherapy given the lack of high-risk features of extranodal extension or a positive margin. With a small T1 tonsillar primary tumor that did not involve the tongue base or soft palate, it may be reasonable to avoid contralateral neck radiation. However, for patients not evaluated by a radiation oncologist pretreatment, caution should be exercised with this approach. Although it may be reasonable to spare the postoperative tonsillar bed in the absence of intermediate-risk features, only low-level evidence supports this approach. We would prescribe 60 Gy at 2 Gy per fraction to the tonsillar bed and involved ipsilateral lymph node levels II and III and 54 Gy at 1.8 Gy per fraction to the elective neck levels IV, V, and VIIb using a simultaneous integrated boost technique.

George Y. Yang, MD
Jimmy J. Caudell, MD, PhD
Department of Radiation Oncology
Moffitt Cancer Center
Tampa, Florida

Disclosures: none.

References

1. ACNichols, JTheurer, EPrisman, et al. Radiotherapy versus transoral robotic surgery and neck dissection for oropharyngeal squamous cell carcinoma (ORATOR): An open-label, phase 2, randomised trial. Lancet Oncol2020191349–1359.
2. DAPalma, EPrisman, EBerthelet, et al. Assessment of toxic effects and survival in treatment deescalation with radiotherapy vs transoral surgery for HPV-associated oropharyngeal squamous cell carcinoma: The ORATOR2 phase 2 randomized clinical trial. JAMA Oncol820221–7.
3. RLFerris, YFlamand, GSWeinstein, et al. Phase II randomized trial of transoral surgery and low-dose intensity modulated radiation therapy in resectable p16+ locally advanced oropharynx cancer: An ECOG-ACRIN Cancer Research Group trial (E3311). J Clin Oncol402022138–149.
4. DJLu, MLuu, CGay, et al. Nodal metastasis count and oncologic outcomes in head and neck cancer: A secondary analysis of NRG/RTOG 9501, NRG/RTOG 0234, and EORTC 22931. Int J Radiat Oncol Biol Phys1132022787–795.

https://doi.org/10.1016/j.ijrobp.2022.05.032

Whose Neck Is on the Line?

The landscape of adjuvant treatment in p16+ oropharynx cancers is evolving. This patient has a lateralized p16+ left tonsil squamous cell carcinoma, pT1 pN2 stage II with 5 out of 36 involved lymph nodes, no lymphovascular invasion or perineural invasion, and negative margins. Going from pT1 pN1 to pT1 pN2 changes the 5-year overall survival prognosis from 89% to 70% based on the landmark study by Haughey et al.[1]

Regarding radiation target volumes, the updated Princess Margaret data show excellent outcomes with ipsilateral radiation for well lateralized tonsillar cancers with an aggregate contralateral failure of 4.3% across published series in the setting of multiple neck nodes.[2] A recent series of predominantly Human papillomavirus (HPV)+ tonsil cancers, with 45% having multiple neck nodes, similarly shows 2% contralateral neck failure rate.[3] Although there are intriguing data about omitting coverage of the primary site, this needs further study at the multi-institutional level before becoming standard practice.[4]

In terms of radiation dose, this patient would not have been eligible for the de-escalation arm of Eastern Cooperative Oncology Group 3311 and so we would proceed with standard postoperative dose of 60 Gy. Previous data from our group demonstrate that although pN2 patients are at an elevated risk of both distant and locoregional failure, distant failure predominates.[5] This points to a need to address treatment intensification in these patients in the next generation of clinical trials.

For now, given the elevated risk of failure in this group, for patients young and fit enough to tolerate it, we recommend concurrent chemoradiation with cisplatin for this population.

Nauman H. Malik, MD, MSc
Zain A. Husain, MD
Department of Radiation Oncology, Odette Cancer Centre, Sunnybrook Health Sciences Centre, University of Toronto Toronto, Canada

Disclosures: Z. H. discloses honoraria and travel support from ASTRO and participation in data safety and monitoring board of National Institute of Dental and Craniofacial Research (NIDCR). N. M. has nothing to disclose.

References

1. Haughey BH, Sinha P, Kallogjeri D, et al. Pathology-based staging for HPV-positive squamous carcinoma of the oropharynx. Oral Oncol. 6211–19.
2. Huang SH, Waldron J, Bratman SV, et al. Re-evaluation of ipsilateral radiation for T1-T2N0-N2b tonsil carcinoma at the Princess Margaret Hospital in the human papillomavirus era, 25 years later. Int J Radiat Oncol Biol Phys. 98159–169.
3. Taku N, Chronowski G, Gunn GB, et al. Unilateral radiotherapy for tonsillar cancer: Treatment outcomes in the era of human papilloma virus, positron-emission tomography and intensity-modulated radiation therapy [e-pub ahead of print]. *Int J Radiat Oncol Biol Phys.* doi:10.1016/j.ijrobp.2022.04.035, Accessed on May 4, 2022.
4. Swisher-McClure S, Lukens JN, Aggarwal C, et al. A phase 2 trial of alternative volumes of oropharyngeal irradiation for DE-intensification (AVOID): Omission of the resected primary tumor bed after transoral robotic surgery for human papilloma virus-related squamous cell carcinoma of the oropharynx. Int J Radiat Oncol Biol Phys. 106725–732.
5. Lee NCJ, Kelly JR, Park HS, et al. Patterns of failure in high-metastatic node number human papillomavirus-positive oropharyngeal carcinoma. Oral Oncol. 8535–39.

https://doi.org/10.1016/j.ijrobp.2022.06.058

Too Many Items on the Menu

Published studies on the topic of de-escalation of adjuvant therapy after Transoral Robotic Surgery for p16+ oropharyngeal cancers have a variety of inclusion criteria and proposed de-escalation schemes.

However, not all p16+ cancers are favorable. Though our standard criteria for addition of chemotherapy to postoperative radiation therapy are the presence of extracapsular extension (ECE) and/or positive surgical margins, with 7 lymph nodes (LN) positive (pN2), we would consider the addition of chemotherapy in this patient who met criteria for the chemoradiation arm of ECOG-ACRIN E3311. Despite trimodality therapy, patients on this arm had a 2-year progression free survival of only 90.7%, with the caveat that the majority also had ECE. Other single institution postoperative trials reporting favorable results also employed concurrent chemotherapy for patients with multiple positive LNs, with or without ECE.[1,2]

Although the primary was well-confined, given the number of pathologically positive LNs, which was not manifest on preoperative dual modality imaging, we would default to bilateral LN treatment employing conservative normal tissue constraints with a low threshold for decreasing the elective dose to achieve them.

Little is gained by omission of the primary site and covering the intervening lymphatic tracks giving rise to the involved LNs may be critical.[3]

With regard to dose, we would treat the primary site and involved nodal levels to 60 Gy with any elective LN levels to 50 to 54 Gy using a simultaneous integrated boost technique.

With so many choices for de-escalation on the menu, it is increasingly critical to recognize the patient for whom intensified therapy is most appropriate.

Monica E. Shukla, MD
Musaddiq J. Awan, MD

Department of Radiation Oncology
Medical College of Wisconsin
Milwaukee, Wisconsin

Disclosures: none.

References

1. Miles BA, Posner MR, Gupta V, et al. De-escalated adjuvant therapy after transoral robotic surgery for human papillomavirus-related oropharyngeal carcinoma: The Sinai Robotic Surgery (SIRS) trial. *Oncologist* 2021;26:504–513.
2. Ma DJ, Price KA, Moore EJ, et al. Phase II Evaluation of aggressive dose de-escalation for adjuvant chemoradiotherapy in human papillomavirus-associated oropharynx squamous cell carcinoma. *J Clin Oncol* 2019;37:1909–1918.
3. Lazarev S, Todorov B, Tam J, et al. Adjuvant radiation in the TORS era: Is there a benefit to omitting the tumor bed? *Pract Radiat Oncol* 2017;7:93–99.

https://doi.org/10.1016/j.ijrobp.2022.05.003

ChatGPT-4: Balancing Aggressive Treatment with Minimizing Toxicity

User: *You are an expert radiation oncologist from an academic center.*

Please give your recommendation on the following case:

"…"

Please give a specific recommendation of your most favored therapeutic approach in this patient case based on the available information and your reasoning for it.

ChatGPT-4: Based on the available information, the patient has a p16-positive, squamous cell carcinoma of the left palatine tonsil, staged as T1N2M0. The patient has already undergone transoral robotic surgery (TORS) with tonsillectomy and selective lymph node dissection of the left neck (levels 2-4). The pathology report shows negative surgical margins and no lymphovascular or perineural invasion, which is favorable.

My most favored therapeutic approach for this patient would be adjuvant radiotherapy with concurrent chemotherapy, considering the high-risk factors like multiple lymph node involvement (N2 disease).

The rationale for recommending adjuvant radiotherapy is based on the risk of locoregional recurrence due to the presence of multiple positive lymph nodes. Radiotherapy will be administered to the primary tumor site (tonsil) and the regional lymphatics (bilateral neck, including the dissected and undissected levels) to reduce the risk of recurrence.

Concurrent chemotherapy (most commonly cisplatin) is recommended because it has been shown to improve disease-free survival and overall survival in patients with locally advanced head and neck squamous cell carcinoma, particularly when there are high-risk features like multiple lymph node involvement. The p16-positive status of this patient's tumor is a favorable prognostic factor, as p16-positive oropharyngeal cancers tend to have a better response to treatment and improved outcomes. However, given the N2 nodal status, the addition of chemotherapy is still advisable.

In summary, my recommendation for this patient would be adjuvant radiotherapy with concurrent chemotherapy. This approach aims to minimize the risk of locoregional recurrence and improve overall survival, considering the high-risk features in this case. The patient should be closely followed up during and after the treatment to monitor for potential toxicities and assess the response to therapy.

User: *What are your recommendations for adjuvant therapy? Adjuvant radiation or adjuvant chemoradiation?*

ChatGPT-4:

Apologies for any confusion in my previous response. Based on the available information and the patient's pathological stage of T1N2M0, my recommendation for adjuvant therapy would be adjuvant chemoradiation.

The rationale for choosing adjuvant chemoradiation over adjuvant radiation alone is the presence of high-risk features, such as N2 nodal status with multiple involved lymph nodes. This increases the risk of locoregional recurrence and necessitates the addition of concurrent chemotherapy to improve disease-free survival and overall survival.

Concurrent chemotherapy, most commonly cisplatin, has been shown to enhance the effects of radiation and improve outcomes in patients with locally advanced head and neck squamous cell carcinoma, particularly in the presence of high-risk factors. Although the p16-positive status of the tumor is a favorable prognostic factor, the N2 nodal status warrants the use of concurrent chemotherapy.

In summary, my recommendation for adjuvant therapy in this patient would be adjuvant chemoradiation, as it provides a better chance of disease control and overall survival in the presence of high-risk features such as multiple positive lymph nodes. The patient should be closely monitored during and after treatment for potential toxicities and response to therapy.

***User:** What are your recommendations for adjuvant therapy? Ipsilateral or bilateral nodal radiation?*

ChatGPT-4: Given the patient's pathological stage of T1N2M0, p16-positive squamous cell carcinoma of the left palatine tonsil, and the presence of multiple positive lymph nodes in the left neck, my recommendation for adjuvant therapy would be adjuvant chemoradiation with bilateral nodal radiation.

The rationale for recommending bilateral nodal radiation is to minimize the risk of locoregional recurrence. Although the primary tumor and involved lymph nodes are located on the left side of the neck, there is still a potential risk of subclinical contralateral lymph node involvement in the context of locally advanced disease (N2). Additionally, the anatomical lymphatic drainage pattern of the oropharyngeal region warrants coverage of bilateral neck lymphatics to minimize the risk of occult contralateral nodal disease.

The radiation treatment fields would include the primary tumor site (left tonsil) and the regional lymphatics of both sides of the neck. This would encompass the dissected and undissected lymph node levels on the left side, as well as the contralateral (right) neck lymphatics.

In summary, my recommendation for adjuvant therapy would be adjuvant chemoradiation with bilateral nodal radiation to minimize the risk of locoregional recurrence and improve overall survival in the presence of high-risk features such as multiple positive lymph nodes. The patient should be closely monitored during and after treatment for potential toxicities and response to therapy.

***User:** What radiation dose(s) should be prescribed?*

ChatGPT-4: For this patient with p16-positive squamous cell carcinoma of the left palatine tonsil staged as T1N2M0, who has undergone transoral robotic surgery (TORS) with tonsillectomy and selective lymph node dissection, my recommendation for adjuvant chemoradiation would involve the following radiation doses:

High-risk target volumes (postoperative tumor bed and involved lymph node levels): A total dose of 60-66 Gy, delivered in daily fractions of 1.8-2.0 Gy over 6-6.5 weeks. This dose is prescribed to areas where there is a higher risk of residual microscopic disease, such as the primary tumor bed and involved lymph node regions.

Elective (low-risk) target volumes (uninvolved lymph node levels on both sides of the neck): A total dose of 50-54 Gy, delivered in daily fractions of 1.8-2.0 Gy over 5-5.5 weeks. This dose is prescribed for prophylactic treatment of the regional lymphatics on both sides of the neck, including the undissected contralateral lymph nodes, to address the potential risk of subclinical disease.

The exact dose and fractionation may vary depending on the patient's specific anatomy, proximity of critical structures, and the radiation oncologist's preferences. Intensity-modulated radiation therapy (IMRT) is recommended for better dose conformity and sparing of nearby critical structures, such as the salivary glands, spinal cord, and swallowing muscles.

Concurrent chemotherapy, typically cisplatin, will be administered during the course of radiation therapy to enhance the therapeutic effects of radiation and improve outcomes.

It is important to closely monitor the patient during and after treatment for potential toxicities and response to therapy. In case of significant acute toxicities, dose adjustments or breaks in treatment may be necessary to ensure patient safety and tolerability.

User: *Should the primary site be irradiated?*

ChatGPT-4: Yes, the primary site should be irradiated in this case. The patient has p16-positive squamous cell carcinoma of the left palatine tonsil staged as T1N2M0, and has undergone transoral robotic surgery (TORS) with tonsillectomy and selective lymph node dissection. Despite negative surgical margins and no lymphovascular or perineural invasion, there is still a risk of microscopic residual disease at the primary site.

Including the primary tumor bed (left palatine tonsil region) in the high-risk target volume for radiation therapy helps to minimize the risk of local recurrence. The radiation dose for the high-risk target volumes, including the postoperative tumor bed and involved lymph node levels, should be in the range of 60-66 Gy, delivered in daily fractions of 1.8-2.0 Gy over 6-6.5 weeks.

In addition to the primary site, the regional lymphatics of both sides of the neck should also be irradiated to address the risk of subclinical disease in the lymph nodes, given the presence of multiple positive lymph nodes on the left side of the neck.

Concurrent chemotherapy, typically cisplatin, will be administered during the course of radiation therapy to enhance the therapeutic effects of radiation and improve outcomes.

User: *Please summarize the other experts' recommendations in short sentences.*

ChatGPT-4: Expert 1: Recommends adjuvant radiation therapy (60 Gy to the operative bed and involved lymph node levels; 54 Gy to ipsilateral level IV neck) without chemotherapy, treating the primary site and ipsilateral neck, and omitting the contralateral neck.

Expert 2: Suggests adjuvant radiation therapy (60 Gy or more), possibly with concurrent chemotherapy, treating both the primary site and neck, and considering treatment of the contralateral neck due to aggressive biology.

Expert 3: Advises adjuvant radiation therapy (60 Gy to primary site and involved lymph node levels; 54 Gy to elective neck levels) without concurrent chemotherapy, as there are no high-risk features such as extranodal extension or positive margins.

Expert 4: Recommends concurrent chemoradiation with cisplatin, treating the primary site and ipsilateral neck with standard postoperative dose of 60 Gy.

Expert 5: Supports adjuvant chemoradiation, treating the primary site and bilateral lymph nodes (60 Gy to primary site and involved nodal levels; 50-54 Gy to elective lymph node levels) using a simultaneous integrated boost technique.

User: *As an expert radiation oncologist, which of the above experts' recommendation do you think the most proper?*

ChatGPT-4: As an expert radiation oncologist, I would consider Expert 2's recommendation as the most appropriate for the patient described above. This recommendation takes into account the aggressive biology of the tumor, with a high nodal count, which suggests a higher risk of locoregional failure and distant metastases.

Expert 2 suggests adjuvant radiation therapy of at least 60 Gy, with concurrent chemotherapy as an option to enhance tumor control. They also recommend treating both the primary site and the neck, carefully evaluating and considering treatment of the contralateral neck due to the patient's aggressive tumor biology. This approach aims to strike a balance between tumor control and minimizing toxicity.

It is important to note that individual patient factors, such as comorbidities, performance status, and personal preferences, should also be considered when making treatment decisions. Close

collaboration with the patient's multidisciplinary team, including medical oncologists and head and neck surgeons, is crucial to providing the best possible care.

***User:** At the beginning, you said that "My most favored therapeutic approach for this patient would be adjuvant radiotherapy with concurrent chemotherapy, considering the high-risk factors like multiple lymph node involvement (N2 disease)." In this case, which expert's recommendation is your initial suggestion closest to?*

ChatGPT-4: My initial suggestion is closest to Expert 4's recommendation. Both recommendations emphasize the importance of adjuvant radiotherapy with concurrent chemotherapy due to the aggressive nature of the tumor and the presence of high-risk factors such as multiple lymph node involvement (N2 disease). Additionally, both recommendations suggest treating both the primary site and the neck, with consideration given to the contralateral neck based on the patient's aggressive tumor biology.

***User:** After seeing the other experts' recommendations, are you going to update your own recommendation of your most favored therapeutic approach in this patient case? If so, what is your updated recommendation and which expert/experts is your update based on?*

ChatGPT-4: After considering the recommendations of the other five experts, I would like to update my recommendation for this patient. My updated recommendation would be to proceed with adjuvant radiation therapy, treating both the primary site and the neck. Given the aggressive nature of the tumor, with a high nodal count, I would consider concurrent chemotherapy to enhance tumor control. This approach aims to balance tumor control and minimize toxicity.

Updated recommendation:

- Adjuvant radiation therapy: Treat both the primary site and neck, considering the contralateral neck based on the patient's aggressive tumor biology. Prescribe a dose of at least 60 Gy to the primary site and involved nodal levels, and a lower dose to elective lymph node levels (50-54 Gy).
- Consider concurrent chemotherapy: Evaluate the patient's performance status, comorbidities, and personal preferences before deciding on concurrent chemotherapy to enhance tumor control.

It is essential to consider individual patient factors when making treatment decisions, and to collaborate closely with the patient's multidisciplinary team, including medical oncologists and head and neck surgeons, to provide the best possible care.
