# Supplementary material for: Benchmarking ChatGPT-4 on a radiation oncology in-training exam and Red Journal Gray Zone cases: potentials and challenges for ai-assisted medical education and decision making in radiation oncology
Source: Front Oncol. 2023 Sep 14;13:1265024. doi: 10.3389/fonc.2023.1265024 (PMC10543650; doi:10.3389/fonc.2023.1265024)
Supplement: Supplementary file 2 [file DataSheet_2.zip › Red journal gray zone evaluation/Case7_with_GPT_response.pdf]

GRAY ZONE

CME ASTRO

INTERNATIONAL JOURNAL OF
RADIATION ONCOLOGY • BIOLOGY • PHYSICS

www.redjournal.org

Mending the Rent: Knife Versus Rays Versus Syringe

Abhinav Dewan, MBBS, DNB Radiotherapy, and Swarupa Mitra, MBBS, MD Radiotherapy

Department of Radiation Oncology, Rajiv Gandhi Cancer Institute and Research Centre, New Delhi, India

A 60-year-old woman with hypertension presented with postmenopausal bleeding, pain in lower abdomen, and urinary incontinence of 1-month duration. Obstetric (P2L2) and menopausal history were unremarkable. Clinical examination revealed a firm growth involving the cervix and anterior vaginal wall up to the introitus with bilateral medial parametrial involvement. Foul-smelling vaginal discharge was noted. Cervical biopsy revealed poorly differentiated squamous cell carcinoma. Staging magnetic resonance imaging (Fig. 1a) revealed a cervix lesion (8.0 × 4.3 × 4.0 cm) involving the anterior/posterior cervical lips and extending superiorly to the lower uterine corpus and inferiorly to the proximal two-thirds of the vagina with parametrial extension, involvement of the posterior bladder wall, and enlarged pelvic/paraortic nodes. Positron emission tomography-computed tomography (Fig. 1b) showed a metabolically active soft tissue cervical mass (4.5 × 5.0 × 6.9 cm; SUVmax, 12.9) involving the lower uterine body, left parametrium, upper vagina, posterior urinary bladder, and anterior rectal wall with enlarged pelvic/paraortic nodes. Cystoscopy revealed bladder-trigone indentation by cervical growth, and biopsy was suggestive of bladder involvement. The patient received extended-field radiation therapy with concurrent weekly cisplatin to a dose of 45 Gy in 25 fractions to the whole pelvis and paraortic region with simultaneous integrated boost to a dose of 55 Gy in 25 fractions to gross nodes. Magnetic resonance imaging of the pelvis (after chemoradiation) (Fig. 2) showed partial regression of the cervical mass (2.9 Antero-posterior × 3.6 Transverse × 2.2 Craniocaudal cm) involving the posterior lip/upper vagina, bilateral parametrium, and posterior urinary bladder wall with sloughing off in the midline upper vaginal region causing direct communication between the vagina and urinary bladder lumen with complete regression of lymph nodes.

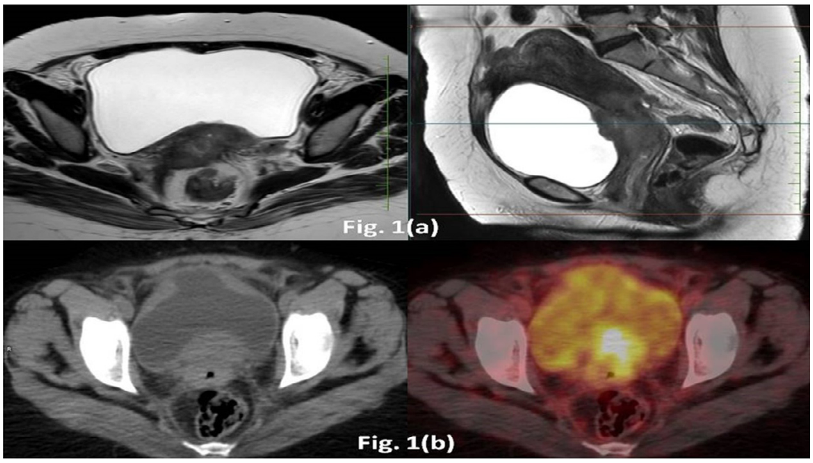

Fig. 1. **(a)** Axial and sagittal T2 imaging shows a cervix mass abutting the posterior urinary bladder wall with fat-plane loss. **(b)** Positron emission tomography/computed tomography showed metabolically active locally advanced cervical carcinoma.

Int J Radiation Oncol Biol Phys, Vol. 113, No. 4, pp. 718−719, 2022
0360-3016/$ - see front matter © 2021 Elsevier Inc. All rights reserved.
https://doi.org/10.1016/j.ijrobp.2021.03.010

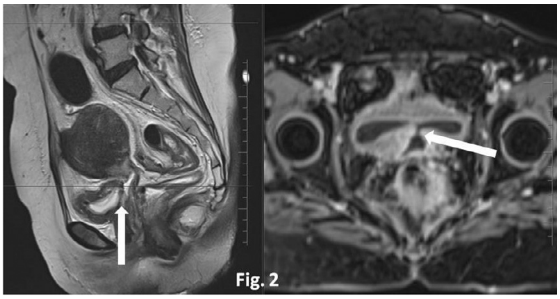

Fig. 2. Post-Chemoradiation Imaging: Sagittal T2 imaging shows a small focal collection abutting the bladder wall, with loss of fat planes. Corresponding post-contrast images show a fistulous tract.

1. How would you manage this patient further?
2. Is there an option of pelvic exenteration, palliative chemotherapy, interstitial brachytherapy implant, or best supportive care?

*Corresponding author: Abhinav Dewan, MBBS, DNB Radiotherapy; E-mail: drabhinavdewan@gmail.com
Disclosures: none.

INTERNATIONAL JOURNAL OF
RADIATION ONCOLOGY • BIOLOGY • PHYSICS
www.redjournal.org

GRAY ZONE EXPERT OPINIONS

Mend and Cure with Surgery

In stage IVA cervical cancer, both primary pelvic exenteration and chemoradiation have almost equal oncologic outcomes. However, exenteration is not usually done because of quality-of-life issues compared with chemoradiation.[1] In this woman, after pelvic radiation with concurrent chemotherapy, there is significant residual disease and a vesicovaginal fistula (VVF) owing to breakdown of cancer-bearing tissues, albeit in response to treatment.

Brachytherapy is not recommended in patients who develop VVF and have residual disease.[2] VVF after chemoradiation is due to endarteritis. Flaps to close the fistula have higher failure rates compared with unirradiated tissue. Hence, in this woman with residual disease in cervix, vagina, and bladder with a VVF, diversion surgery (preferably anterior en bloc resection [anterior pelvic exenteration])[3] is preferrable. The intent of treatment will still be curative at this point.

The role of chemotherapy will depend on surgical margins. If positive, platinum-based chemotherapy may be indicated. Palliative chemotherapy without resection or urinary diversion will lead to ascending infections and poor tolerance. If she is not fit for exenteration, urinary diversion will be needed.

Venkata Pradeep Babu Koyyala, MD, DNB
Department of Medical Oncology
Homi Bhaba Cancer Hospital and Research Centre
Visakhapatnam, Andhra Pradesh, India

Disclosures: none.

References

1. Marnitz S, Köhler C, Müller M, et al. Cervical cancer on stage FIGO IVA: Chemoradiation or exenteration? *Int J Radiat Oncol Biol Phys* 2007;69:S404.
2. Deewan A, Mitra S. Mending the rent: Knife versus rays versus syringe. *Int J Radiat Oncol Biol Phys* 2022;113:710–711.
3. Li L, Ma SQ, Tan XJ, et al. Pelvic exenteration for recurrent and persistent cervical cancer. *Chin Med J* 2018;131:1541–1548.

https://doi.org/10.1016/j.ijrobp.2021.03.008

Rays Over Knife

For stage IVA cervical cancer, the gold standard is concurrent chemoradiation followed by image-guided brachytherapy, including in the presence of a fistula which is not a contraindication for brachytherapy. Given the presence of vesicovaginal fistula, this patient should undergo placement of bilateral percutaneous nephrostomy tubes prior to treatment.

We would proceed with perineal interstitial HDR brachytherapy in order to cover the residual disease.[1] A preplanning magnetic resonance imaging (MRI) with vaginal gel and an MRI or computed tomography (CT) with cylinder in place can be performed to guide needle position and depth. Intraoperatively, the uterine cavity is dilated to allow tandem insertion under transabdominal ultrasound guidance. A cylinder with perineal template and interstitial catheters are placed under ultrasound guidance with particular attention to anterior bladder coverage. The ultrasound can be placed in the vagina first for anterior needle placement. The ultrasound can be utilized transrectally for the paravaginal/parametrial needles. After applicator placement, the patient ideally would undergo MRI-simulation. If MRI-simulation is not available, we recommend fusion of CT simulation with pretreatment MRI with cylinder in place. The high risk clinical target volume will include the gross disease (vagina, parametria and bladder), entire cervix, and gray areas on T2W MRI. A dose of least 85 Gy equivalent dose in 2 Gy fractions to high risk clinical target volume D90 (minimum dose covering 90% of the volume) should be delivered with D98 to the gross tumor volume of 95 Gy equivalent dose in 2 Gy fractions.

If bladder involvement cannot be covered appropriately with definitive doses (even if a reimplant were to be

0360-3016/$ - see front matter © 2021 Elsevier Inc. All rights reserved.

attempted), we recommend aborting the procedure and referring the patient for salvage pelvic exenteration which is the gold standard for persistent and recurrent disease after definitive chemoradiation. Chemotherapy in lieu of exenteration can be considered in the palliative setting after exhaustion of all curative approaches. We would also consider enrolling the patient on a clinical trial.

Lara Hathout, MD
Department of Radiation Oncology
Rutgers Cancer Institute of New Jersey
New Brunswick, NJ

Matthew M. Harkenrider, MD
Department of Radiation Oncology
Stritch School of Medicine
Cardinal Bernardin Cancer Center
Loyola University Chicago
Maywood, Illinois

Disclosures: none.

Reference

1. Deewan A, Mitra S. Mending the rent: Knife versus rays versus syringe. *Int J Radiat Oncol Biol Phys* 2022;113:710–711.

https://doi.org/10.1016/j.ijrobp.2022.04.013

Combination of Knife and Rays with MRI-Guided Ablative Brachytherapy

The authors describe a complex case of locally advanced cervix cancer.[1] Imaging and examination were concerning for extensive disease with involvement of bladder and anterior rectal wall along with pelvic and para-aortic lymph nodes. For this reason, the patient was treated with definitive chemoradiation (external beam radiation therapy, EBRT) to the pelvic and para-aortic region with simultaneous integrated boost to the involved nodes. Positron emission tomography/computed tomography (CT) and magnetic resonance imaging (MRI) after EBRT showed good imaging response with residual disease in cervix with vesicovaginal fistula and regression of all enlarged nodes.

Patients with such advanced locoregional disease are at high risk for both local and distant disease progression after completion of definitive therapy. EMBRACE-I results published in 2021 give us insight on outcomes for IVA cervical cancer patients treated with definitive chemoradiation with MRI-based brachytherapy boost in the modern era.[2] In this study, 2.5% (34 patients) enrolled had IVA disease and had a 5-year pelvic control rate of 81%. These patients were at elevated risk of developing fistula and ureteral strictures (18.6% [n = 6]; 21.3% [n = 23], respectively). Similarly in another series of CT-based interstitial brachytherapy, 8 patients had stage IVA disease with crude local control and vesicovaginal fistula being 62.5 % and 12.5% respectively.[3] Furthermore, patients with IVA disease, are at elevated risk for developing distant disease, also with the EMBRACE series showing 5-year disease-free survival and overall survival of 47% and 52%, respectively.[2] Also, in patients with para-aortic nodal disease at presentation like the case here there is increased rate of distant disease, which adversely impacts their outcomes with 5 year disease-free survival and overall survival in the range of 25% to 50%

Based on this information, we recommend the patient complete her definitive radiation with image-based brachytherapy preferably with MRI guidance as there would be better lineation of target volume in the setting of fistula and residual disease seen on imaging. We would recommend brachytherapy boost with the hybrid applicator with vaginal cap to cover vaginal disease and template to cover parametrial disease to deliver EQD2 dose of 85 to 90 Gy to HRCTV (to include the entire cervix and adjacent residual disease including gray zones) and D98 of >95 Gy to residual GTV seen on MRI.

Because this patient has developed vesicovaginal fistula on treatment, we favor bilateral nephrostomy now to divert urine to help with symptoms caused by constant leakage. About 12 to 16 weeks after completion of brachytherapy would perform positron emission tomography/CT along with MRI imaging with functional sequences to assess for locoregional response and any new distant metastases.[4,5] Should the patient have evidence of complete response on pelvic examination and imaging, we would then favor replacement of bilateral nephrostomy urinary diversion with ileal conduit as more definitive treatment to manage symptoms from fistula. These vesicovaginal fistulas are unlikely to heal and very hard to repair even after complete regression of disease. Should the patient have biopsy proven persistent disease at 12 to 16 weeks then an informed risks and benefits discussion should occur with the patient for palliative systemic treatment or salvage exenteration. She should understand that despite aggressive surgical salvage with associated morbidities, she is at elevated risk of failing distantly due to extent of nodal disease at presentation.

Parul Barry, MD, FACRO
Hillman Cancer Center, Radiation Oncology
University of Pittsburgh Medical Center
Pittsburgh, Pennsylvania

Sushil Beriwal, MD, MBA, FABS, FASTRO
Allegheny Health Network, Academic Chief
Pittsburgh, Pennsylvania
Varian Medical Systems
Vice President, Medical Affairs
Palo Alto, CA

Disclosures: P.B. is an Elsevier Pathways consultant and S.B. is Elsevier Pathways Director. S.B.'s employer is Varian, and he serves as Academic Chief of AHN. He serves on the Xoft data and safety monitoring board. P.B. is employed by UPMC Hillman Cancer Center and serves as an ACRO Board member.

References

1. Deewan A, Mitra S. Mending the rent: Knife versus rays versus syringe. *Int J Radiat Oncol Biol Phys* 2022;113:710–711.
1. Pötter R, Tanderup K, Schmid MP, et al. MRI-guided adaptive brachytherapy in locally advanced cervical cancer (EMBRACE-I): A multicentre prospective cohort study. *Lancet Oncol* 2021;22:538–547.
2. Kannan N, Beriwal S, Kim H, et al. High-dose-rate interstitial computed tomography-based brachytherapy for the treatment of cervical cancer: Early results. *Brachytherapy* 2012;11:408–412.
3. Schwarz JK, Siegel BA, Dehdashti F, Grigsby PW. Association of post-therapy positron emission tomography with tumor response and survival in cervical carcinoma. *JAMA* 2007;298:2289–2295.
4. Kalash R, Glaser SM, Rangaswamy B, et al. Use of functional magnetic resonance imaging in cervical cancer patients with incomplete response on positron emission tomography/computed tomography after image-based high-dose-rate brachytherapy. *Int J Radiat Oncol Biol Phys* 2018;102:1008–1013.

https://doi.org/10.1016/j.ijrobp.2022.04.017

ChatGPT-4: Combination of Knife and Syringe

User: *You are an expert radiation oncologist from an academic center.*

Please give your recommendation on the following case:

"…"

Please give a specific recommendation of your most favored therapeutic approach in this patient case based on the available information and your reasoning for it.

ChatGPT-4: Based on the available information, this patient has a locally advanced, poorly differentiated squamous cell carcinoma of the cervix with bladder involvement and metastatic lymph nodes. The patient has already received extended-field radiation therapy with concurrent weekly cisplatin to a dose of 45 Gy in 25 fractions to the whole pelvis and para-aortic region with simultaneous integrated boost to a dose of 55 Gy in 25 fractions to gross nodes. Post-treatment imaging showed partial regression of the cervical mass and complete regression of lymph nodes, but a fistulous tract between the vagina and urinary bladder has developed.

Considering the treatment response and the presence of a fistula, my most favored therapeutic approach for this patient would be a combination of salvage surgery and adjuvant therapy. The specific recommendations are as follows:

Salvage surgery: Given the remaining cervical mass and the fistula, I would recommend a radical hysterectomy with bilateral salpingo-oophorectomy, partial vaginectomy, and partial cystectomy, along with repair of the fistulous tract. This surgery aims to remove the remaining tumor and address the fistula.

Adjuvant therapy: After the surgery, I would recommend adjuvant chemotherapy, preferably with a combination of cisplatin and paclitaxel or carboplatin and paclitaxel, depending on the patient's tolerance and previous response to cisplatin. This adjuvant therapy is intended to target any residual microscopic disease and decrease the risk of recurrence.

The rationale for this approach is that the patient has already received a course of chemoradiation with partial tumor response, but the fistula complicates the situation and can severely affect the patient's quality of life. Salvage surgery offers a chance to remove the remaining tumor, repair the fistula, and potentially improve the patient's quality of life. Adjuvant chemotherapy will help address any residual disease and reduce the risk of recurrence.

Of course, the patient's performance status, comorbidities, and preferences should be taken into account when discussing these options. A multidisciplinary team involving a gynecologic oncologist, radiation oncologist, and medical oncologist should review the case and determine the best course of action tailored to the patient's individual circumstances.

User: *How would you manage this patient further?*

ChatGPT-4: After implementing the recommended salvage surgery and adjuvant chemotherapy, the patient would require a comprehensive follow-up plan to monitor for potential recurrence, manage treatment-related complications, and provide supportive care. Here's a suggested follow-up and management plan:

- Post-operative care and monitoring: Ensure appropriate post-operative care, including pain management, wound care, and monitoring for any potential complications such as infection, bleeding, or urinary incontinence.

- Follow-up imaging and exams: Schedule regular follow-up visits with pelvic examinations, Pap smears, and imaging studies (e.g., CT or MRI) every 3-6 months for the first two years, and then every 6-12 months for the next three years. After five years, annual follow-ups should be sufficient if the patient remains free of recurrence. These follow-ups will help detect any signs of recurrence or metastasis early.

- Monitoring of treatment-related side effects: Assess and manage any long-term side effects from surgery, radiation, and chemotherapy, such as lymphedema, bowel or bladder dysfunction, and sexual health concerns.

- Supportive care: Address the patient's psychosocial and emotional well-being by providing access to support groups, counseling, or other resources. Encourage the patient to maintain a healthy lifestyle through regular exercise, a balanced diet, and stress reduction techniques.

- Personalized survivorship care plan: Develop a personalized survivorship care plan for the patient to provide guidance on follow-up care, healthy lifestyle recommendations, and possible late effects of treatment. This plan should be updated periodically based on the patient's needs and any new medical information.

- Collaboration with other healthcare providers: Coordinate care with the patient's primary care physician, gynecologist, and any other relevant healthcare providers to ensure seamless management of the patient's overall health and to address any comorbid conditions, such as hypertension.

Ultimately, the goal of further management is to closely monitor the patient's progress, promptly address any complications, and provide comprehensive supportive care to ensure the best possible long-term outcomes and quality of life.

User: *Is there an option of pelvic exenteration, palliative chemotherapy, interstitial brachytherapy implant, or best supportive care?*

ChatGPT-4: Yes, these options can also be considered depending on the patient's specific circumstances, preferences, and overall health. Here is an overview of each approach:

Pelvic exenteration: This is a radical surgical procedure involving the removal of multiple organs in the pelvis, such as the bladder, uterus, cervix, vagina, and sometimes the rectum, depending on tumor involvement. Pelvic exenteration may be considered in cases where the tumor is locally advanced and involves several pelvic organs, or when other surgical or non-surgical treatments have not been successful. It can offer the possibility of long-term disease control or even cure. However, this surgery is associated with significant morbidity and long-term effects on the patient's quality of life. It is crucial to carefully weigh the potential benefits against the risks and impact on the patient's well-being.

Palliative chemotherapy: If the patient is not a candidate for further aggressive treatment due to poor performance status, significant comorbidities, or other factors, palliative chemotherapy can be considered to manage symptoms and potentially slow tumor progression. The choice of chemotherapy agents would be based on the patient's previous response to cisplatin, tolerance to chemotherapy, and the overall goal of maintaining the best possible quality of life.

Interstitial brachytherapy implant: Brachytherapy involves the placement of radioactive material directly into or near the tumor. In this case, interstitial brachytherapy could be an option for treating residual or recurrent tumor, particularly if the patient is not a candidate for further surgery or external beam radiation therapy. This treatment delivers a high dose of radiation directly to the tumor while minimizing radiation exposure to surrounding healthy tissue.

Best supportive care: If the patient is not a candidate for further aggressive treatment or prefers not to pursue additional interventions, the focus should shift to best supportive care. This approach emphasizes managing symptoms, maintaining the patient's comfort and quality of life, and providing emotional and psychological support. It includes pain management, nutritional support, and other palliative measures tailored to the patient's specific needs.

A multidisciplinary team should discuss these options with the patient, taking into account her preferences, overall health, and the potential benefits and risks associated with each treatment modality. The goal is to develop a personalized plan that balances the potential for disease control with the patient's quality of life.

***User:** Please summarize the other experts' recommendations in short sentences.*

ChatGPT-4: Expert 1: Recommends en bloc resection (anterior pelvic exenteration) with the intent of curative treatment, followed by chemotherapy if margins are positive. Urinary diversion is needed if the patient is not fit for exenteration.

Expert 2: Advocates for perineal interstitial HDR brachytherapy to cover residual disease, with bilateral percutaneous nephrostomy tubes placed prior to treatment. If bladder involvement cannot be covered, consider salvage pelvic exenteration or palliative chemotherapy.

Expert 3: Suggests completing definitive radiation with MRI-guided brachytherapy, then assessing response 12-16 weeks after. If there's a complete response, proceed with urinary diversion using an ileal conduit. If persistent disease is present, discuss palliative systemic treatment or salvage exenteration with the patient.

User: *As an expert radiation oncologist, which of the above experts' recommendation do you think the most proper?*

ChatGPT-4: As an expert radiation oncologist, I would lean towards Expert 2's recommendation. This approach involves perineal interstitial HDR brachytherapy to cover the residual disease, with bilateral percutaneous nephrostomy tubes placed prior to treatment. This strategy is in line with the standard treatment for stage IVA cervical cancer, which is concurrent chemoradiation followed by image-guided brachytherapy.

The presence of a vesicovaginal fistula in this patient is indeed a challenge, but it is not a contraindication for brachytherapy. By placing bilateral percutaneous nephrostomy tubes before the procedure, the patient's urinary symptoms can be better managed, allowing for a more effective brachytherapy treatment.

However, it is crucial to have a multidisciplinary team evaluate the patient and her preferences, overall health, and the potential benefits and risks associated with each treatment modality. The goal is to develop a personalized plan that balances the potential for disease control with the patient's quality of life.

User: *At the beginning, you said that "my most favored therapeutic approach for this patient would be a combination of salvage surgery and adjuvant therapy. " In this case, which expert's recommendation is your initial suggestion closest to?*

ChatGPT-4: I apologize for the confusion earlier. Upon further consideration of the experts' opinions, my initial suggestion aligns more closely with Expert 1's recommendation. Expert 1 suggests en bloc resection (anterior pelvic exenteration) with the intent of curative treatment, followed by chemotherapy if margins are positive. Urinary diversion is needed if the patient is not fit for exenteration. This approach combines salvage surgery with adjuvant therapy depending on the surgical margins.

However, as emphasized earlier, it is essential to have a multidisciplinary team evaluate the patient, taking into account her preferences, overall health, and the potential benefits and risks associated with each treatment modality. The goal is to develop a personalized plan that balances the potential for disease control with the patient's quality of life.

User: *After seeing the other experts' recommendations, are you going to update your own recommendation of your most favored therapeutic approach in this patient case? If so, what is your updated recommendation and which expert/experts is your update based on?*

ChatGPT-4: After reviewing the other experts' recommendations, I would like to update my initial recommendation to align more closely with Expert 2's approach. Expert 2 suggests perineal interstitial HDR brachytherapy to cover the residual disease, with bilateral percutaneous nephrostomy tubes placed prior to treatment. This strategy aligns with the standard treatment for stage IVA cervical cancer, which is concurrent chemoradiation followed by image-guided brachytherapy.

The presence of a vesicovaginal fistula in this patient presents a challenge, but it is not a contraindication for brachytherapy. By placing bilateral percutaneous nephrostomy tubes before the procedure, the patient's urinary symptoms can be better managed, allowing for a more effective brachytherapy treatment.

It remains crucial to have a multidisciplinary team evaluate the patient, taking into account her preferences, overall health, and the potential benefits and risks associated with each treatment modality. The goal is to develop a personalized plan that balances the potential for disease control with the patient's quality of life.
