# Supplementary material for: Benchmarking ChatGPT-4 on a radiation oncology in-training exam and Red Journal Gray Zone cases: potentials and challenges for ai-assisted medical education and decision making in radiation oncology
Source: Front Oncol. 2023 Sep 14;13:1265024. doi: 10.3389/fonc.2023.1265024 (PMC10543650; doi:10.3389/fonc.2023.1265024)
Supplement: Supplementary file 2 [file DataSheet_2.zip › Red journal gray zone evaluation/Case8_with_GPT_response.pdf]

GRAY ZONE

CME ASTRO

INTERNATIONAL JOURNAL OF
RADIATION ONCOLOGY • BIOLOGY • PHYSICS

www.redjournal.org

A Viewpoint on Isolated Contralateral Axillary Lymph Node Involvement by Breast Cancer: Regional Recurrence or Distant Metastasis?

Abdulla Al-Rashdan, MD,[*,†] and Jeffery Cao, MD, MBA[*,†]

[*]Department of Oncology, Division of Radiation Oncology, University of Calgary, Alberta, Canada; and [†]Tom Baker Cancer Centre, Calgary, Alberta, Canada

Received Aug 1, 2020; Accepted for publication Sep 16, 2020

A 55-year-old woman was diagnosed in 2010 with mixed invasive lobular and ductal carcinoma of the left breast. Initial breast conserving surgery and sentinel lymph node biopsy revealed 2.2 cm (lobular) and 0.4 cm (ductal), grade 2, lymphovascular invasion and surrounding lobular carcinoma in situ and ductal carcinoma in situ but negative margins for invasive component. One of 1 sentinel node was positive. Subsequent mastectomy and axillary lymph node dissection revealed residual ductal carcinoma in situ, and 0 of 20 nodes were involved. Final stage was pT2N1a. Estrogen and progesterone receptors were positive but human epidermal growth factor receptor 2 was unknown. Patient received anthracycline/taxane chemotherapy and tamoxifen but discontinued due to intolerance. Adjuvant radiation therapy was not recommended. The patient underwent right contralateral prophylactic mastectomy with implant reconstruction in 2012.

In 2020, 2 firm nodes were identified in the right axilla (contralateral to the original primary) and removed along with another adjacent node in a limited resection. Pathology confirmed metastatic breast (mixed ductal and lobular) carcinoma involving 2 of 3 lymph nodes, tumor deposits measuring 0.8 cm and 1.9 cm, both showing extranodal extension, large vessel, and perineural invasion seen in perinodal soft tissue. Estrogen and progesterone were positive and human epidermal growth factor receptor 2 was negative. No primary tumor was found on resection of implants and capsules bilaterally. No distant metastasis was evidenced on restaging.

- Would you offer radiation to the right side and what would be your target volumes?
- Would your decision be affected by systemic therapy or extent of axillary surgery?
- Would you consider radiation therapy to the original left side?

*Corresponding author: Abdulla Al-Rashdan, MD; E-mail: Abdullah.Al-Rashdan@Albertahealthservices.ca
Disclosures: none.

Int J Radiation Oncol Biol Phys, Vol. 113, No. 3, pp. 489, 2022
0360-3016/$ - see front matter © 2020 Elsevier Inc. All rights reserved.
https://doi.org/10.1016/j.ijrobp.2020.09.036

INTERNATIONAL JOURNAL OF
RADIATION ONCOLOGY • BIOLOGY • PHYSICS
www.redjournal.org

GRAY ZONE EXPERT OPINION

Contralateral Disease Begets Contralateral Treatment

Isolated metachronous contralateral axillary lymph node metastasis (CAM) is rare. Potential etiologies include (1) contralateral spread from the original ipsilateral cancer, (2) contralateral spread from new or recurrent ipsilateral tumor, and (3) ipsilateral spread from new contralateral disease. CAM is associated with aggressive biology but may represent regional recurrence from aberrant lymphatic drainage after axillary surgery and not distant hematogenous recurrence, warranting aggressive local therapy.

In the presented case,[1] metachronous CAM with histology concordant with the original disease is suggestive of subclinical contralateral spread from the left primary. Aggressive treatment with radiation therapy and systemic therapy may improve progression-free survival.[2] Regardless, the risk of right nodal recurrence is high given extranodal extension and perineural invasion and warrants right postmastectomy radiation therapy (PMRT) treating the chest wall and regional nodes (axillary, supraclavicular, and internal mammary). This may also address occult right-sided lesions and dermal lymphatics that can transmit left-sided disease.

Right PMRT is recommended regardless of systemic therapy or axillary surgery, especially given the locoregional risk from extranodal extension and perineural invasion. Systemic therapy is encouraged to mitigate distant recurrence risk and maximize PFS and overall survival.[3] Axillary dissection may not be of benefit and does not obviate PMRT.[1]

Radiation therapy to the left side can be omitted because there is no gross disease currently, nor for the past 10 years, and the left axilla was therapeutically dissected; this avoids the risks of PMRT after extensive surgery, namely lymphedema. Overall, an interdisciplinary approach should be used to address regional and distant recurrence risk.

Nicolas D. Prionas, MD, PhD
Catherine C. Park, MD
Department of Radiation Oncology
UCSF Helen Diller Family Comprehensive Cancer Center
University of California
San Francisco, California

Disclosures: none.

References

1. Al-Rashdan A, Cao J. A Viewpoint on isolated contralateral axillary lymph node involvement by breast cancer: regional recurrence or distant metastasis? *Int J Radiat Oncol Biol Phys* 2022;113:489.
2. Wang W, Yuan P, Wang J, et al. Management of contralateral axillary lymph node metastasis from breast cancer: A clinical dilemma. *Tumori* 2014;100:600–604.
3. Magnoni F, Colleoni M, Mattar D, et al. Contralateral axillary lymph node metastases from breast carcinoma: Is it time to review TNM cancer staging? *Ann Surg Oncol* 2020;27:4488–4499.

https://doi.org/10.1016/j.ijrobp.2020.11.033

Long Overdue "Beam-On"

At the time of initial breast cancer diagnosis,[1] this patient underwent suboptimal locoregional and systemic treatment due to omission of postmastectomy regional nodal irradiation (RNI) and inadequate duration of antiestrogen therapy. Although the benefits of RNI for patients with limited nodal disease were not fully appreciated in the past, subsequent clinical trials and a meta-analysis have demonstrated a clear benefit to RNI with an estimated ~3% to 4% absolute reduction in distant metastases.[2]

After 10 years, the patient now has contralateral axillary nodal metastases. In addition to restaging studies, we recommend bilateral magnetic resonance imaging to assess for potential primary/recurrent tumor in the residual ipsilateral or contralateral breast tissue. Because aberrant lymphatic drainage to the contralateral axilla is not uncommon after axillary lymph node dissection, and in the absence of distant metastases, the contralateral axillary nodal metastases likely represent regional spread of disease or could be the result of an occult primary tumor in the residual right breast tissue. Although data are limited, some patients with contralateral axillary nodal metastases may be cured with aggressive locoregional treatment.[3]

Because both scenarios (regional spread from the left breast or occult primary in the right breast) are potentially amenable to long-term disease control with aggressive locoregional therapy, we would recommend bilateral comprehensive radiation therapy to the chest wall and RNI. Our RNI target volumes

Int J Radiation Oncol Biol Phys, Vol. 113, No. 3, pp. 490−492, 2022
0360-3016/$ - see front matter © 2020 Elsevier Inc. All rights reserved.

would include the undissected axillary nodal basins, supraclavicular nodes, and internal mammary nodes. Given the high risk of subsequent distant hematogenous metastases, discussion of additional systemic therapy is warranted.

Youssef H. Zeidan, MD, PhD
American University of Beirut and Lynn Cancer Institute
Boca Raton, Florida

Jose G. Bazan, MD
Department of Radiation Oncology
Arthur G. James Comprehensive Cancer Center
Ohio State University
Columbus, Ohio

Disclosures: none

References

1. Al-Rashdan A, Cao J. A Viewpoint on isolated contralateral axillary lymph node involvement by breast cancer: regional recurrence or distant metastasis? *Int J Radiat Oncol Biol Phys* 2022;113:489.
2. Whelan TJ, Olivotto IA, Parulekar WR, et al. Regional nodal irradiation in early-stage breast cancer. *N Engl J Med.* 2015;373(4):307–316.
3. Chkheidze R, Sanders MAG, Haley B, Leitch AM, Sahoo S. Isolated contralateral axillary lymph node involvement in breast cancer represents a locally advanced disease not distant metastases. *Clin Breast Cancer* 2018;18:298–304.

https://doi.org/10.1016/j.ijrobp.2021.07.1704

Making the Right Choice: Radiate Only What's Left, the Rest is Left

Although the exact origin will never be known, we favor regional recurrence of original tumor rather than new primary or distant metastasis.[1] Recurrent breast cancers are known to have aberrant nodal drainage, including to the contralateral axilla,[2] and similarity of histology to the original diagnosis suggests a recurrence of the original tumor. In addition, exhaustive efforts were undertaken, including excision of implants (and assuming imaging including breast magnetic resonance imaging and positron emission tomography/computed tomography) to look for occult primary and distant metastases.

We would radiate the right-sided axillary levels 1 to 3 and supraclavicular (SCV) nodes only, 50 Gy in 25 fractions targeting the potential microscopic disease remaining after surgery. Historically, most patients in this scenario had axillary lymph node dissection, but some have suggested that radiation therapy, rather than extent of surgery, may be more important.[2,3] Thus, the extent of axillary surgery is up to the surgeon and would not change our radiation field in this patient with higher-risk features for regional progression such as extracapsular extension and lymphovascular invasion.[4] Because there is no evidence of chest wall or left-sided nodal recurrence 10 years after treatment, we would omit irradiation of the bilateral chest walls and left axilla/SCV to reduce morbidity, as potential future isolated recurrence is salvageable, and the patient is just as likely to develop distant disease.

Systemic therapy is critical in reducing the chance of metastases, and should include endocrine and chemotherapy, and possibly a cyclin-dependent kinase 4/6 inhibitor. However, systemic therapy would not affect our radiation recommendations aside from starting radiation after any cytotoxic chemotherapy. Finally, this is a great case that illustrates the importance of long-term follow up in breast cancer patients, both on and off-protocol. We wonder whether the outcome would have been different if the patient had radiation at initial diagnosis.

Diane C. Ling, MD
Jason C. Ye, MD
Department of Radiation Oncology
Keck School of Medicine of USC
Los Angeles, California

Disclosures: none.

References

1. Al-Rashdan A, Cao J. A Viewpoint on isolated contralateral axillary lymph node involvement by breast cancer: regional recurrence or distant metastasis? *Int J Radiat Oncol Biol Phys* 2022;113:489.
2. Moossdorff M, Vugts G, Maaskant-Brant AJG, et al. Contralateral lymph node recurrence in breast cancer: Regional event rather than distant metastatic disease. A systematic review of the literature. *Eur J Surg Oncol* 2015;41:1128–1136.
3. Wang W, Yuan P, Wang J, et al. Management of contralateral axillary lymph node metastasis from breast cancer: A clinical dilemma. *Tumori* 2014;100:600–604.
4. Fisher BJ, Perera FE, Opeitum A, et al. Extracapsular axillary node extension in patients receiving adjuvant systemic therapy: An indication for radiotherapy? *Int J Radiat Oncol Biol Phys* 1997;38:551–559.

https://doi.org/10.1016/j.ijrobp.2022.04.002

Postoperative Comprehensive Radiation with Curative Intent

Isolated metachronous contralateral axillary metastases (CAM) from breast carcinoma are rare and historically classified as distant metastatic disease.[1] However, altered lymphatic drainage patterns are common after mastectomy, particularly after axillary lymph node dissection,[2] where spread to the contralateral axilla occurs via drainage through superficial dermal lymphatics or deep facial plexus. Patients with isolated CAM have improved prognosis compared with those with distant metastasis, and many advocate for classifying isolated CAM as a regional recurrence event.[3]

Given negative systemic staging, we favor proceeding with curative intent treatment (provided this approach aligns with the patient's goals) consisting of possible axillary node dissection (depending on patient wishes and surgical recommendations), adjuvant radiation therapy, subsequent aromatase inhibitor, and likely CDK 4/6 inhibitor.

Although occult CAM at initial diagnosis with subsequent indolent growth is possible, occult ipsilateral recurrence with spread to the contralateral axilla seems more likely given lymphovascular invasion and nodal involvement at diagnosis. Tailoring to the latter possibility, we would target bilateral chest walls, and bilateral lymph node areas at risk including right axilla, left undissected axilla, and bilateral internal mammary and supraclavicular nodes. Fifty Gy in 25 fractions would be given using a central electron IMN field matched to bilateral photon tangents. To minimize cardiac dose, the central electron field would be split with higher energy superiorly and lower energy inferiorly, in addition to use of deep inspiratory breath hold. In the absence of an axillary dissection, we would boost any undissected locoregional adenopathy seen on imaging to 60 to 66 Gy pending evaluation of brachial plexus dose.

Susan G.R. McDuff, MD, PhD
Rachel C. Blitzblau, MD, PhD
Department of Radiation Oncology
Duke University
Durham, NC

Disclosures: none.

References

1. Al-Rashdan A, Cao J. A Viewpoint on isolated contralateral axillary lymph node involvement by breast cancer: regional recurrence or distant metastasis? *Int J Radiat Oncol Biol Phys* 2022;113:489.
2. Maaskant-Braat AJG, Roumen RMH, Voogd AC, et al. Sentinel node and recurrent breast cancer (SNARB): Results of a nationwide registration study. *Ann Surg Oncol* 2013;20:620–626.
3. Wang W, Yuan P, Wang J, et al. Management of contralateral axillary lymph node metastasis from breast cancer: A clinical dilemma. *Tumori* 2014;100:600–604.

https://doi.org/10.1016/j.ijrobp.2022.04.007

A Favorable Entity Warranting the Right Therapy

This vignette[1] describes a patient with an ER/PR-positive, HER-2-negative, pT2N1a, mixed histology left breast carcinoma presenting 10 years after initial diagnosis with an isolated contralateral axillary metastasis (CAM). Next steps should include thorough multidisciplinary review. Completion axillary lymph node dissection and a retrial of hormone therapy are strongly recommended.

Although traditional viewpoints would suggest palliation, contemporary evidence has identified CAM as a distinct entity with favorable outcomes in comparison to other areas of dissemination.[2] Further, her molecular subtype and prolonged trajectory from diagnosis to recurrence suggest a favorable biology. These factors warrant the use of aggressive salvage therapy with the goal of improving survival and maximizing local control.

Right-sided locoregional radiation therapy would be recommended. In many Canadian or UK centers, this would entail a hypofractionated dose (42.5 Gy/16 or 40 Gy/15) daily to the chest wall and regional lymph nodes. Full coverage of nodal levels, including the internal mammary nodes, would be encouraged given the concerning pathologic features, although a modified volume could be considered based on results of the axillary lymph node dissection. The provision of chemotherapy would not alter this recommendation.

CAM likely results from aberrant drainage secondary to blockage of normal lymphatic routes,[3] providing some slightly controversial rationale for radiating the original left side. However, for this patient the toxicity of radiation therapy to this area, although minimal, would likely outweigh the benefit.

This case highlights the exciting, everchanging landscape of metastatic breast cancer, which requires comprehensive clinical consideration to ensure high-quality patient-centered care.

Nafisha Lalani, MD, MPH
Irving Greenberg Family Cancer Centre
University of Ottawa
Ottawa, Canada

Disclosures: none.

References

1. Al-Rashdan A, Cao J. A Viewpoint on isolated contralateral axillary lymph node involvement by breast cancer: regional recurrence or distant metastasis? *Int J Radiat Oncol Biol Phys* 2022;113:449.
2. Magnoni F, Colleoni M, Mattar D, et al. Contralateral axillary lymph node metastases from breast carcinoma: Is it time to review TNM cancer staging? *Ann Surg Oncol* 2020;27:4488–4499.
3. Perre CI, Hoefnagel CA, Kroon BB. Altered lymphatic drainage after lymphadenectomy or radiotherapy of the axilla in patients with breast cancer. *Br J Surg* 1996;83:1258.

https://doi.org/10.1016/j.ijrobp.2022.04.011

ChatGPT-4: Patient's History is as Important as Current Pathology Findings

User: *You are an expert radiation oncologist from an academic center.*

Please give your recommendation on the following case:

"…"

Please give a specific recommendation of your most favored therapeutic approach in this patient case based on the available information and your reasoning for it.

ChatGPT-4: Based on the available information, the patient has locally recurrent breast cancer with contralateral axillary lymph node involvement. Considering the patient's history and pathology findings, my recommendation for the therapeutic approach would include the following:

Systemic therapy: Given that the patient has hormone receptor-positive (ER/PR+) and HER2-negative disease, initiating endocrine therapy with an aromatase inhibitor (AI) would be my first choice. In addition, considering the prior discontinuation of tamoxifen due to intolerance, AI would be a better option for this patient. Additionally, since the patient has locally advanced and recurrent disease, I would also recommend adding a CDK4/6 inhibitor, such as palbociclib, ribociclib, or abemaciclib, in combination with the AI to improve the efficacy of treatment.

Radiation therapy: As the patient has not received radiation therapy in the past and given the involvement of contralateral axillary lymph nodes with extranodal extension, I would recommend considering regional nodal irradiation, which includes the chest wall (in the case of a mastectomy) and regional lymph nodes (axillary, supraclavicular, and internal mammary). This will help in reducing the risk of further local-regional recurrence and may improve overall survival.

Close monitoring: The patient should undergo regular follow-ups with physical examinations, and imaging as needed, to closely monitor her response to the treatment and to detect any signs of disease progression or new metastases.

Supportive care: It is essential to address any potential side effects or complications of the treatments mentioned above, such as fatigue, bone health issues, and lymphedema. This would involve coordinating with a multidisciplinary team of healthcare professionals including medical oncologists, radiation oncologists, surgeons, physical therapists, and other supportive care services.

In summary, my favored therapeutic approach for this patient would be the combination of endocrine therapy with an aromatase inhibitor and a CDK4/6 inhibitor, regional nodal irradiation, close monitoring, and appropriate supportive care. This recommendation is based on the patient's hormone receptor-positive, HER2-negative status, prior treatment history, and the need to address local-regional disease control while minimizing the risk of further recurrence.

User: *Would you offer radiation to the right side and what would be your target volumes?*

ChatGPT-4: Yes, given the presence of metastatic breast cancer in the right axillary lymph nodes, I would recommend radiation therapy to the right side as well. The target volumes for radiation therapy in this case would include:

Right chest wall: As the patient had undergone a contralateral prophylactic mastectomy on the right side, radiation to the right chest wall would be indicated to address any potential microscopic disease that could be present in the chest wall tissues.

Right axillary lymph nodes: Since the patient has confirmed involvement of right axillary lymph nodes, it would be crucial to include this area in the radiation treatment plan to eliminate any residual disease.

Right supraclavicular fossa: The supraclavicular lymph nodes are a common site for breast cancer metastasis. Including the right supraclavicular fossa in the radiation treatment plan would help to address any potential microscopic disease in this region.

Right internal mammary lymph nodes: Although not always included in every breast cancer case, given the locally advanced and recurrent nature of this patient's disease, I would also recommend considering irradiation of the right internal mammary lymph nodes to address any potential microscopic disease in this area.

The radiation therapy should be planned and delivered using modern techniques, such as intensity-modulated radiation therapy (IMRT) or volumetric modulated arc therapy (VMAT), to ensure optimal dose distribution while minimizing the dose to adjacent healthy tissues and critical structures, such as the heart and lungs.

***User:** Would your decision be affected by systemic therapy or extent of axillary surgery?*

ChatGPT-4: My decision regarding radiation therapy would not be significantly affected by the choice of systemic therapy or the extent of axillary surgery. However, there are some considerations that should be taken into account:

Systemic therapy: In this case, the patient would benefit from the combination of endocrine therapy (an aromatase inhibitor) and a CDK4/6 inhibitor. This systemic therapy may provide additional control over the cancer and improve overall outcomes. If the patient were to respond well to systemic therapy, the need for more extensive radiation therapy might be re-evaluated. However, given the locally advanced and recurrent nature of the disease, radiation therapy remains an essential component of the treatment plan to achieve local-regional control.

Extent of axillary surgery: The extent of axillary surgery could have an impact on the decision-making process for radiation therapy. A more extensive axillary lymph node dissection would reduce

the volume of residual disease and lower the risk of regional recurrence. However, considering the presence of extranodal extension, large vessel, and perineural invasion in the patient's pathology report, radiation therapy to the right axillary region would still be recommended to ensure optimal local control, regardless of the extent of axillary surgery.

In conclusion, while systemic therapy and the extent of axillary surgery can influence the decision-making process, the overall recommendation for radiation therapy in this case would remain largely unchanged. The patient's history of locally advanced and recurrent disease, as well as the presence of high-risk features, highlights the importance of a comprehensive treatment approach that includes radiation therapy for optimal local-regional control.

***User:** Would you consider radiation therapy to the original left side?*

ChatGPT-4: Considering the patient's history and the absence of any new evidence of disease on the left side, I would not routinely recommend radiation therapy to the original left side at this time. However, there are certain factors to consider:

The patient never received adjuvant radiation therapy to the left side after her initial breast-conserving surgery and mastectomy. This could potentially increase the risk of local recurrence on the left side.

The current presentation of contralateral axillary lymph node involvement might suggest an aggressive disease pattern. In such cases, one might argue that the left side could be at a higher risk of recurrence and might benefit from radiation therapy.

Given these factors, the decision to offer radiation therapy to the left side should be made on a case-by-case basis after a thorough discussion with the patient and the multidisciplinary team. Factors such as the patient's overall health, tolerance to radiation therapy, potential side effects, and the risk-benefit ratio should be carefully considered.

If it is decided to include the left side in the radiation treatment plan, the target volumes would include the left chest wall and regional lymph nodes, such as the left axillary, supraclavicular, and internal mammary lymph nodes. As always, modern radiation techniques like IMRT or VMAT should be utilized to ensure optimal dose distribution and minimize the dose to healthy tissues and critical structures.

***User:** Please summarize the other experts' recommendations in short sentences.*

ChatGPT-4:

Expert 1: Recommends right postmastectomy radiation therapy (PMRT) to the chest wall and regional nodes, regardless of systemic therapy or axillary surgery. Omission of left-side radiation is suggested.

Expert 2: Suggests bilateral comprehensive radiation therapy to the chest wall and regional nodal irradiation (RNI), with discussion of additional systemic therapy.

Expert 3: Advises radiation to the right-sided axillary levels 1 to 3 and supraclavicular nodes only, with systemic therapy as a critical component for reducing the chance of metastases.

Expert 4: Recommends postoperative comprehensive radiation therapy with curative intent, targeting bilateral chest walls and lymph node areas, alongside subsequent aromatase inhibitor and CDK 4/6 inhibitor.

Expert 5: Favors right-sided locoregional radiation therapy with a hypofractionated dose to the chest wall and regional lymph nodes. The provision of chemotherapy would not alter this recommendation.

***User:** As an expert radiation oncologist, which of the above experts' recommendation do you think the most proper?*

ChatGPT-4: As an expert radiation oncologist, I believe the recommendation provided by Expert 2 is the most appropriate for the patient described above. This recommendation suggests bilateral comprehensive radiation therapy to the chest wall and regional nodal irradiation (RNI), with a discussion of additional systemic therapy.

This approach considers the patient's suboptimal initial treatment and the potential benefits of aggressive locoregional therapy in controlling the disease. By targeting both sides, it addresses the risk of residual disease on the left side and the possibility of aberrant lymphatic drainage after the axillary lymph node dissection. Furthermore, the recommendation includes a thorough assessment for potential primary or recurrent tumors through bilateral magnetic resonance imaging.

Adding systemic therapy to the treatment plan is essential to minimize the risk of distant metastases and maximize progression-free survival and overall survival. The multidisciplinary approach mentioned in this recommendation ensures a comprehensive treatment plan tailored to the patient's needs and overall prognosis.

***User:** At the beginning, you said that "my favored therapeutic approach for this patient would be the combination of endocrine therapy with an aromatase inhibitor and a CDK4/6 inhibitor, regional nodal irradiation, close monitoring, and appropriate supportive care. " In this case, which expert's recommendation is your initial suggestion closest to?*

ChatGPT-4: My initial recommendation aligns most closely with Expert 3's recommendation. Expert 3 suggests radiating the right-sided axillary levels 1 to 3 and supraclavicular nodes only and emphasizes the critical role of systemic therapy in reducing the chance of metastases. This approach focuses on treating the current contralateral axillary nodal metastases and involves the use of endocrine therapy and a CDK4/6 inhibitor as part of the systemic therapy.

While Expert 3's recommendation omits irradiation of the bilateral chest walls and the left axilla/supraclavicular nodes to reduce morbidity, it acknowledges the importance of long-term follow-up and potential salvage treatment in case of isolated recurrence. This recommendation balances the need for effective treatment of the current disease presentation while minimizing potential side effects and considering the patient's overall prognosis.

***User:** After seeing the other experts' recommendations, are you going to update your own recommendation of your most favored therapeutic approach in this patient case? If so, what is your updated recommendation and which expert/experts is your update based on?*

ChatGPT-4: After reviewing the other five experts' recommendations, I would like to update my initial recommendation to provide a more comprehensive approach for the patient.

Updated recommendation:

- Perform thorough imaging, including bilateral magnetic resonance imaging, to assess for potential primary or recurrent tumors in the residual ipsilateral or contralateral breast tissue.
- Administer right-sided locoregional radiation therapy, targeting the right-sided axillary levels 1 to 3 and supraclavicular nodes. Consider bilateral comprehensive radiation therapy if imaging or further assessment indicates the necessity.
- Initiate systemic therapy, including endocrine therapy with an aromatase inhibitor, a CDK4/6 inhibitor, and possibly chemotherapy to mitigate the risk of distant recurrence and maximize progression-free survival and overall survival.
- Conduct close monitoring and long-term follow-up to detect any signs of recurrence or progression early and provide appropriate supportive care.

This updated recommendation combines essential elements from the other experts' suggestions while balancing the need for aggressive treatment and minimizing potential side effects. It provides a comprehensive and multidisciplinary approach tailored to the patient's needs and overall prognosis.
